# RNA-seq validation: software for selection of reference and variable candidate genes for RT-qPCR

**DOI:** 10.1186/s12864-024-10511-y

**Published:** 2024-07-16

**Authors:** Márcio Wilson Dias de Brito, Stephanie Serafim de Carvalho, Maria Beatriz dos Santos Mota, Rafael Dias Mesquita

**Affiliations:** 1 Programa de Pós-graduação em Biologia Computacional e Sistemas, Instituto Oswaldo Cruz, Fundação Oswaldo Cruz, Rio de Janeiro, Brazil; 2 RioGen Tecnologia, Rio de Janeiro, Brazil; 3 Instituto de Bioquímica Médica Leopoldo de Meis, Universidade Federal do Rio de Janeiro, Rio de Janeiro, Brazil; 4 Departamento de Bioquímica, Instituto de Química, Universidade Federal do Rio de Janeiro, Rio de Janeiro, Brazil; 5 Instituto Nacional de Ciência e Tecnologia em Entomologia Molecular, Universidade Federal do Rio de Janeiro, Rio de Janeiro, Brazil

**Keywords:** Gene expression, Reference genes, Transcriptome validation, *Aedes aegypti*, RNA-seq, RT-qPCR

## Abstract

**Background:**

Real-time quantitative PCR (RT-qPCR) is one of the most widely used gene expression analyses for validating RNA-seq data. This technique requires reference genes that are stable and highly expressed, at least across the different biological conditions present in the transcriptome. Reference and variable candidate gene selection is often neglected, leading to misinterpretation of the results.

**Results:**

We developed a software named “Gene Selector for Validation” (GSV), which identifies the best reference and variable candidate genes for validation within a quantitative transcriptome. This tool also filters the candidate genes concerning the RT-qPCR assay detection limit. GSV was compared with other software using synthetic datasets and performed better, removing stable low-expression genes from the reference candidate list and creating the variable-expression validation list. GSV software was used on a real case, an *Aedes aegypti* transcriptome. The top GSV reference candidate genes were selected for RT-qPCR analysis, confirming that eiF1A and eiF3j were the most stable genes tested. The tool also confirmed that traditional mosquito reference genes were less stable in the analyzed samples, highlighting the possibility of inappropriate choices. A meta-transcriptome dataset with more than ninety thousand genes was also processed successfully.

**Conclusion:**

The GSV tool is a time and cost-effective tool that can be used to select reference and validation candidate genes from the biological conditions present in transcriptomic data.

**Supplementary Information:**

The online version contains supplementary material available at 10.1186/s12864-024-10511-y.

## Background

Gene expression analysis is valuable for obtaining information about cellular mechanisms in different biological samples [1]. Quantitative real-time PCR (RT-qPCR) is a widely used technique to access the gene expression of a specific gene. However, the development of high-throughput sequencing (HTS) technology has enabled the analysis of the whole transcriptome of a cell, tissue, or organism [2].

HTS has enabled an exponential increase in sequencing performance and depth [2–5]. RNA sequencing (RNA-seq) has become increasingly popular in expression profiling analyses, generating a large volume of complex data. Therefore, using high computational power and developing bioinformatics tools was necessary to ensure more reliable data management and interpretation [3, 6, 7].

RT-qPCR has high sensitivity, specificity, and reproducibility, making it the gold standard for gene expression analysis and validating transcriptome datasets [8–12]. However, to better understand the data generated by RT-qPCR, it is essential to use reference genes with high and stable expression under various biological conditions [13–18].

Currently, reference genes are usually chosen based on their function. Therefore, housekeeping genes (HK) (e.g., actin and GAPDH) and ribosomal proteins (e.g., RpS7 and RpL32) are the most common choices due to their presumed stable expression [19–21]. Recent work has shown that these genes can be modulated depending on the biological condition, suggesting that it is important to evaluate and select reference genes according to the biological conditions [22]. When a reference gene is correctly selected, errors generated during the RT-qPCR quantification of the genes used for validation are reduced, thus ensuring a more reliable interpretation of the result [8, 15, 18, 23, 24].

Despite its importance, several studies have neglected to select adequate RT-qPCR reference genes for transcriptome validation. The traditionally used genes may not be ideal for the research in question, and the most common problems are low stability and low levels of gene expression [8, 15, 25–27].

The stability of a reference candidate gene can be checked after the RT-qPCR using statistical software such as OLIVER [28], GeNorm [29], NormFinder [23], and BestKeeper [30], which use cycle quantification (Cq) data obtained from the RT-qPCR [8, 16, 31–33]. Additionally, GenExpA software was developed to determine the best validation gene to use from RT-qPCR data [34]. Other methodologies also use a group of candidates to determine the most stable gene [35]. OLIVER and NormFinder software can also analyze microarray data to select validation candidate genes, but OLIVER has reported better results than the others [28]. Both need command-line interaction in the operational system (OLIVER) or R package (NormFinder).

The packages cited above have limitations in analyzing RNA-seq quantification data because they were not developed with this goal in mind. Some can only analyze a small set of genes (GeNorm and BestKeeper), and none filter out stable low-expression genes.

For instance, studies have used RT-qPCR data from *Aedes aegypti* to determine the best reference gene at different developmental stages [22], indicating that *RpL32*, *RpS17*, and *ACT* are the most stable genes in different life stages of the mosquito. Another study conducted a meta-analysis of human gene matrices. It revealed that several reference candidate genes, such as *OAZ1* and *RpS20*, are more stable than the traditionally used HK genes *ACTB* and *GAPDH* [15].

An RNA-seq dataset can be used to obtain a reference candidate gene [15]. Previous work has already demonstrated the potential of transcriptomic data in selecting reference genes [13]. However, different criteria are used for selecting genes with high expression and low variation in transcriptome libraries and other types of high-throughput data [8, 36, 37].

Eisenberg and Levanon [9, 13] developed a methodology using Reads Per Kilobase Million (RPKM) and the size of introns, exons, and coding sequences to determine whether there was a difference between HK and non-HK genes to normalize microarray datasets and RT-qPCR experiments. This methodology was subsequently modified by Yajuan Li et al. for systematic identification and validation of reference genes in the scallop transcriptome [13]. They used a stepwise criteria system based on transcripts per million (TPM) to compare gene expression between RNA-Seq libraries [9, 13]. The goal was to search for reference genes that RT-qPCR could easily amplify, irrespective of whether they were housekeeping genes. One of the advantages of using TPM instead of RPKM to compare gene expression between libraries is a direct comparison of gene expression between libraries, eliminating the substantial inconsistencies that RPKM could cause among samples [13].

Choosing an adequate reference gene for a determined biological RNA-seq dataset is essential for better understanding RT-qPCR gene expression. The lack of a consensus on the criteria for selecting reference genes, the different types of transcriptome data, and the use of previously established HK genes as endogenous controls can lead to a misinterpretation of gene expression in a particular sample. Therefore, GSV software was developed to identify, in a set of RNA-seq libraries, the most stable (reference candidate) genes and the most variable (validation candidate) genes between treatments, assuring they have enough expression to be used in RT-qPCR. GSV speeds up the analysis, thus reducing the chances of errors and costs of the whole process.

## Implementation

### Development of the GSV software

The GSV software was developed using the Python programming language and the Pandas [38], Numpy [39], and Tkinter [40] libraries.

The software’s algorithm follows a filtering-based methodology that uses TPM values to compare gene expression between RNA-seq transcriptome samples, adapted from Li et al. [13].

The program groups the transcriptome quantification tables (TPM values) in a data frame. Then, the established criteria were applied to remove all genes that did not meet the requirements and order the candidates. Finally, a file is returned with a table indicating which genes are the most stable and which are the most variable.

The Tkinter library was used to create a graphical interface that allows the entire process to be performed without using the command line and accepts different file formats, such as .xlsx, .txt, and .csv, making the software user-friendly.

### Identifying reference genes

**Fig. 1:**
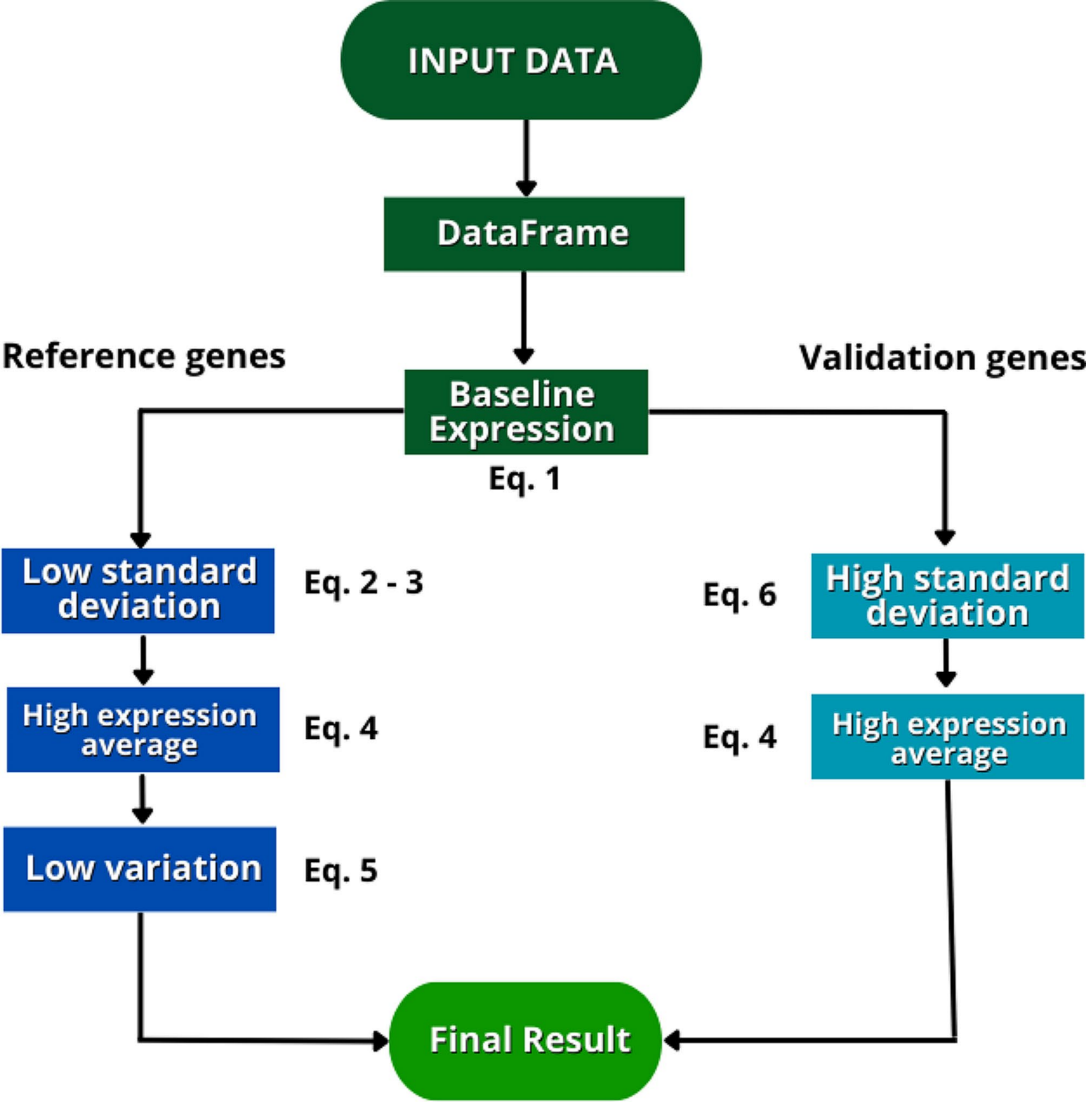
GSV software logic. The left-hand side path shows the genes with the most stable expression (reference candidate genes), and the right-hand path shows the genes with the most variable expression (validation candidate genes). Equation 1: TPM > 0; Eq. 2: SD(Log2TPM) < 1; Eq. 3: |Log2TPM - AVRG(Log2TPM)| < 2; Eq. 4: AVRG(Log2TPM) > 5; Eq. 5: CV < 0.2; Eq. 6: SD(Log2TPM) > 1. Where TPM is transcripts per million, SD is standard deviation, AVRG is average, and CV is coefficient of variation. The equations are described in the text

The criteria Yajuan Li et al. [13] provided for identifying reference genes were adapted. The genes must (I) have an expression greater than zero in all libraries analyzed (Eq. 1); (II) have low variability between libraries, represented by a standard variation smaller than one (Eq. 2); (III) not have an exceptional expression in any library, at most twice the average of log_2_ expression (Eq. 3); (IV) have a high level of expression, represented by an average of log_2_ expression above five (Eq. 4); and finally, (V) has a low coefficient of variation, which must be less than 0.2 (Eq. 5). These filters are organized in a workflow (Fig. 1).

1 $${\left({TPM}_{i}\right)}_{i=a}^{n} >0$$

2 $$\sigma \left({{log}_{2}\left({ TPM}_{i}\right)}_{i=a}^{n}\right) < 1$$

3 $$\left|{{ log}_{2}\left({ TPM}_{i}\right)}_{i=a}^{n} - \overline{{log}_{2}TPM} \right| <2$$

4 $$\overline{{log}_{2}TPM} > 5$$

5 $$\frac{\sigma \left({{log}_{2}\left({ TPM}_{i}\right)}_{i=a}^{n}\right)}{\overline{{log}_{2}TPM}} < 0.2$$

For all equations, “*TPM”* means transcripts per million, “$\sigma$” is the symbol for standard deviation, “*a”* was the first library analyzed, *“i”* was the first one and *“n”* was the last one, considering the entire transcriptome. The values at the end of all equations are the recommended standard filter values for optimal gene selection.

### Identifying validation genes

The computational identification of variable genes can aid in the experimental validation of a quantitative transcriptome. The GSV filter criteria aim to select genes that are within the detection limit of RT-qPCR and have a considerable difference between samples. The GSV applies more general filters to remove genes with low or invariable expression. The criteria suggested and used in the test analysis presented below are as follows: (I) the genes must have an expression greater than zero in all libraries analyzed (Eq. 1); (II) they must have a high variation between libraries, represented by a standard variation higher than one (Eq. 6); and (III) they must ensure a high level of expression, represented by an average of log_2_ expression above five (Eq. 4). This stage of the software is an adaptation of the methods of Yajuan Li et al. [13]. These filters are organized in a workflow (Fig. 1).

6 $$\sigma \left({{log}_{2}\left({ TPM}_{i}\right)}_{i=a}^{n}\right) > 1$$

### Tuning cutoff values

Despite our recommendation of using the standard cutoff values, the user can modify them through the software interface to loosen the search for more efficiency based on the TPM values obtained in the transcriptome.

## Results and discussion

### Input and output

**Fig. 2:**
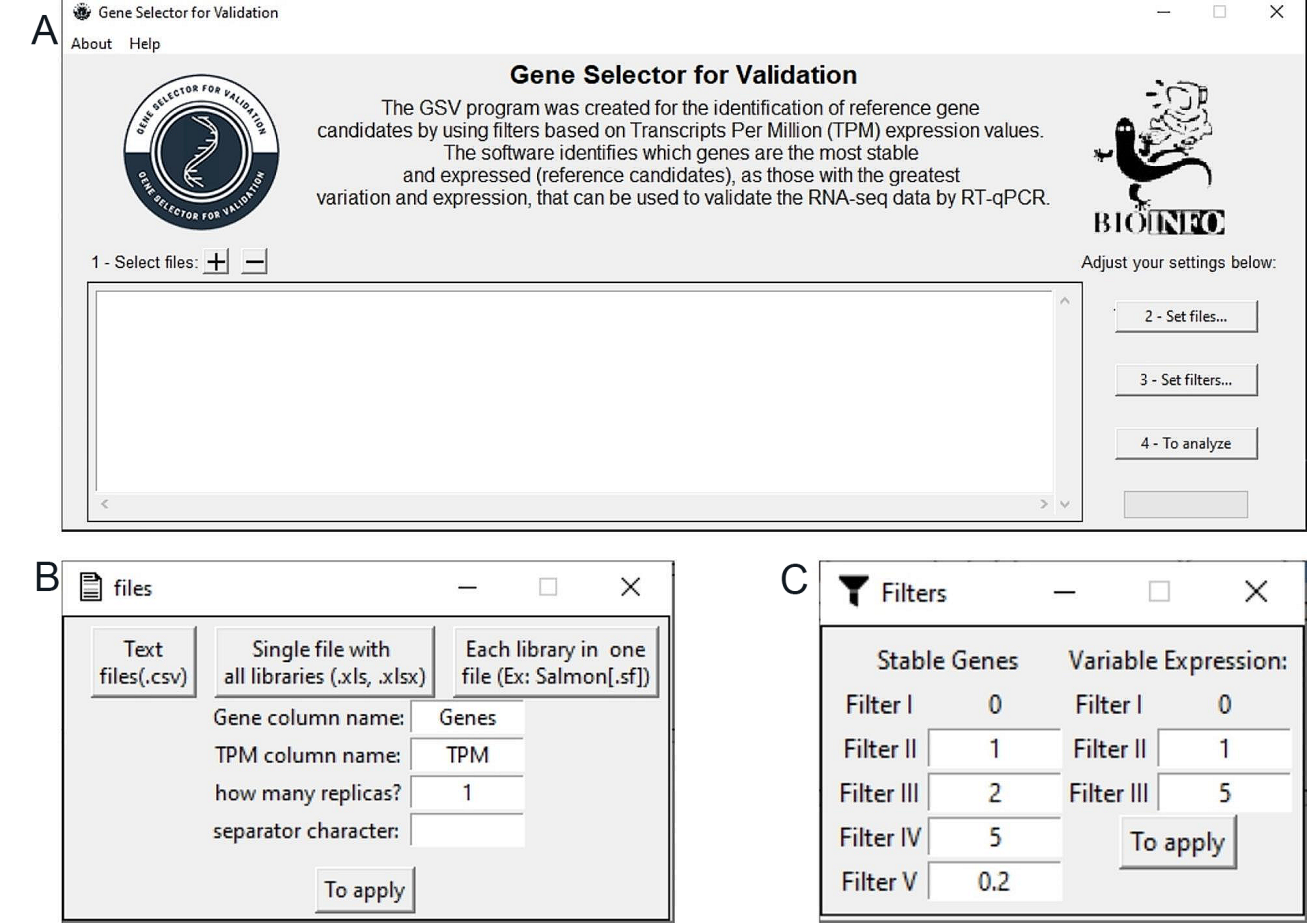
Graphical interface windows of the Gene Selector for Validation (GSV) software. **(A)** Main program window with 1-2-3-4 steps. (1) Input data, (2) Set file details, (3) Set filter cutoffs, (4) Analysis. **(B)** The set file detail window. The user must choose a file extension and add additional information that fits the input data. **(C)** Set filter cutoff window. The user can modify the cutoffs; however, it is recommended to maintain the default values

GSV’s graphical interface was created using the Tkinter library to be as intuitive as possible for the user. On its initial screen (Fig. 2A), the user can set up the input files, configure their details (Fig. 2B), tune the filters by changing the equation standard cutoffs (Fig. 2C), and access additional information, such as the user manual and developer information.

When selecting the plus button in the “Select file” window, the program allows the user to upload the file. The program accepts two types of input. The first option is a table (.csv, .xls, and .xlsx) with the gene names in the first column, followed by the TPM values, without replicas. The replica averages must be preprocessed to use table input in GSV. The input table must be correctly organized to allow the GSV to convert it to a data frame. The genes are expected in the rows, and the average TPM values are in the columns. The second input option uses files generated by the Salmon software (.sf), where the user needs to include the files and indicate the replicates so that GSV can average them.

The filter window will show the default values presented in Eqs. 1–6, and most of them can be changed. The filters are numbered according to their use in the GSV code for each gene selection pathway. The reference gene filters I-V are based on Eqs. 1–5. For the validation genes, filters II and III use Eqs. 6 and 4, respectively.

**Fig. 3:**
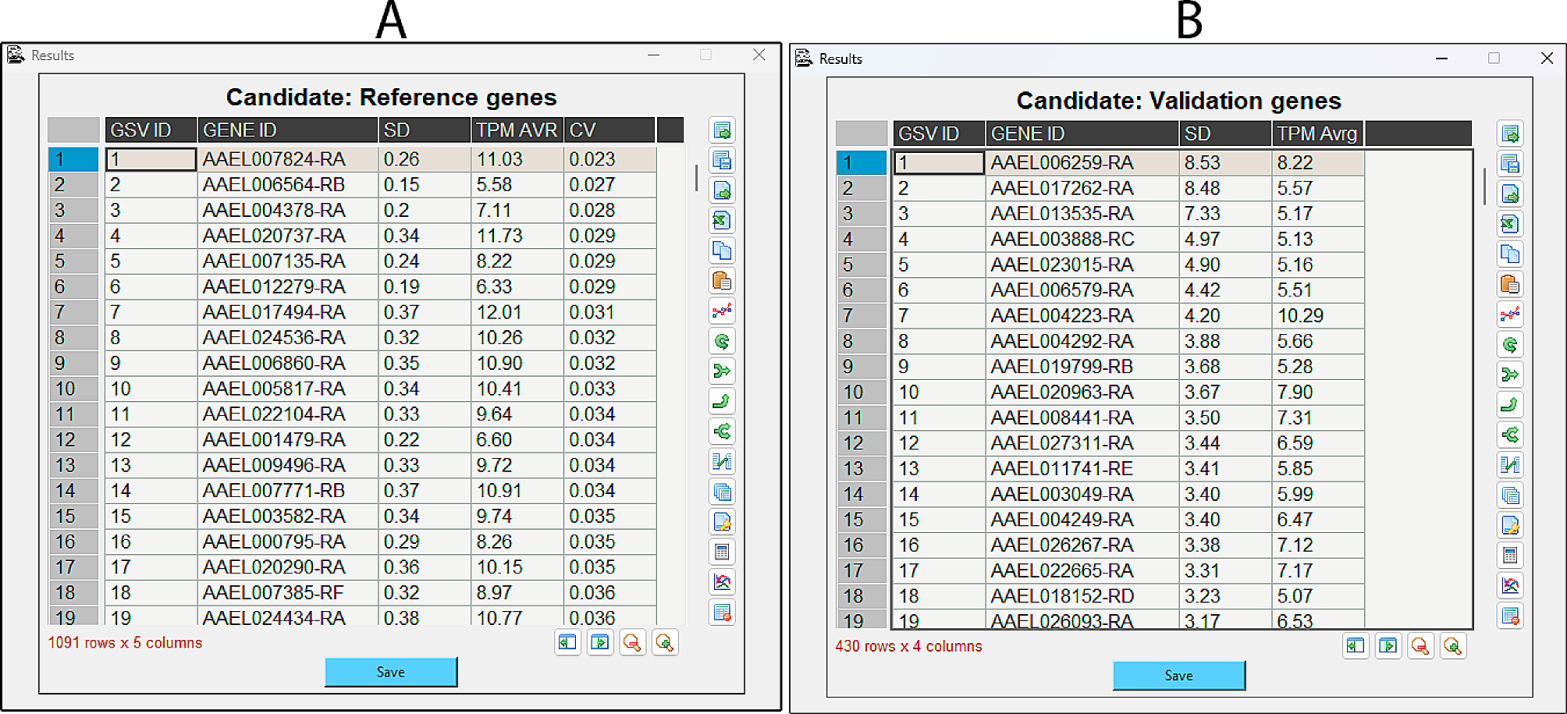
GSV-generated output results. **(A)** Results for the reference candidate genes. **(B)** Results for the validation candidate genes. The “SD” column contains the results of Eq. 2 (reference genes) or 6 (validation genes). The column “TPM Avrg” represents the log_2_TPM average and includes the results of Eq. 4. The “CV” column contains the results of Eq. 5

Upon completion of the analysis, the program displayed two new windows for presenting the results, ordering the suggestions for the reference and validation genes (Fig. 3). These tables can be saved in formats txt, xls, and xlsx). They will contain the gene identification and their respective numeric values supporting the selection.

### Identification of stable and variable genes in completely synthetic datasets

A controlled synthetic dataset mimicking an RNA-seq quantification table was created with 50,000 genes and six libraries. The quantification values were randomly generated between 0.01 and 1,000. The genes were selected based on reference candidate gene cutoffs until the completion of three groups following a normal distribution. The first group had 49,500 genes that will fail at least in the coefficient of variation filter (Eq. 5). The second group had 400 genes that would pass all GSV filters, and the third group had 100 genes that would fail only in the expression level filter (Eq. 4). No restrictions were applied to the validation candidate genes. We compared GSV results with software that stated micro-array data processing ability (OLIVER and NormFinder). The synthetic dataset had no gene with zero TPM value despite being very common in RNA-seq data. This choice avoided manual pre-processing of the synthetic dataset, as OLIVER does not accept any gene with zero expression. The NormFinder running was aborted after one hour without any result. OLIVER ran very fast, resulting in reference candidate genes similar to GSV in the top 50 genes (Table 1 and S1). OLIVER’s result does not remove stable genes with low RNA-seq expression, so one gene with a low average TPM was in the top 50 list of the “avgexpratio_avgcv” OLIVER method. Considering the three OLIVER standard output methods, one to three low-expression genes were in the top 100 and 32 to 67 in the top 400 (Table S1). The OLIVER CV method result included 124 variable genes (based on GSV cutoffs) in the top 400 list of reference candidate genes (Table S1).

A second synthetic dataset was created, changing only the third group of stable genes with low RNA-seq expression. We created this group with a CV lower than 0.05. This situation stressed the GSV and OLIVER differences. The GSV result didn’t change, but almost all low-expression genes were in the OLIVER top 100 more stable genes in all standard calculated methods (Table 1 and S2). A gene with low RNA-seq expression probably will not amplify in the validation RT-qPCR and should be removed from the list of reference candidate genes. All these differences are not defects but a consequence of the software objective. OLIVER was not planned to deal with RNA-seq data and did not have filters, so it consequently classified all genes from the input dataset. The validation candidate gene list was created only by GSV (Table 2), as other software does not have this function.

**Table 1:** The top ten reference candidate genes indicated by GSV and OLIVER in the synthetic datasets 1 and 2. The rank order (GSV ID) of the genes (ID) was based on the coefficient of variation (CV). The OLIVER gene orders were based on the three standard methods of calculated CV, methods 10 and 14 [28]. The low-expression genes filtered out by GSV are in italics

				**Synthetic Dataset 1**				
**GSV**			**OLIVER**					
**GSV ID**	**ID**	**CV**	**ResultFile**	**CV**	**ResultFile**	**Method 10 (geomean_expratio_cv)**	**ResultFile**	**Method 14 (avgexpratio_avgcv)**
1	gene377	0.0113379	gene377	0.0773104	gene377	0.3356463	gene377	0.7876236
2	gene290	0.0129895	gene290	0.0845927	gene290	0.3438141	gene290	0.8641141
3	gene222	0.0202799	gene222	0.1297098	gene325	0.4320022	gene155	0.9153812
4	gene155	0.0203968	gene155	0.1340130	gene155	0.4332223	gene286	0.9249259
5	gene325	0.0209477	gene325	0.1345984	gene286	0.4476606	gene222	0.9330298
6	gene286	0.0215052	gene286	0.1434339	gene222	0.4485550	gene273	0.9664869
7	gene133	0.0271640	gene133	0.1744992	gene133	0.5041332	gene342	0.9692297
8	gene342	0.0271994	gene342	0.1749626	gene340	0.5065868	gene325	0.9852902
9	gene273	0.0278454	gene273	0.1775537	gene480	0.5066982	gene340	0.9861197
10	gene480	0.0282302	gene378	0.1786234	gene378	0.5124461	gene402	0.9901295
				**Synthetic Dataset 2**				
**GSV**			**OLIVER**					
**GSV ID**	**ID**	**CV**	**ResultFile**	**CV**	**ResultFile**	**Method 10 (geomean_expratio_cv)**	**ResultFile**	**Method 14 (avgexpratio_avgcv)**
1	gene377	0.0113379	*gene49*	*0.0720397*	*gene49*	*0.31417451*	gene290	0.7939459
2	gene290	0.0129895	gene377	0.0773104	gene377	0.33298232	*gene55*	*0.7962386*
3	gene222	0.0202799	gene290	0.0845927	gene290	0.34160419	*gene49*	*0.8057665*
4	gene155	0.0203968	*gene69*	*0.0922727*	*gene50*	*0.36414906*	gene377	0.8169944
5	gene325	0.0209477	*gene50*	*0.0939697*	*gene69*	*0.36678234*	gene286	0.8496790
6	gene286	0.0215052	*gene88*	*0.0945400*	*gene34*	*0.37005926*	*gene62*	*0.8551309*
7	gene133	0.0271640	*gene34*	*0.0979758*	*gene81*	*0.37152753*	*gene43*	*0.8677893*
8	gene342	0.0271994	*gene81*	*0.0992733*	*gene88*	*0.37557419*	*gene58*	*0.8712006*
9	gene273	0.0278454	*gene5*	*0.1006912*	*gene65*	*0.3853843*	*gene5*	*0.8720661*
10	gene480	0.0282302	*gene39*	*0.1030613*	*gene39*	*0.38661365*	*gene65*	*0.8764844*

**Table 2:** The top ten validation candidate genes indicated by GSV in the synthetic datasets 1 and 2. The rank order (GSV ID) of the genes (ID) was based on the standard deviation (SD). TPM AVRG means the average of the Log2TPM

GSV ID	ID	SD	TPM AVRG
1	gene49049	6.50771218	6.4819876
2	gene20524	6.50356912	6.5199902
3	gene3942	6.47505851	6.1725836
4	gene46239	6.42478353	6.3101188
5	gene10627	6.40934803	6.2874486
6	gene20827	6.40510379	6.3650915
7	gene43908	6.36618735	6.2642036
8	gene34484	6.36455739	6.0959488
9	gene19851	6.33686152	6.2016036
10	gene36982	6.11444776	5.6513914

### Identification of stable and variable genes in the *Aedes aegypti* transcriptome

A previous transcriptome published by our group (bioproject PRJNA659517 [41]) was used to test the GSV algorithm. The RNA-seq data were obtained from the development time course of the adult *Ae. aegypti* mosquitoes. This mosquito is the major vector of arboviruses, such as Dengue, Zika, and Chikungunya, in tropical regions [41–43]. The samples were collected from males and females, heads and bodies at 2, 12, 24, 48, and 96 h after the emergence (the transition from pupae to adults). The details are provided in the supplementary material. This dataset set was selected to test this software because it has many different conditions and could challenge the reference genes commonly used for *Ae. aegypti* mosquitoes, including RpS7, RpL32, Actin, and GAPDH [19, 20, 22]. Additionally, ribosomal genes were differentially expressed under some conditions [41]. Therefore, using GSV could lead to the discovery of new reference genes for RT-qPCR validation of differentially expressed genes in these complex data.

**Fig. 4:**
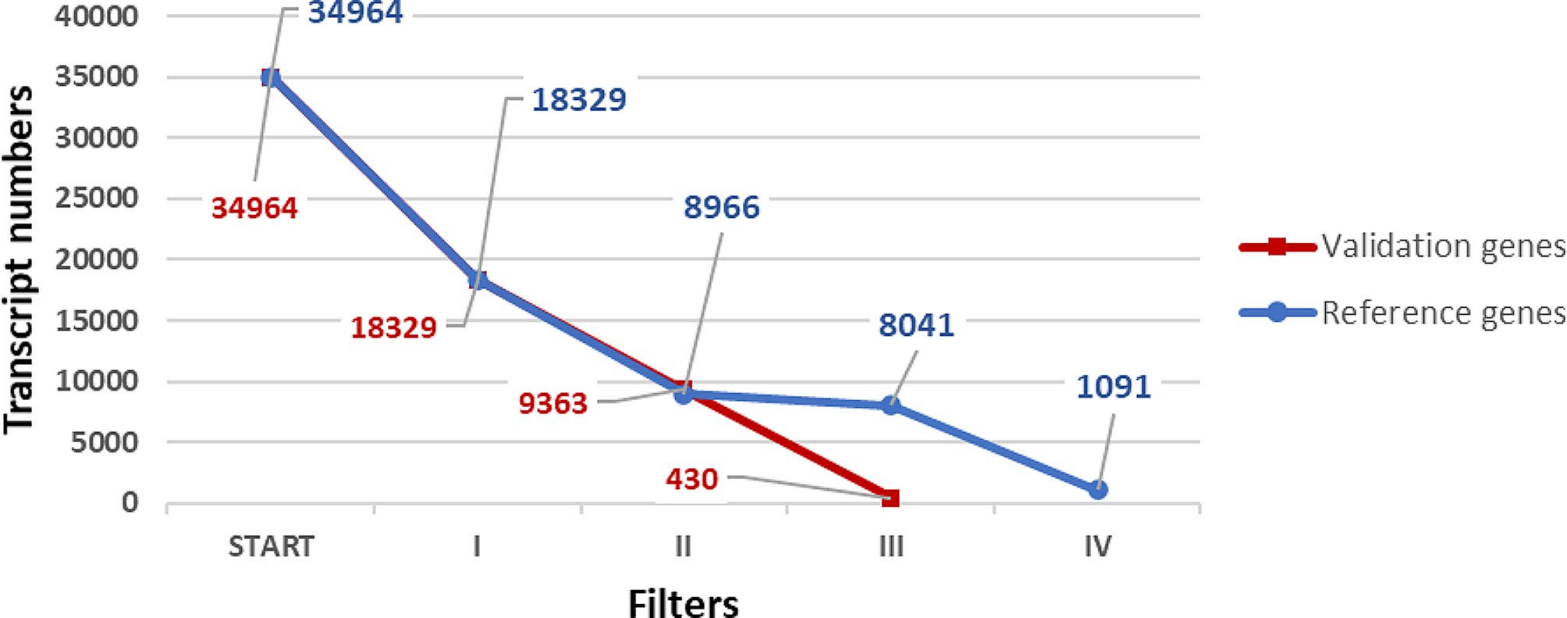
Decrease in transcript number during each software process selection stage (filters). The red line represents the selection of validation candidate genes, and the blue line represents the selection of reference candidate genes. The colored numbers represent the candidates’ numbers after each filter step. The complete final results are shown in Tables S1 and S2

An input table (.xlsx) with the gene identifiers and their respective average TPM for each development time was used for the analysis. The default cutoffs for the filters were used in the program. The predicted *Ae. aegypti* proteins in the genome (version 5.1) have 34,964 transcripts. After Filter I (Eqs. 1), 18,329 transcripts showed expression values above zero. The filters that followed the reference gene selection path included filters II (Eq. 2), III (Eq. 3), IV (Eq. 4), and V (Eq. 5) with 9,663, 8,966, 8,041 and 1,091 genes, respectively, after each filter. The validation gene selection path had only three filters, and it is relevant to remember that the last two were different from the reference path. For Filters II (Eq. 6) and III (Eqs. 4), 9,363 and 430 genes were selected in the validation path. Overall, there were 430 validation candidate genes and 1,091 reference candidate genes (Fig. 4).

The analysis of this RNA-seq dataset took only a few seconds, and it was possible to identify the genes with the most stable and most variable expression. The top ten reference candidate genes (Table 3 and S3) included five ribosomal genes (*RpS29* - AAEL007824-RA, *RpS21* - AAEL020737-RA, *RpL19* - AAEL024536-RA, *RpS28* - AAEL006860-RA, and *RpL26* - AAEL005817-RA), and other four were mainly related to protein biosynthesis (*MSP* - AAEL006564-RB, *eiF-1 A* - AAEL004378-RA, *prdx5* - AAEL007135-RA, and *eiF3j* - AAEL012279-RA). Only one gene (AAEL017494-RA) was not annotated in version 5.1 of the Vector Base *Ae. aegypti* data.

**Table 3:** The top ten reference candidate genes indicated by GSV in the PRJNA659517 transcriptome. The rank order (GSV ID) of the VectorBase genes (ID) was based on the coefficient of variation (CV)

GSV ID	ID	Gene annotation	CV
1	AAEL007824-RA	40 S Ribosomal protein S29 (*RpS29*)	0.0234100
2	AAEL006564-RB	Mitochondrial splicing protein (*MSP*)	0.0274915
3	AAEL004378-RA	Eukaryotic translation factor 1 A (*eiF-1 A*)	0.0284984
4	AAEL020737-RA	40 S Ribosomal protein S21 (*RpS21*)	0.0287212
5	AAEL007135-RA	Peroxiredoxin 5 (*prdx5*)	0.0293174
6	AAEL012279-RA	Eukaryotic translation factor 3, subunit J (*eiF3j*)	0.0293384
7	AAEL017494-RA	unidentified gene	0.0305761
8	AAEL024536-RA	Ribosomal protein L19 (*RpL19*)	0.0316085
9	AAEL006860-RA	40 S Ribosomal protein S28 (*RpS28*)	0.0324845
10	AAEL005817-RA	Ribosomal protein L26 (*RpL26*)	0.0325360

The expression of ribosomal genes, including ribosome subunits, increased over time and is sex-related, revealing a relevant positive regulation [41, 44]; consequently, these genes were discarded to avoid problems. Thus, of the ten most stable genes found by the software, *eiF1A*, *eiF3j*, *prdx5*, and *MSP* were chosen as reference candidate genes. Three other genes recommended in the literature (*RpS7* - AAEL004175-RA, *RpL32* - AAEL003396-RA, and *ACT* - AAEL011197-RC and AAEL011197-RD) [19, 20, 22] were selected for comparison.

**Fig. 5:**
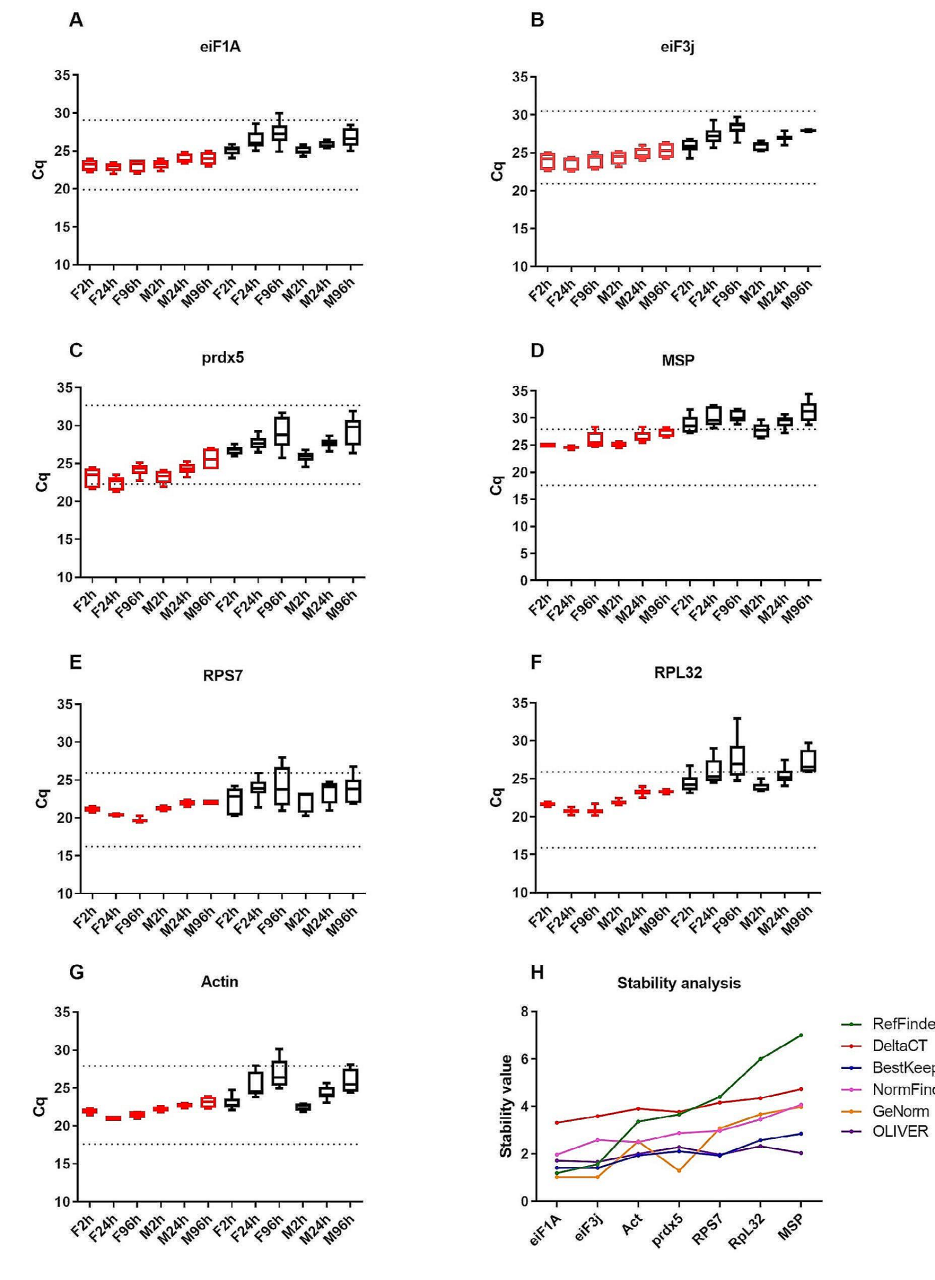
Cq variation of the four reference candidate genes (A-D) and the three recommended genes in the literature (E-G) during the postemergence phase of *Aedes aegypti* mosquitoes. **(A)** Eukaryotic translation factor 1 A – eiF-1 A; **(B)** Eukaryotic translation factor 3, subunit J – eiF3j; **(C)** Peroxiredoxin 5 – prdx5; **(D)** Mitochondrial splicing protein – MSP; **(E)** 40 S ribosomal protein S7 - *RpS7*; **(F)** L32 ribosomal protein – *RpL32*; **(G)** Actin; **(H)** stability analysis with OLIVER, RefFinder, DeltaCT, BestKeeper, NormFinder, and GeNorm. The body (red box-whisker plot) and head (black box-whisker plot) of male (M time points) and female (F time points) mosquitoes were analyzed. The upper and lower whiskers of the bars represent the highest and lowest observations, respectively. The line inside the bars represents the median. The dotted lines represent the Cq range in the standard curve. The raw Cq values are in Table S5

After selecting the reference candidate genes, RT-qPCR was performed to verify their stability between the different sample conditions of the postemergence adult phase of the *Ae. aegypti* mosquito (Fig. 5A-G). The Cq values obtained via RT-qPCR for the various genes were subjected to statistical analysis with different software packages, such as OLIVER, RefFinder, BestKeeper, NormFinder, and GeNorm, and also using the DeltaCT method was used to determine gene stability (Fig. 5H). Among the genes traditionally used in the literature, *RpS7* (Fig. 5E) was considered stable between the biological conditions in the transcriptome. *RpL32* and *ACT* (Fig. 5F-G) showed more time-dependent variation, and the *RpL32* data more pronouncedly crossed the Cq range limit of the standard curve. Despite the *ACT* variation observed in Fig. 5G, the statistical analysis (Fig. 5H) indicated that the expression of this gene was more stable than or slightly better than that of the *RpS7* gene. Wider Cq ranges observed in *RpS7* than in *ACT* could be the reason for this difference. On the other hand, for the genes chosen from the GSV analysis, *eiF1A* and *eiF3j* (Fig. 5A-B) were the most stable, surpassing the traditional genes’ stability (Fig. 5H). According to the MIQE guidelines for RT-qPCR experiments [45], we selected two genes, *eiF1A* and *eiF3j*, as reference genes.

The top ten validation candidate genes (Table 4 and S4) included proteins with diverse biological functions, including photoreceptors (AAEL006259-RA), defense proteins (the cuticle protein AAEL017262-RA and the cecropin precursor AAEL004223-RA), cell cycle, and transcriptional regulators (Phosrestin II AAEL013535-RA, the transcription elongation factor AAEL004292-RA, and the muscle lim protein AAEL019799-RB), and protein degradation members such as polyubiquitin (AAEL003888-RC). In addition, three unidentified genes were considered validation candidates (AAEL023015-RA, AAEL006579-RA, and AAEL020963-RA). The validation candidate genes identified with GSV were not used in the previously published work because we needed to confirm gene cluster expression patterns, limiting the choices to genes inside each cluster. The genes Actin-4 (AAEL001951-RA), D7 family salivary proteins (AAEL006423-RA and AAEL026087-RA), female-specific chymotrypsin (AAEL003060-RA), and polyphenol oxidase 5 (AAEL013492-RA) were used to validate the clusters B1, B2, H1, B3, and H4 [41].

**Table 4:** The top ten validation candidate genes indicated by GSV in the PRJNA659517 transcriptome. The rank order (GSV ID) of the VectorBase genes (VB ID) was based on the standard deviation (SD)

GSV ID	VB ID	Gene annotation	SD
1	AAEL006259-RA	photoreceptors R1-R6 (GPROP2)	8.527251681
2	AAEL017262-RA	Insect cuticle protein	8.476450384
3	AAEL013535-RA	Phosrestin ii	7.326451716
4	AAEL003888-RC	Polyubiquitin	4.972236262
5	AAEL023015-RA	unidentified gene	4.89721388
6	AAEL006579-RA	unidentified gene	4.418938336
7	AAEL004223-RA	Cecropin precursor	4.204042765
8	AAEL004292-RA	Transcription elongation factor	3.880707628
9	AAEL019799-RB	Muscle lim protein	3.678006944
10	AAEL020963-RA	unidentified gene	3.669554077

One example of the importance of selecting reference genes for RT-qPCR via a case-by-case approach is the glyceraldehyde-3-phosphate dehydrogenase (GAPDH) gene. This gene was commonly used as a reference gene in the literature [15, 46]; however, it was not found in the list of reference candidate genes but in a poor position on the list of genes with the most variable expression list (Table 5 and S4). It is not a good reference for RT-qPCR for this particular set of samples. The study of Dzaki et al., 2017 [22] has already suggested that GAPDH was not a stable choice in most cases.

Notably, the *RpL32* and *ACT* genes tested via RT-qPCR were in very low positions in the GSV reference candidate gene order, with 153rd and 661st, respectively (Table 5 and S3). The RT-qPCR results of these genes showed more variation than those of genes selected by GSV; therefore, these genes, especially for *ACT*, would be a poor choice. The other genes identified by the study of Dzaki [22] were *RpL8*, *α-Tubulin*, and *GAPDH*. The first was at the 148th position, close to *RpL32* in the reference candidate gene list, while GSV did not even select the other two in this list. The *α-Tubulin* and *GAPDH* genes were included in the validation gene list (329th and 418th of 430 genes) (Table 5 and S4), corroborating the data already shown [22].

**Table 5:** Genes identified by Dzaki et al. (2017), Xi et al. (2008), and Almeida et al. (2023) [19, 20, 22] and their GSV results. The rank order (GSV ID) of the VectorBase genes (ID) was based on the coefficient of variation (CV)

GSV ID	ID	Gene name	CV
**Candidates for reference genes**			
13	AAEL009496-RA	RpS7	0.03407860
32	AAEL004175-RA	*RpS17*	0.0374068
148	AAEL000987-RA	*RpL8*	0.0523476
153	AAEL003396-RA	*RpL32*	0.0529165
661	AAEL011197-RC	*ACT*	0.0975963
692	AAEL011197-RD	*ACT*	0.1011038
**Candidates for validation genes**			
329	AAEL013229-RA	*α-Tubulin*	Not calculated
418	AAEL016984-RA	*GAPDH*	Not calculated

### Identification of stable and variable genes in a genome-resolved meta-transcriptome that used synthetic microbiota

The manuscript of Vannier et al. (2023) [47] studied the repopulation of germ-free Arabidopsis thaliana roots using a synthetic microbiota with known composition. They used rRNA depletion and deep RNA sequencing followed by read mapping against reference microbial, fungal, and plant genomes. The authors did not validate the bacterial gene expression using RT-qPCR; they validated the identified bacterial taxonomic distribution within the meta-transcriptome with a DNA qPCR for the known bacterial species.

Notwithstanding, the Vannier et al. paper indicates the expression of some bacterial single-copy genes known as housekeeping (HK) based on previous meta-transcriptomics meta-analysis [48]. The Vanier et al. meta-transcriptomic expression table has over 90,000 genes and two conditions, and it was processed with GSV to check the stability of those genes considered HK. The GSV result indicated that (i) some genes with zero TPM in one or both conditions were left at the HK gene analysis and had the Log2FoldChange calculated in Vannier et al. manuscript. (ii) The HK gene better positioned at the GSV result (559|3866) was at the 343rd position (Table 6). Meta-transcriptomic validation using RT-qPCR would be possible based on reference candidate genes that are more stable than the HK genes (Table 6).

**Table 6:** GSV analysis of the Vannier et al. (2023) meta-transcriptome [47]. The top ten reference candidate genes indicated by GSV were listed, indicating the rank order (GSV ID) of the genes (ID) based on the coefficient of variation (CV). The GSV top ten housekeeping genes listed by Vannier et al. (2023) in the meta-transcriptomics were included. Their rank order (GSV ID) organizes them based on the coefficient of variation (CV). AVRG TPM: average of the Log2TPM (Eq. 4)

GSV ID	ID	Gene name	CV	AVRG TPM
**Candidates for reference genes**				
1	322|1301	-	1.209E-05	8.4080
2	559|3566	-	1.366E-05	10.4436
3	559|904	-	1.465E-05	10.3520
4	670|2540	-	4.358E-05	7.81075
5	569|4115	-	4.551E-05	6.1970
6	149|979	-	4.553E-05	8.2784
7	559|2310	-	4.948E-05	8.3695
8	670|2349	-	6.833E-05	7.4814
9	154|4086	-	6.851E-05	10.2664
10	61|1606	-	9.688E-05	6.4324
**Housekeeping genes**				
343	559|3866	rpoC	0.002847	11.5358
344	154|680	dnaG	0.002904	9.86601
470	322|1497	adk	10.19230	0.003822
836	154|836	secA	9.84736	0.006997
1063	123D2|886	adk	11.19050	0.008808
1302	181|1157	rpoB	13.00365	0.01091
1664	149|905	dnaG	7.18954	0.01384
1725	1277|4109	rpoC	10.9993	0.014280
1790	559|2137	rho	10.8979	0.014936
1945	322|822	rho	10.4543	0.01620

## Conclusion

Gene Selector for Validation (GSV) software effectively identified reference candidate genes with stable and measurable expression in RNA-seq datasets. The synthetic datasets analyzed showed that the GSV result was clear of low- and variable-expression genes. The identified mosquito RNA-seq reference candidate genes were confirmed via RT-qPCR, and the Cq values were analyzed using statistical analysis. The genes eiF1A and eiF3j were identified as those with the most stable expression in our dataset. The genes *RpS7, RpL8, RpL32*, and *ACT* suggested in the literature [19, 20, 22] were retrieved by GSV as worse reference options and confirmed by RT-qPCR. The use of GSV prevents researchers from relying solely on the reference genes of previous studies, which may not align with their specific experimental conditions. The meta-transcriptome processed was gene-resolved, allowing the GSV identification of genes with a stable and likely measurable expression by RT-qPCR. This allowed reference candidate gene identification, which is suitable for a particular and complex dataset. Validation candidate genes with variable and measurable RT-qPCR expression were identified by GSV in all analyses and complemented the reference candidate genes identification. The GSV is easy to use due to its graphical interface and fast response time. It has cost-saving benefits, avoiding using inadequate reference and validation genes and redoing the RNA-seq validation RT-qPCRs. The software is available for free, fostering RNA-seq analysis by identifying reliable and RT-qPCR quantifiable reference and validation candidate genes.

### Electronic supplementary material

Below is the link to the electronic supplementary material.

Supplementary Material 1

## Data Availability

The authors declare that the datasets used in this paper are available at the NCBI BioProject PRJNA659517 and in the supplementary material of Vannier et al. [47]. The synthetic datasets are available on the software home page.

## Declarations

### Code availability

Project name: Gene Selector for Validation (GSV)

Project home page: https://github.com/rdmesquita/GSV

Operating system(s): tested on Windows 10/11

Programming language: Python 3.9.13

Other requirements: N/A

License: GNU 3.0

Any restrictions to use by non-academics: none.

## Abbreviations

PCR: Polymerase chain reaction
RT: qPCR-Reverse transcriptase quantitative PCR
HTS: High throughput sequencing
RNA: Ribonucleic acid
RNA: Seq-Ribonucleic acid sequencing
GAPDH: Glyceraldehyde 3-phosphate dehydrogenase
RpS7: 40 S ribosomal protein S7
Cq: Quantitation cycle
RpL32: Ribosomal protein L32
RpS17: Ribosomal protein S17
ACT: Actin
HK: Housekeeping genes
RPKM: Reads per kilobase million
TPM: Transcripts per million
CV: Coefficient of variation
MSP: Merozoite surface protein
