# Supplementary material for: RNA-seq validation: software for selection of reference and variable candidate genes for RT-qPCR
Source: BMC Genomics. 2024 Jul 16;25:697. doi: 10.1186/s12864-024-10511-y (PMC11251314; doi:10.1186/s12864-024-10511-y)
Supplement: Supplementary file 1 — Supplementary Material 1 [file 12864_2024_10511_MOESM1_ESM.docx]

**RNA-seq validation: Software for selection of reference and variable candidate genes for RT-qPCR**

**Supplementary material**

[Table S1: Reference candidate genes top 400 list from GSV and OLIVER for the synthetic dataset 1. The rank order (GSV ID) of the genes (ID) was based on the coefficient of variation (CV). TPM avrg: TPM average. The OLIVER orders was based on CV and methods 10 and 14 calculations in the OLIVER original paper. The low-expression genes filtered out by GSV are in red. The variable genes filtered out by GSV are in orange. 2](#_pknnrtb87m2c)

[Table S2: Reference candidate genes top 400 list from GSV and OLIVER for the synthetic dataset 2. The rank order (GSV ID) of the genes (ID) was based on the coefficient of variation (CV). TPM avrg: TPM average. The OLIVER orders was based on CV and methods 10 and 14 calculations in the OLIVER original paper. The low-expression genes filtered out by GSV are in red. The variable genes filtered out by GSV are in orange. 15](#_bhqwtzj8sjuv)

[Table S3: Reference candidate genes full list for the PRJNA659517 transcriptome. The rank order (GSV ID) of the VectorBase genes (ID) was based on the coefficient of variation (CV). TPM avrg: TPM average. 27](#_mkjogvimqldq)

[Table S4: Validation candidate genes full list for the PRJNA659517 transcriptome. The rank order (GSV ID) of the VectorBase genes (ID) was based on the standard deviation (SD). TPM avrg: TPM average. 55](#_99waqlv1psue)

[Table S5: Raw data used to create Figure 5. 65](#_2co52rq2rlhy)

[Table S6: List of primers and their characteristics. GSV ID: GSV rank order. ID: VectorBase code. 68](#_fjwzxssonv2a)

[Supplementary methodology: RNA extraction and quantitative PCR (qPCR) 70](#_u29uzq5jtus4)

##

## Table S1: Reference candidate genes top 400 list from GSV and OLIVER for the synthetic dataset 1. The rank order (GSV ID) of the genes (ID) was based on the coefficient of variation (CV). TPM avrg: TPM average. The OLIVER orders was based on CV and methods 10 and 14 calculations in the OLIVER original paper. The low-expression genes filtered out by GSV are in red. The variable genes filtered out by GSV are in orange.

| **GSV** | | | | | **OLIVER** | | | | | |
| --- | --- | --- | --- | --- | --- | --- | --- | --- | --- | --- |
| **GSV ID** | **ID** | **SD** | **TPM AVRG** | **CV** | **ResultFile** | **OLIVER cv** | **ResultFile** | **geomean expratio cv OLIVER method 10** | **ResultFile** | **avgexpratio avgcv OLIVER method 14** |
| 1 | gene377 | 0.110245 | 9.723479 | 0.011338 | gene377 | 0.077310402 | gene377 | 0.335646304 | gene377 | 0.787623642 |
| 2 | gene290 | 0.121321 | 9.339871 | 0.01299 | gene290 | 0.084592793 | gene290 | 0.343814168 | gene290 | 0.864114152 |
| 3 | gene222 | 0.197401 | 9.733793 | 0.02028 | gene222 | 0.129709827 | gene325 | 0.432002219 | gene155 | 0.91538124 |
| 4 | gene155 | 0.194373 | 9.529592 | 0.020397 | gene155 | 0.134013082 | gene155 | 0.433222302 | gene286 | 0.924925965 |
| 5 | gene325 | 0.198272 | 9.465065 | 0.020948 | gene325 | 0.134598401 | gene286 | 0.4476606 | gene222 | 0.933029894 |
| 6 | gene286 | 0.203005 | 9.439783 | 0.021505 | gene286 | 0.143433988 | gene222 | 0.448555071 | gene273 | 0.966486992 |
| 7 | gene133 | 0.251593 | 9.261978 | 0.027164 | gene133 | 0.174499233 | gene133 | 0.504133227 | gene342 | 0.969229786 |
| 8 | gene342 | 0.26335 | 9.682192 | 0.027199 | gene342 | 0.174962688 | gene340 | 0.506586854 | gene325 | 0.985290268 |
| 9 | gene273 | 0.269506 | 9.678665 | 0.027845 | gene273 | 0.177553781 | gene480 | 0.506698224 | gene340 | 0.986119772 |
| 10 | gene480 | 0.272768 | 9.662264 | 0.02823 | gene378 | 0.17862346 | gene378 | 0.512446133 | gene402 | 0.990129567 |
| 11 | gene340 | 0.265637 | 9.334172 | 0.028458 | gene340 | 0.182762622 | gene273 | 0.518897768 | gene360 | 1.000268906 |
| 12 | gene378 | 0.281031 | 9.591588 | 0.0293 | gene453 | 0.183176518 | gene453 | 0.523816339 | gene480 | 1.020982572 |
| 13 | gene409 | 0.276717 | 9.34019 | 0.029626 | gene438 | 0.186659659 | gene409 | 0.526485085 | gene300 | 1.02142674 |
| 14 | gene453 | 0.285897 | 9.6226 | 0.029711 | gene480 | 0.187862722 | gene431 | 0.531434006 | gene431 | 1.022185132 |
| 15 | gene431 | 0.283752 | 9.401054 | 0.030183 | gene402 | 0.189667715 | gene342 | 0.533253555 | gene133 | 1.037359554 |
| 16 | gene402 | 0.278525 | 9.226712 | 0.030187 | gene101 | 0.194167816 | gene402 | 0.541471139 | gene404 | 1.042510383 |
| 17 | gene300 | 0.283573 | 9.378202 | 0.030237 | gene300 | 0.195066721 | gene101 | 0.544092428 | gene101 | 1.047816098 |
| 18 | gene360 | 0.299527 | 9.536431 | 0.031409 | gene409 | 0.200642477 | gene360 | 0.548922628 | gene409 | 1.048254108 |
| 19 | gene264 | 0.306551 | 9.50114 | 0.032265 | gene404 | 0.203604703 | gene264 | 0.552939433 | gene441 | 1.05762914 |
| 20 | gene101 | 0.289697 | 8.926785 | 0.032453 | gene451 | 0.203752009 | gene438 | 0.553171808 | gene453 | 1.058513931 |
| 21 | gene438 | 0.316337 | 9.679207 | 0.032682 | gene360 | 0.205182587 | gene300 | 0.562802901 | gene264 | 1.061710704 |
| 22 | gene451 | 0.308391 | 9.266564 | 0.03328 | gene441 | 0.210116719 | gene451 | 0.568228318 | gene411 | 1.063145583 |
| 23 | gene489 | 0.326176 | 9.595713 | 0.033992 | gene264 | 0.212489647 | gene405 | 0.56961937 | gene482 | 1.06704628 |
| 24 | gene404 | 0.292991 | 8.605131 | 0.034048 | gene405 | 0.213227994 | gene237 | 0.570800855 | gene438 | 1.067602187 |
| 25 | gene405 | 0.337236 | 9.51922 | 0.035427 | gene489 | 0.214790365 | gene441 | 0.577744629 | gene237 | 1.068148683 |
| 26 | gene329 | 0.339012 | 9.519928 | 0.035611 | gene431 | 0.217532777 | gene404 | 0.579610884 | gene378 | 1.07253691 |
| 27 | gene441 | 0.346043 | 9.704271 | 0.035659 | gene380 | 0.219599644 | gene380 | 0.579853907 | gene285 | 1.073643226 |
| 28 | gene237 | 0.336744 | 9.191205 | 0.036638 | gene482 | 0.22357479 | gene489 | 0.579864548 | gene489 | 1.075803272 |
| 29 | gene380 | 0.351458 | 9.548838 | 0.036806 | gene237 | 0.226844427 | gene482 | 0.587062201 | gene451 | 1.081282578 |
| 30 | gene482 | 0.339798 | 8.879414 | 0.038268 | gene329 | 0.227254557 | gene223 | 0.599290193 | gene368 | 1.086998947 |
| 31 | gene285 | 0.360836 | 9.367363 | 0.038521 | gene219 | 0.240238004 | gene368 | 0.604581429 | gene319 | 1.096350178 |
| 32 | gene223 | 0.368917 | 9.423726 | 0.039148 | gene140 | 0.241179276 | gene491 | 0.610928298 | gene383 | 1.102155787 |
| 33 | gene411 | 0.371132 | 9.345639 | 0.039712 | gene370 | 0.244523868 | gene285 | 0.611130809 | gene462 | 1.106515969 |
| 34 | gene319 | 0.386251 | 9.432352 | 0.04095 | gene411 | 0.244903673 | gene329 | 0.612142387 | gene490 | 1.110731426 |
| 35 | gene370 | 0.370306 | 8.861963 | 0.041786 | gene142 | 0.246940105 | gene370 | 0.614374255 | gene491 | 1.116142637 |
| 36 | gene383 | 0.394944 | 9.429722 | 0.041883 | gene474 | 0.247607623 | gene363 | 0.616453281 | gene282 | 1.117581297 |
| 37 | gene490 | 0.390639 | 9.284677 | 0.042073 | gene223 | 0.251949476 | gene113 | 0.617492676 | gene329 | 1.12172684 |
| 38 | gene282 | 0.394126 | 9.349026 | 0.042157 | gene285 | 0.253396557 | gene219 | 0.618500078 | gene380 | 1.125761642 |
| 39 | gene363 | 0.394371 | 9.295877 | 0.042424 | gene121 | 0.25500676 | gene462 | 0.619438221 | gene474 | 1.126096398 |
| 40 | gene140 | 0.38969 | 9.024626 | 0.043181 | gene368 | 0.255031909 | gene140 | 0.624041149 | gene405 | 1.127193993 |
| 41 | gene491 | 0.39045 | 9.018882 | 0.043292 | gene113 | 0.255693783 | gene474 | 0.627602788 | gene370 | 1.131617982 |
| 42 | gene463 | 0.387535 | 8.929904 | 0.043397 | gene363 | 0.256310235 | gene411 | 0.629980202 | gene363 | 1.137514975 |
| 43 | gene381 | 0.405554 | 9.317427 | 0.043526 | gene462 | 0.261167595 | gene142 | 0.635711847 | gene130 | 1.13845273 |
| 44 | gene462 | 0.406756 | 9.31912 | 0.043647 | gene491 | 0.261996981 | gene395 | 0.635781776 | gene223 | 1.142561925 |
| 45 | gene113 | 0.411164 | 9.377169 | 0.043847 | gene319 | 0.263124579 | gene319 | 0.637724017 | gene463 | 1.146537294 |
| 46 | gene368 | 0.385256 | 8.713823 | 0.044212 | gene490 | 0.263702457 | gene121 | 0.638070301 | gene112 | 1.152592864 |
| 47 | gene464 | 0.409296 | 9.234585 | 0.044322 | gene464 | 0.27297408 | gene383 | 0.64001888 | gene115 | 1.156434071 |
| 48 | gene142 | 0.426945 | 9.629661 | 0.044336 | gene160 | 0.274157567 | gene282 | 0.642352268 | gene6 | 1.15688167 |
| 49 | gene121 | 0.405707 | 9.072561 | 0.044718 | gene383 | 0.275312536 | gene490 | 0.643591339 | gene265 | 1.15747372 |
| 50 | gene219 | 0.41713 | 9.298939 | 0.044858 | gene261 | 0.276680703 | gene381 | 0.646089091 | gene298 | 1.160187499 |
| 51 | gene474 | 0.419279 | 9.281502 | 0.045174 | gene381 | 0.277997578 | gene464 | 0.646172135 | gene371 | 1.160889115 |
| 52 | gene160 | 0.442174 | 9.525586 | 0.04642 | gene282 | 0.279197316 | gene261 | 0.647222126 | gene190 | 1.161468986 |
| 53 | gene190 | 0.413599 | 8.787055 | 0.047069 | gene395 | 0.280534718 | gene115 | 0.651317622 | gene261 | 1.162663006 |
| 54 | gene115 | 0.41967 | 8.908811 | 0.047107 | gene354 | 0.281923335 | gene348 | 0.651709897 | gene186 | 1.163179942 |
| 55 | gene395 | 0.433483 | 9.105883 | 0.047605 | gene196 | 0.283805757 | gene345 | 0.656377944 | gene348 | 1.167202453 |
| 56 | gene298 | 0.439013 | 9.205788 | 0.047689 | gene486 | 0.285318281 | gene403 | 0.661123503 | gene142 | 1.170116069 |
| 57 | gene403 | 0.45438 | 9.440651 | 0.04813 | gene115 | 0.286052037 | gene236 | 0.661208152 | gene269 | 1.170603504 |
| 58 | gene269 | 0.429285 | 8.891437 | 0.048281 | gene483 | 0.287186989 | gene130 | 0.663298988 | gene148 | 1.170937797 |
| 59 | gene316 | 0.456884 | 9.369227 | 0.048764 | gene217 | 0.2872596 | gene463 | 0.663308717 | gene395 | 1.176243201 |
| 60 | gene265 | 0.452076 | 9.265011 | 0.048794 | gene403 | 0.288509141 | gene371 | 0.666139146 | gene221 | 1.181170461 |
| 61 | gene281 | 0.450025 | 9.218313 | 0.048819 | gene81 | 0.290112818 | gene190 | 0.669499331 | gene478 | 1.182255228 |
| 62 | gene385 | 0.457144 | 9.347046 | 0.048908 | gene345 | 0.290474221 | gene217 | 0.674113021 | gene381 | 1.183516713 |
| 63 | gene112 | 0.451796 | 9.166367 | 0.049288 | gene371 | 0.292142502 | gene6 | 0.682991485 | gene312 | 1.183721916 |
| 64 | gene354 | 0.46519 | 9.415508 | 0.049407 | gene236 | 0.293767896 | gene385 | 0.683275976 | gene121 | 1.184697144 |
| 65 | gene162 | 0.45649 | 9.238509 | 0.049412 | gene105 | 0.29518513 | gene269 | 0.686082979 | gene217 | 1.187306253 |
| 66 | gene312 | 0.448839 | 9.072878 | 0.04947 | gene191 | 0.29713221 | gene312 | 0.686511104 | gene113 | 1.19125988 |
| 67 | gene198 | 0.471693 | 9.427414 | 0.050034 | gene130 | 0.302366285 | gene160 | 0.686929163 | gene219 | 1.198372776 |
| 68 | gene186 | 0.458638 | 9.146273 | 0.050145 | gene367 | 0.302772801 | gene354 | 0.688568428 | gene140 | 1.198576078 |
| 69 | gene217 | 0.415232 | 8.112677 | 0.051183 | gene190 | 0.302859683 | gene162 | 0.688640917 | gene464 | 1.200447381 |
| 70 | gene371 | 0.467681 | 9.134597 | 0.051199 | gene186 | 0.307570856 | gene186 | 0.69013199 | gene461 | 1.202904735 |
| 71 | gene154 | 0.477569 | 9.294193 | 0.051384 | gene269 | 0.307621151 | gene198 | 0.690994486 | gene355 | 1.204773631 |
| 72 | gene261 | 0.456646 | 8.866917 | 0.0515 | gene6 | 0.308185426 | gene81 | 0.692288263 | gene364 | 1.212894138 |
| 73 | gene348 | 0.455711 | 8.816046 | 0.051691 | gene348 | 0.308936084 | gene478 | 0.69424323 | gene198 | 1.217112941 |
| 74 | gene130 | 0.444603 | 8.569519 | 0.051882 | gene385 | 0.30984492 | gene265 | 0.699267536 | gene358 | 1.219130138 |
| 75 | gene196 | 0.47718 | 9.182479 | 0.051966 | gene198 | 0.310478143 | gene221 | 0.700830578 | gene224 | 1.223422517 |
| 76 | gene224 | 0.448343 | 8.603868 | 0.052109 | gene331 | 0.313008547 | gene224 | 0.701095401 | gene281 | 1.224449481 |
| 77 | gene355 | 0.493928 | 9.415029 | 0.052462 | gene463 | 0.31313479 | gene483 | 0.701700471 | gene391 | 1.224544452 |
| 78 | gene174 | 0.487837 | 9.276248 | 0.05259 | gene148 | 0.314911174 | gene112 | 0.702075335 | gene160 | 1.224758292 |
| 79 | gene345 | 0.500847 | 9.52274 | 0.052595 | gene316 | 0.315427643 | gene281 | 0.703479969 | gene403 | 1.22659649 |
| 80 | gene478 | 0.46561 | 8.70687 | 0.053476 | gene55 | 0.315570906 | gene355 | 0.70663241 | gene385 | 1.230311363 |
| 81 | gene270 | 0.458079 | 8.532964 | 0.053683 | gene112 | 0.316165245 | gene105 | 0.710360304 | gene316 | 1.231454386 |
| 82 | gene483 | 0.520701 | 9.60062 | 0.054236 | gene478 | 0.317602344 | gene188 | 0.710921432 | gene162 | 1.231574467 |
| 83 | gene461 | 0.495902 | 9.113772 | 0.054412 | gene314 | 0.320786543 | gene154 | 0.712347855 | gene270 | 1.2331165 |
| 84 | gene400 | 0.504218 | 9.246303 | 0.054532 | gene355 | 0.32129314 | gene196 | 0.714872717 | gene345 | 1.233572711 |
| 85 | gene485 | 0.515413 | 9.416684 | 0.054734 | gene281 | 0.321617667 | gene481 | 0.717120724 | gene236 | 1.233862686 |
| 86 | gene236 | 0.514288 | 9.341388 | 0.055055 | gene221 | 0.323021918 | gene298 | 0.717679548 | gene187 | 1.233981752 |
| 87 | gene199 | 0.49374 | 8.961057 | 0.055098 | gene265 | 0.323554674 | gene499 | 0.718373521 | gene234 | 1.235253523 |
| 88 | gene221 | 0.499155 | 8.961649 | 0.055699 | gene312 | 0.324997751 | gene270 | 0.719334562 | gene347 | 1.235872927 |
| 89 | gene443 | 0.502348 | 8.999475 | 0.05582 | gene162 | 0.326698638 | gene461 | 0.720382405 | gene154 | 1.236260303 |
| 90 | gene105 | 0.518364 | 9.275857 | 0.055883 | gene270 | 0.327241304 | gene148 | 0.721534067 | gene105 | 1.240504668 |
| 91 | gene327 | 0.493341 | 8.752264 | 0.056367 | gene154 | 0.327353742 | gene486 | 0.721864779 | gene174 | 1.2419762 |
| 92 | gene148 | 0.507125 | 8.939788 | 0.056727 | gene364 | 0.327898507 | gene191 | 0.724343402 | gene199 | 1.242561937 |
| 93 | gene364 | 0.52249 | 9.201874 | 0.056781 | gene298 | 0.328279 | gene316 | 0.726946731 | gene499 | 1.243285489 |
| 94 | gene126 | 0.529421 | 9.254366 | 0.057208 | gene174 | 0.328701734 | gene331 | 0.72718033 | gene196 | 1.245169976 |
| 95 | gene233 | 0.519669 | 9.06728 | 0.057313 | gene233 | 0.329080421 | gene174 | 0.727373049 | gene102 | 1.245986832 |
| 96 | gene234 | 0.534136 | 9.311019 | 0.057366 | gene386 | 0.329462533 | gene433 | 0.727806487 | gene437 | 1.246295716 |
| 97 | gene486 | 0.556614 | 9.661029 | 0.057614 | gene485 | 0.330662955 | gene400 | 0.727930703 | gene400 | 1.248762935 |
| 98 | gene205 | 0.535337 | 9.221263 | 0.058055 | gene234 | 0.332700096 | gene52 | 0.729073019 | gene486 | 1.253780847 |
| 99 | gene437 | 0.525694 | 9.026347 | 0.05824 | gene499 | 0.333326014 | gene358 | 0.73028348 | gene485 | 1.255959949 |
| 100 | gene499 | 0.537983 | 9.201778 | 0.058465 | gene400 | 0.334031235 | gene126 | 0.731567588 | gene188 | 1.256115436 |
| 101 | gene331 | 0.551006 | 9.416067 | 0.058518 | gene224 | 0.336381768 | gene367 | 0.73169915 | gene137 | 1.258499312 |
| 102 | gene358 | 0.548197 | 9.351661 | 0.05862 | gene389 | 0.336768659 | gene199 | 0.731888773 | gene433 | 1.263097138 |
| 103 | gene188 | 0.548555 | 9.320316 | 0.058856 | gene126 | 0.337296134 | gene0 | 0.733985006 | gene443 | 1.263379505 |
| 104 | gene391 | 0.538661 | 9.109576 | 0.059131 | gene317 | 0.338327845 | gene129 | 0.736243287 | gene52 | 1.264234665 |
| 105 | gene102 | 0.518276 | 8.758253 | 0.059176 | gene461 | 0.338883908 | gene317 | 0.736549189 | gene126 | 1.264869671 |
| 106 | gene317 | 0.535993 | 8.992143 | 0.059607 | gene207 | 0.340441833 | gene205 | 0.736981141 | gene226 | 1.26894029 |
| 107 | gene458 | 0.553143 | 9.231845 | 0.059917 | gene11 | 0.341609738 | gene225 | 0.737551696 | gene327 | 1.273823873 |
| 108 | gene191 | 0.572379 | 9.542553 | 0.059982 | gene129 | 0.343961624 | gene102 | 0.738370365 | gene389 | 1.276477026 |
| 109 | gene187 | 0.554849 | 9.234199 | 0.060086 | gene433 | 0.345505315 | gene364 | 0.740518942 | gene354 | 1.278723024 |
| 110 | gene129 | 0.558987 | 9.272431 | 0.060285 | gene188 | 0.345744634 | gene389 | 0.742997375 | gene129 | 1.279104606 |
| 111 | gene469 | 0.553644 | 9.122029 | 0.060693 | gene227 | 0.345761477 | gene108 | 0.744129752 | gene172 | 1.284150007 |
| 112 | gene314 | 0.565323 | 9.311413 | 0.060713 | gene358 | 0.346318906 | gene227 | 0.74586226 | gene483 | 1.284904025 |
| 113 | gene110 | 0.542654 | 8.916722 | 0.060858 | gene225 | 0.346887292 | gene386 | 0.747774507 | gene108 | 1.285636867 |
| 114 | gene481 | 0.552099 | 9.012274 | 0.061261 | gene481 | 0.34691354 | gene233 | 0.748306815 | gene287 | 1.287173967 |
| 115 | gene225 | 0.580758 | 9.422626 | 0.061634 | gene61 | 0.348164639 | gene437 | 0.750682194 | gene386 | 1.289645441 |
| 116 | gene415 | 0.553646 | 8.964641 | 0.061759 | gene430 | 0.349634043 | gene61 | 0.750742518 | gene11 | 1.290295471 |
| 117 | gene108 | 0.567888 | 9.16294 | 0.061977 | gene458 | 0.349853547 | gene234 | 0.75092069 | gene110 | 1.291491819 |
| 118 | gene367 | 0.58668 | 9.41637 | 0.062304 | gene0 | 0.35236032 | gene347 | 0.75096003 | gene225 | 1.293003657 |
| 119 | gene433 | 0.577804 | 9.271529 | 0.06232 | gene347 | 0.353374639 | gene287 | 0.751831748 | gene458 | 1.29391672 |
| 120 | gene295 | 0.568381 | 9.026776 | 0.062966 | gene229 | 0.358663975 | gene415 | 0.754885806 | gene13 | 1.294205007 |
| 121 | gene137 | 0.560309 | 8.851647 | 0.0633 | gene169 | 0.359767952 | gene117 | 0.755095161 | gene117 | 1.294845855 |
| 122 | gene172 | 0.570857 | 9.017421 | 0.063306 | gene108 | 0.360324738 | gene443 | 0.755374062 | gene430 | 1.296121632 |
| 123 | gene226 | 0.575414 | 8.984564 | 0.064045 | gene397 | 0.361941177 | gene187 | 0.756323325 | gene233 | 1.297876165 |
| 124 | gene117 | 0.590426 | 9.164041 | 0.064429 | gene399 | 0.36224913 | gene172 | 0.757188431 | gene415 | 1.297885144 |
| 125 | gene302 | 0.577137 | 8.91109 | 0.064766 | gene398 | 0.363564879 | gene226 | 0.758079651 | gene81 | 1.298603295 |
| 126 | gene220 | 0.60698 | 9.368835 | 0.064787 | gene410 | 0.364284218 | gene123 | 0.758405812 | gene25 | 1.298729542 |
| 127 | gene454 | 0.604395 | 9.325822 | 0.064809 | gene138 | 0.365320736 | gene327 | 0.763760886 | gene481 | 1.299213018 |
| 128 | gene318 | 0.587357 | 9.0585 | 0.06484 | gene437 | 0.365529991 | gene25 | 0.764023702 | gene469 | 1.300775858 |
| 129 | gene347 | 0.579235 | 8.910055 | 0.065009 | gene123 | 0.365861367 | gene391 | 0.76543767 | gene90 | 1.301444825 |
| 130 | gene139 | 0.599801 | 9.192913 | 0.065246 | gene205 | 0.365925277 | gene139 | 0.767182287 | gene139 | 1.303114748 |
| 131 | gene287 | 0.604772 | 9.228274 | 0.065535 | gene460 | 0.367089828 | gene485 | 0.767276536 | gene205 | 1.304345812 |
| 132 | gene386 | 0.613988 | 9.337544 | 0.065755 | gene220 | 0.370205106 | gene469 | 0.769578268 | gene220 | 1.304797279 |
| 133 | gene392 | 0.585524 | 8.860144 | 0.066085 | gene327 | 0.370249996 | gene454 | 0.770343225 | gene33 | 1.312134056 |
| 134 | gene422 | 0.618954 | 9.363546 | 0.066103 | gene187 | 0.371135937 | gene194 | 0.770703728 | gene211 | 1.313334776 |
| 135 | gene194 | 0.60239 | 9.040156 | 0.066635 | gene302 | 0.3719828 | gene424 | 0.771603803 | gene317 | 1.31384303 |
| 136 | gene389 | 0.598435 | 8.935964 | 0.066969 | gene454 | 0.373741718 | gene267 | 0.77343765 | gene277 | 1.314241031 |
| 137 | gene150 | 0.608347 | 9.067104 | 0.067094 | gene216 | 0.37389655 | gene458 | 0.773924039 | gene288 | 1.319153967 |
| 138 | gene124 | 0.625089 | 9.203277 | 0.06792 | gene146 | 0.374303405 | gene55 | 0.776402298 | gene445 | 1.319691762 |
| 139 | gene169 | 0.609418 | 8.957367 | 0.068035 | gene117 | 0.376392187 | gene110 | 0.77861511 | gene302 | 1.321153833 |
| 140 | gene211 | 0.635792 | 9.259794 | 0.068662 | gene338 | 0.378106948 | gene169 | 0.781698575 | gene314 | 1.325314372 |
| 141 | gene430 | 0.652274 | 9.402468 | 0.069373 | gene391 | 0.378325731 | gene66 | 0.782619849 | gene0 | 1.331915665 |
| 142 | gene180 | 0.627148 | 9.006883 | 0.06963 | gene424 | 0.379137396 | gene13 | 0.78303177 | gene135 | 1.332884604 |
| 143 | gene207 | 0.647564 | 9.295203 | 0.069666 | gene199 | 0.381319529 | gene397 | 0.783637147 | gene267 | 1.332900032 |
| 144 | gene208 | 0.631693 | 9.059422 | 0.069728 | gene194 | 0.381426233 | gene180 | 0.785596462 | gene454 | 1.333395518 |
| 145 | gene445 | 0.63666 | 9.110655 | 0.069881 | gene192 | 0.38170727 | gene445 | 0.786907709 | gene123 | 1.333674969 |
| 146 | gene460 | 0.656552 | 9.351252 | 0.07021 | gene86 | 0.382816773 | gene314 | 0.787162806 | gene295 | 1.334988121 |
| 147 | gene255 | 0.615446 | 8.761603 | 0.070244 | gene161 | 0.3830753 | gene11 | 0.787218355 | gene146 | 1.33772228 |
| 148 | gene123 | 0.653465 | 9.26713 | 0.070514 | gene422 | 0.383643125 | gene220 | 0.787363787 | gene127 | 1.337746891 |
| 149 | gene288 | 0.627881 | 8.883693 | 0.070678 | gene102 | 0.384209592 | gene135 | 0.788316234 | gene71 | 1.339012824 |
| 150 | gene229 | 0.65784 | 9.274891 | 0.070927 | gene124 | 0.38476498 | gene137 | 0.788416943 | gene303 | 1.339178113 |
| 151 | gene487 | 0.636299 | 8.970894 | 0.070929 | gene67 | 0.386581202 | gene320 | 0.78857699 | gene66 | 1.342073776 |
| 152 | gene232 | 0.651344 | 9.148612 | 0.071196 | gene362 | 0.386674727 | gene211 | 0.789607338 | gene442 | 1.342352037 |
| 153 | gene320 | 0.638596 | 8.968043 | 0.071208 | gene287 | 0.388099232 | gene277 | 0.793558181 | gene194 | 1.345718235 |
| 154 | gene369 | 0.631654 | 8.867957 | 0.071229 | gene128 | 0.388365853 | gene90 | 0.794458117 | gene412 | 1.346753936 |
| 155 | gene305 | 0.650116 | 9.040757 | 0.071909 | gene183 | 0.389479786 | gene398 | 0.795392611 | gene331 | 1.348797906 |
| 156 | gene338 | 0.674319 | 9.375862 | 0.071921 | gene37 | 0.389821561 | gene410 | 0.795982828 | gene338 | 1.351930357 |
| 157 | gene387 | 0.633306 | 8.802425 | 0.071947 | gene172 | 0.390417871 | gene318 | 0.798037381 | gene165 | 1.35506151 |
| 158 | gene277 | 0.610383 | 8.472173 | 0.072046 | gene249 | 0.390761536 | gene302 | 0.798316798 | gene227 | 1.359351821 |
| 159 | gene146 | 0.625287 | 8.65019 | 0.072286 | gene226 | 0.390823314 | gene165 | 0.79889262 | gene218 | 1.359531158 |
| 160 | gene455 | 0.646991 | 8.942941 | 0.072347 | gene135 | 0.391099536 | gene423 | 0.799716494 | gene280 | 1.359666942 |
| 161 | gene120 | 0.665042 | 9.155697 | 0.072637 | gene469 | 0.391227954 | gene216 | 0.800670384 | gene191 | 1.360041969 |
| 162 | gene176 | 0.675221 | 9.277494 | 0.072781 | gene288 | 0.391276248 | gene176 | 0.802421459 | gene320 | 1.360397902 |
| 163 | gene410 | 0.682493 | 9.347383 | 0.073014 | gene104 | 0.391437526 | gene207 | 0.802893245 | gene392 | 1.363194016 |
| 164 | gene227 | 0.683754 | 9.352516 | 0.073109 | gene25 | 0.391626815 | gene67 | 0.803181301 | gene61 | 1.364514376 |
| 165 | gene128 | 0.672554 | 9.181155 | 0.073254 | gene374 | 0.396257733 | gene124 | 0.805345074 | gene253 | 1.369533784 |
| 166 | gene374 | 0.680313 | 9.259082 | 0.073475 | gene137 | 0.396312606 | gene399 | 0.806787924 | gene318 | 1.370202122 |
| 167 | gene424 | 0.647341 | 8.800349 | 0.073559 | gene24 | 0.397228167 | gene442 | 0.806868179 | gene231 | 1.370939465 |
| 168 | gene280 | 0.664414 | 9.027742 | 0.073597 | gene211 | 0.398391013 | gene374 | 0.807145306 | gene460 | 1.37099317 |
| 169 | gene303 | 0.649247 | 8.805604 | 0.073731 | gene52 | 0.398400123 | gene146 | 0.80731377 | gene56 | 1.373507995 |
| 170 | gene412 | 0.6363 | 8.56342 | 0.074304 | gene289 | 0.398887086 | gene435 | 0.807520293 | gene424 | 1.373890983 |
| 171 | gene289 | 0.65064 | 8.749356 | 0.074364 | gene351 | 0.399341999 | gene338 | 0.807994004 | gene176 | 1.374150413 |
| 172 | gene212 | 0.681043 | 9.098806 | 0.07485 | gene125 | 0.399624756 | gene289 | 0.808177151 | gene487 | 1.374984939 |
| 173 | gene423 | 0.677218 | 9.019222 | 0.075086 | gene415 | 0.399708078 | gene430 | 0.808299397 | gene455 | 1.378967112 |
| 174 | gene323 | 0.684068 | 9.089716 | 0.075257 | gene310 | 0.400371787 | gene303 | 0.810746037 | gene423 | 1.379013918 |
| 175 | gene135 | 0.677319 | 8.981808 | 0.07541 | gene71 | 0.400898385 | gene487 | 0.811022053 | gene171 | 1.380572981 |
| 176 | gene165 | 0.676716 | 8.967152 | 0.075466 | gene178 | 0.400973379 | gene412 | 0.811393027 | gene435 | 1.380954035 |
| 177 | gene439 | 0.676875 | 8.963107 | 0.075518 | gene423 | 0.402527808 | gene295 | 0.811658494 | gene367 | 1.381317871 |
| 178 | gene125 | 0.705699 | 9.328961 | 0.075646 | gene435 | 0.402887178 | gene422 | 0.811665611 | gene122 | 1.384498563 |
| 179 | gene127 | 0.670492 | 8.861495 | 0.075663 | gene33 | 0.404176094 | gene246 | 0.813794033 | gene169 | 1.384556638 |
| 180 | gene470 | 0.684039 | 9.006602 | 0.075949 | gene498 | 0.404253216 | gene369 | 0.815288184 | gene35 | 1.385146193 |
| 181 | gene267 | 0.69909 | 9.181825 | 0.076138 | gene267 | 0.404435806 | gene204 | 0.816699629 | gene204 | 1.387926225 |
| 182 | gene171 | 0.695997 | 9.109995 | 0.076399 | gene305 | 0.404595244 | gene33 | 0.817064693 | gene369 | 1.387998898 |
| 183 | gene231 | 0.655567 | 8.570441 | 0.076492 | gene204 | 0.404635004 | gene288 | 0.818625767 | gene180 | 1.38805964 |
| 184 | gene153 | 0.682486 | 8.910924 | 0.07659 | gene180 | 0.40499321 | gene392 | 0.819634835 | gene125 | 1.391942563 |
| 185 | gene214 | 0.680168 | 8.874836 | 0.07664 | gene250 | 0.405297582 | gene56 | 0.821297371 | gene255 | 1.392014256 |
| 186 | gene311 | 0.671873 | 8.751224 | 0.076775 | gene127 | 0.4053364 | gene138 | 0.821526987 | gene95 | 1.39240636 |
| 187 | gene147 | 0.651146 | 8.472383 | 0.076855 | gene323 | 0.405621786 | gene253 | 0.822784014 | gene67 | 1.392744536 |
| 188 | gene200 | 0.699617 | 9.074383 | 0.077098 | gene295 | 0.405641312 | gene5 | 0.82288322 | gene183 | 1.392756944 |
| 189 | gene442 | 0.634968 | 8.209995 | 0.077341 | gene443 | 0.406670973 | gene229 | 0.823955031 | gene178 | 1.395703719 |
| 190 | gene432 | 0.695441 | 8.944093 | 0.077754 | gene473 | 0.406775557 | gene455 | 0.824092639 | gene87 | 1.396680307 |
| 191 | gene251 | 0.671764 | 8.628962 | 0.07785 | gene90 | 0.406785294 | gene493 | 0.824295633 | gene439 | 1.396851332 |
| 192 | gene185 | 0.6672 | 8.552747 | 0.07801 | gene318 | 0.407187085 | gene125 | 0.824623497 | gene398 | 1.397154123 |
| 193 | gene163 | 0.661393 | 8.442091 | 0.078345 | gene13 | 0.407760046 | gene232 | 0.825274806 | gene216 | 1.39737476 |
| 194 | gene106 | 0.667902 | 8.524511 | 0.078351 | gene419 | 0.407825952 | gene161 | 0.827103269 | gene147 | 1.39850546 |
| 195 | gene246 | 0.69626 | 8.867978 | 0.078514 | gene442 | 0.408170329 | gene147 | 0.827183927 | gene94 | 1.399440395 |
| 196 | gene337 | 0.7064 | 8.952979 | 0.078901 | gene200 | 0.410104267 | gene171 | 0.827652816 | gene124 | 1.399762365 |
| 197 | gene493 | 0.73126 | 9.24028 | 0.079138 | gene445 | 0.412066868 | gene150 | 0.82837491 | gene232 | 1.40196141 |
| 198 | gene299 | 0.703906 | 8.869857 | 0.079359 | gene139 | 0.413468232 | gene120 | 0.830888876 | gene157 | 1.404240921 |
| 199 | gene353 | 0.680958 | 8.53774 | 0.079759 | gene206 | 0.413865008 | gene460 | 0.83173488 | gene353 | 1.405603196 |
| 200 | gene343 | 0.731406 | 9.114233 | 0.080249 | gene493 | 0.415157533 | gene153 | 0.832094283 | gene422 | 1.407564272 |
| 201 | gene206 | 0.689697 | 8.557122 | 0.080599 | gene428 | 0.415223414 | gene419 | 0.832632855 | gene352 | 1.407916648 |
| 202 | gene322 | 0.727605 | 9.021938 | 0.080648 | gene30 | 0.415907108 | gene170 | 0.832797102 | gene387 | 1.408561067 |
| 203 | gene193 | 0.712018 | 8.807526 | 0.080842 | gene66 | 0.4167638 | gene128 | 0.833384664 | gene274 | 1.409373746 |
| 204 | gene399 | 0.753953 | 9.321727 | 0.080881 | gene110 | 0.416970875 | gene127 | 0.833922223 | gene243 | 1.410699008 |
| 205 | gene435 | 0.696561 | 8.606158 | 0.080938 | gene56 | 0.417495165 | gene206 | 0.833977595 | gene425 | 1.410827345 |
| 206 | gene447 | 0.71803 | 8.870205 | 0.080949 | gene103 | 0.417866625 | gene95 | 0.834043399 | gene64 | 1.412240205 |
| 207 | gene184 | 0.724892 | 8.944211 | 0.081046 | gene171 | 0.417998582 | gene447 | 0.834169842 | gene150 | 1.41365866 |
| 208 | gene149 | 0.689182 | 8.49845 | 0.081095 | gene328 | 0.418435028 | gene65 | 0.83440018 | gene229 | 1.414501311 |
| 209 | gene204 | 0.730021 | 8.975156 | 0.081338 | gene136 | 0.418457836 | gene250 | 0.834895427 | gene200 | 1.414693078 |
| 210 | gene315 | 0.73703 | 9.061328 | 0.081338 | gene488 | 0.419618769 | gene255 | 0.835324118 | gene315 | 1.415649657 |
| 211 | gene245 | 0.76012 | 9.288598 | 0.081834 | gene165 | 0.419800928 | gene71 | 0.835899254 | gene212 | 1.415725801 |
| 212 | gene103 | 0.75974 | 9.262324 | 0.082025 | gene335 | 0.419935658 | gene87 | 0.835965526 | gene246 | 1.416022849 |
| 213 | gene138 | 0.756179 | 9.183371 | 0.082342 | gene343 | 0.422198511 | gene266 | 0.836296194 | gene185 | 1.416720709 |
| 214 | gene495 | 0.736628 | 8.943049 | 0.082369 | gene313 | 0.422489814 | gene231 | 0.836564018 | gene86 | 1.417216013 |
| 215 | gene398 | 0.75014 | 9.10304 | 0.082405 | gene246 | 0.422729324 | gene86 | 0.836844686 | gene79 | 1.417372904 |
| 216 | gene216 | 0.751898 | 9.069758 | 0.082902 | gene280 | 0.422917915 | gene106 | 0.838282872 | gene495 | 1.418217021 |
| 217 | gene143 | 0.755362 | 9.106157 | 0.082951 | gene468 | 0.424141973 | gene353 | 0.839808941 | gene128 | 1.419760677 |
| 218 | gene436 | 0.758445 | 9.119259 | 0.08317 | gene232 | 0.424885961 | gene305 | 0.840054476 | gene120 | 1.420880523 |
| 219 | gene274 | 0.750928 | 9.015648 | 0.083292 | gene455 | 0.425687293 | gene183 | 0.840743229 | gene208 | 1.421664775 |
| 220 | gene276 | 0.726929 | 8.723702 | 0.083328 | gene416 | 0.425894514 | gene488 | 0.841071562 | gene266 | 1.422089622 |
| 221 | gene448 | 0.734614 | 8.814719 | 0.08334 | gene218 | 0.426150534 | gene212 | 0.843008125 | gene54 | 1.422612798 |
| 222 | gene243 | 0.742817 | 8.899465 | 0.083468 | gene157 | 0.427965228 | gene208 | 0.843087488 | gene334 | 1.42326226 |
| 223 | gene418 | 0.736889 | 8.806194 | 0.083678 | gene176 | 0.428004818 | gene104 | 0.843231164 | gene436 | 1.423394325 |
| 224 | gene472 | 0.69036 | 8.246995 | 0.083711 | gene452 | 0.428115291 | gene103 | 0.843433998 | gene289 | 1.424566828 |
| 225 | gene253 | 0.720546 | 8.58195 | 0.083961 | gene118 | 0.428174936 | gene218 | 0.843784325 | gene251 | 1.424862055 |
| 226 | gene352 | 0.732106 | 8.718863 | 0.083968 | gene447 | 0.428207307 | gene280 | 0.844679001 | gene207 | 1.424907767 |
| 227 | gene100 | 0.758188 | 9.023162 | 0.084027 | gene255 | 0.430619384 | gene27 | 0.845043781 | gene65 | 1.424922362 |
| 228 | gene209 | 0.748712 | 8.877473 | 0.084338 | gene185 | 0.430716 | gene37 | 0.845306042 | gene106 | 1.425280379 |
| 229 | gene114 | 0.736963 | 8.711922 | 0.084593 | gene212 | 0.430957105 | gene335 | 0.845334002 | gene92 | 1.425714721 |
| 230 | gene420 | 0.746231 | 8.79385 | 0.084858 | gene303 | 0.431127043 | gene425 | 0.845356453 | gene37 | 1.426205658 |
| 231 | gene488 | 0.780727 | 9.181589 | 0.085032 | gene439 | 0.432432902 | gene197 | 0.84709944 | gene26 | 1.426632117 |
| 232 | gene230 | 0.775492 | 9.101447 | 0.085205 | gene425 | 0.432664907 | gene436 | 0.848552785 | gene376 | 1.426677576 |
| 233 | gene157 | 0.786686 | 9.231438 | 0.085218 | gene95 | 0.432975927 | gene434 | 0.849995199 | gene410 | 1.427033058 |
| 234 | gene365 | 0.752011 | 8.816006 | 0.085301 | gene253 | 0.434906726 | gene99 | 0.850855756 | gene32 | 1.427174386 |
| 235 | gene425 | 0.771731 | 9.026863 | 0.085493 | gene197 | 0.435331112 | gene185 | 0.851928382 | gene361 | 1.427724677 |
| 236 | gene456 | 0.741539 | 8.66896 | 0.08554 | gene189 | 0.435962679 | gene114 | 0.852593979 | gene337 | 1.428651856 |
| 237 | gene249 | 0.801508 | 9.349018 | 0.085732 | gene122 | 0.436681756 | gene32 | 0.853391617 | gene374 | 1.429643928 |
| 238 | gene183 | 0.795964 | 9.276878 | 0.085801 | gene412 | 0.436876983 | gene432 | 0.853989587 | gene323 | 1.434761137 |
| 239 | gene307 | 0.743445 | 8.649338 | 0.085954 | gene266 | 0.437021019 | gene149 | 0.854267835 | gene57 | 1.43578183 |
| 240 | gene416 | 0.79286 | 9.216943 | 0.086022 | gene143 | 0.439693514 | gene122 | 0.854647714 | gene305 | 1.438566396 |
| 241 | gene471 | 0.792001 | 9.203301 | 0.086056 | gene279 | 0.439811162 | gene334 | 0.856639124 | gene427 | 1.439126664 |
| 242 | gene334 | 0.78046 | 9.047567 | 0.086262 | gene320 | 0.439915791 | gene473 | 0.856889585 | gene55 | 1.439734223 |
| 243 | gene394 | 0.784593 | 9.092433 | 0.086291 | gene376 | 0.44009055 | gene299 | 0.857015129 | gene214 | 1.440850142 |
| 244 | gene475 | 0.757877 | 8.756992 | 0.086545 | gene48 | 0.440373275 | gene157 | 0.857349798 | gene5 | 1.441495935 |
| 245 | gene428 | 0.790084 | 9.126651 | 0.086569 | gene406 | 0.440412354 | gene26 | 0.857486787 | gene170 | 1.442700064 |
| 246 | gene122 | 0.7548 | 8.696976 | 0.086789 | gene38 | 0.440417795 | gene439 | 0.85765791 | gene83 | 1.443824924 |
| 247 | gene379 | 0.740709 | 8.519602 | 0.086942 | gene471 | 0.440843916 | gene406 | 0.859013028 | gene49 | 1.444823784 |
| 248 | gene173 | 0.765581 | 8.791826 | 0.087079 | gene414 | 0.441575051 | gene100 | 0.85986233 | gene493 | 1.44492609 |
| 249 | gene159 | 0.781459 | 8.947635 | 0.087337 | gene208 | 0.442129525 | gene192 | 0.860292993 | gene99 | 1.445830664 |
| 250 | gene417 | 0.757997 | 8.66475 | 0.087481 | gene434 | 0.443884122 | gene471 | 0.860485263 | gene470 | 1.445893317 |
| 251 | gene266 | 0.785558 | 8.979566 | 0.087483 | gene245 | 0.444646684 | gene456 | 0.860644844 | gene393 | 1.44734773 |
| 252 | gene376 | 0.801235 | 9.145889 | 0.087606 | gene106 | 0.444930781 | gene366 | 0.860772571 | gene78 | 1.447496491 |
| 253 | gene476 | 0.757724 | 8.646251 | 0.087636 | gene114 | 0.448201005 | gene184 | 0.861044096 | gene244 | 1.447936137 |
| 254 | gene335 | 0.806653 | 9.203787 | 0.087644 | gene150 | 0.448376571 | gene35 | 0.861734223 | gene250 | 1.448481482 |
| 255 | gene393 | 0.771818 | 8.7946 | 0.08776 | gene195 | 0.448409333 | gene244 | 0.861902663 | gene143 | 1.449854183 |
| 256 | gene178 | 0.815444 | 9.291457 | 0.087763 | gene27 | 0.450437815 | gene94 | 0.862324246 | gene459 | 1.450141939 |
| 257 | gene247 | 0.779962 | 8.863631 | 0.087996 | gene446 | 0.451051482 | gene83 | 0.862761281 | gene259 | 1.450456166 |
| 258 | gene397 | 0.783797 | 8.897419 | 0.088093 | gene361 | 0.451332767 | gene92 | 0.86331849 | gene397 | 1.450481402 |
| 259 | gene167 | 0.759017 | 8.583789 | 0.088424 | gene175 | 0.451972239 | gene78 | 0.863477482 | gene351 | 1.450687619 |
| 260 | gene275 | 0.779271 | 8.781166 | 0.088743 | gene132 | 0.452137708 | gene498 | 0.864238309 | gene206 | 1.450864818 |
| 261 | gene104 | 0.811403 | 9.086022 | 0.089302 | gene436 | 0.45240231 | gene323 | 0.864866037 | gene447 | 1.452389993 |
| 262 | gene341 | 0.786752 | 8.808913 | 0.089313 | gene5 | 0.453387289 | gene79 | 0.866616994 | gene301 | 1.452585648 |
| 263 | gene406 | 0.801763 | 8.968766 | 0.089395 | gene54 | 0.453453765 | gene310 | 0.86667819 | gene209 | 1.453672701 |
| 264 | gene259 | 0.798915 | 8.932766 | 0.089437 | gene393 | 0.453804737 | gene352 | 0.86667921 | gene322 | 1.453896921 |
| 265 | gene484 | 0.817448 | 9.136804 | 0.089468 | gene32 | 0.454002099 | gene387 | 0.866952823 | gene472 | 1.45578304 |
| 266 | gene218 | 0.696961 | 7.756645 | 0.089853 | gene254 | 0.45436426 | gene393 | 0.867077329 | gene153 | 1.45586793 |
| 267 | gene164 | 0.813445 | 9.019689 | 0.090185 | gene65 | 0.455028264 | gene200 | 0.867530497 | gene59 | 1.458209468 |
| 268 | gene244 | 0.827484 | 9.125461 | 0.090679 | gene64 | 0.456344208 | gene178 | 0.867956111 | gene184 | 1.458217586 |
| 269 | gene434 | 0.789635 | 8.667548 | 0.091102 | gene334 | 0.456373231 | gene274 | 0.868783392 | gene417 | 1.458778745 |
| 270 | gene350 | 0.814537 | 8.899919 | 0.091522 | gene59 | 0.45695426 | gene328 | 0.870834975 | gene307 | 1.460021541 |
| 271 | gene239 | 0.826809 | 8.968041 | 0.092195 | gene57 | 0.459078806 | gene417 | 0.871054869 | gene311 | 1.460540575 |
| 272 | gene306 | 0.790869 | 8.553637 | 0.09246 | gene231 | 0.460972028 | gene24 | 0.871423614 | gene93 | 1.461525981 |
| 273 | gene294 | 0.826798 | 8.936902 | 0.092515 | gene241 | 0.461038018 | gene2 | 0.871424565 | gene434 | 1.4617504 |
| 274 | gene427 | 0.822389 | 8.881709 | 0.092594 | gene99 | 0.461513009 | gene59 | 0.872435722 | gene335 | 1.464096322 |
| 275 | gene366 | 0.833754 | 9.000673 | 0.092632 | gene301 | 0.461747408 | gene57 | 0.874393002 | gene448 | 1.465462014 |
| 276 | gene152 | 0.827771 | 8.916683 | 0.092834 | gene235 | 0.462926636 | gene88 | 0.874461212 | gene104 | 1.466271716 |
| 277 | gene170 | 0.803491 | 8.652583 | 0.092861 | gene487 | 0.463286214 | gene376 | 0.875006482 | gene328 | 1.466483654 |
| 278 | gene477 | 0.823846 | 8.866619 | 0.092915 | gene417 | 0.464056544 | gene472 | 0.875341958 | gene149 | 1.466947074 |
| 279 | gene440 | 0.770032 | 8.273464 | 0.093072 | gene369 | 0.465028664 | gene495 | 0.875858174 | gene27 | 1.467409768 |
| 280 | gene132 | 0.845438 | 9.066568 | 0.093248 | gene497 | 0.465249347 | gene48 | 0.876470788 | gene197 | 1.470405347 |
| 281 | gene452 | 0.836379 | 8.969213 | 0.09325 | gene259 | 0.466754826 | gene322 | 0.87916589 | gene343 | 1.471953986 |
| 282 | gene336 | 0.818353 | 8.760153 | 0.093418 | gene23894 | 0.46708195 | gene251 | 0.879215511 | gene2 | 1.472710523 |
| 283 | gene326 | 0.846419 | 9.055237 | 0.093473 | gene353 | 0.467515197 | gene97 | 0.87922912 | gene488 | 1.474279476 |
| 284 | gene107 | 0.805465 | 8.604301 | 0.093612 | gene6845 | 0.467612298 | gene215 | 0.879413531 | gene432 | 1.475389633 |
| 285 | gene250 | 0.860637 | 9.167989 | 0.093874 | gene170 | 0.468499418 | gene214 | 0.880056002 | gene421 | 1.475505104 |
| 286 | gene328 | 0.867913 | 9.233842 | 0.093993 | gene420 | 0.468621035 | gene136 | 0.880358382 | gene22 | 1.477554125 |
| 287 | gene201 | 0.867522 | 9.216556 | 0.094126 | gene14522 | 0.468639423 | gene428 | 0.881663444 | gene114 | 1.477669969 |
| 288 | gene271 | 0.82349 | 8.741849 | 0.094201 | gene1878 | 0.468816356 | gene47 | 0.881909549 | gene419 | 1.478457493 |
| 289 | gene468 | 0.845075 | 8.964495 | 0.094269 | gene43244 | 0.468911685 | gene241 | 0.881978892 | gene47 | 1.480399397 |
| 290 | gene361 | 0.865708 | 9.183063 | 0.094272 | gene120 | 0.469114658 | gene53 | 0.882527246 | gene394 | 1.48109276 |
| 291 | gene356 | 0.83044 | 8.783997 | 0.09454 | gene47545 | 0.469547146 | gene337 | 0.882571855 | gene294 | 1.483026804 |
| 292 | gene202 | 0.735624 | 7.777121 | 0.094588 | gene3725 | 0.469602315 | gene259 | 0.88268443 | gene239 | 1.483478616 |
| 293 | gene292 | 0.752413 | 7.949089 | 0.094654 | gene42896 | 0.469785782 | gene40 | 0.883132323 | gene420 | 1.484144535 |
| 294 | gene310 | 0.875348 | 9.244833 | 0.094685 | gene14751 | 0.46982705 | gene51 | 0.883588307 | gene24 | 1.484716981 |
| 295 | gene177 | 0.853001 | 8.996091 | 0.094819 | gene251 | 0.470217263 | gene307 | 0.883633506 | gene456 | 1.48515668 |
| 296 | gene351 | 0.875613 | 9.233554 | 0.094829 | gene24669 | 0.470285466 | gene249 | 0.88370238 | gene82 | 1.486670472 |
| 297 | gene372 | 0.828042 | 8.718618 | 0.094974 | gene33517 | 0.47093646 | gene175 | 0.883896277 | gene164 | 1.486710892 |
| 298 | gene272 | 0.81639 | 8.588722 | 0.095054 | gene19457 | 0.471207847 | gene326 | 0.883960624 | gene299 | 1.487163419 |
| 299 | gene145 | 0.84742 | 8.860343 | 0.095642 | gene23684 | 0.47131377 | gene452 | 0.885428138 | gene366 | 1.487756886 |
| 300 | gene459 | 0.803817 | 8.379312 | 0.095929 | gene277 | 0.471554533 | gene235 | 0.887167978 | gene88 | 1.488123655 |
| 301 | gene414 | 0.8599 | 8.931652 | 0.096276 | gene8230 | 0.471601626 | gene230 | 0.889279771 | gene63 | 1.489069729 |
| 302 | gene161 | 0.901267 | 9.351437 | 0.096377 | gene12162 | 0.471678587 | gene279 | 0.88987362 | gene103 | 1.489076231 |
| 303 | gene304 | 0.864811 | 8.964304 | 0.096473 | gene43097 | 0.47195252 | gene379 | 0.890944329 | gene53 | 1.490772813 |
| 304 | gene308 | 0.81586 | 8.449066 | 0.096562 | gene244 | 0.471975763 | gene68 | 0.891178427 | gene97 | 1.490932141 |
| 305 | gene321 | 0.854524 | 8.831586 | 0.096758 | gene94 | 0.472258367 | gene64 | 0.891420738 | gene399 | 1.491951203 |
| 306 | gene497 | 0.873733 | 9.025219 | 0.09681 | gene230 | 0.472531283 | gene243 | 0.891499837 | gene471 | 1.492136143 |
| 307 | gene291 | 0.874661 | 8.959793 | 0.097621 | gene46459 | 0.472588295 | gene173 | 0.891676161 | gene138 | 1.493568391 |
| 308 | gene228 | 0.844266 | 8.638742 | 0.09773 | gene49425 | 0.472681759 | gene470 | 0.892999107 | gene80 | 1.493834586 |
| 309 | gene401 | 0.85867 | 8.737563 | 0.098273 | gene40030 | 0.472917171 | gene301 | 0.893947476 | gene163 | 1.493972387 |
| 310 | gene421 | 0.879326 | 8.885145 | 0.098966 | gene151 | 0.472975968 | gene132 | 0.894552588 | gene193 | 1.494195953 |
| 311 | gene203 | 0.836858 | 8.402181 | 0.0996 | gene432 | 0.473121433 | gene245 | 0.894788184 | gene195 | 1.494325449 |
| 312 | gene419 | 0.905193 | 9.082334 | 0.099665 | gene18467 | 0.473293943 | gene315 | 0.895053935 | gene379 | 1.494932409 |
| 313 | gene496 | 0.903923 | 9.066618 | 0.099698 | gene311 | 0.473325968 | gene54 | 0.895489319 | gene30 | 1.495041312 |
| 314 | gene278 | 0.869053 | 8.706734 | 0.099814 | gene149 | 0.473574885 | gene448 | 0.897233382 | gene496 | 1.495951695 |
| 315 | gene136 | 0.907395 | 9.068083 | 0.100065 | gene14156 | 0.473661153 | gene484 | 0.898344373 | gene276 | 1.496382595 |
| 316 | gene388 | 0.811993 | 8.098962 | 0.100259 | gene33834 | 0.473708988 | gene80 | 0.898559617 | gene416 | 1.498093774 |
| 317 | gene373 | 0.865372 | 8.619766 | 0.100394 | gene27293 | 0.473851202 | gene311 | 0.899036359 | gene245 | 1.49834185 |
| 318 | gene235 | 0.870955 | 8.667581 | 0.100484 | gene33225 | 0.473926225 | gene351 | 0.900207121 | gene161 | 1.498745855 |
| 319 | gene268 | 0.863485 | 8.592693 | 0.100491 | gene6887 | 0.47392681 | gene189 | 0.900235159 | gene173 | 1.498900939 |
| 320 | gene408 | 0.879226 | 8.746405 | 0.100524 | gene11216 | 0.474023108 | gene343 | 0.900439318 | gene43 | 1.501210156 |
| 321 | gene192 | 0.923736 | 9.15504 | 0.100899 | gene12497 | 0.474093536 | gene202 | 0.900740969 | gene428 | 1.502699473 |
| 322 | gene181 | 0.881375 | 8.714037 | 0.101144 | gene153 | 0.474100749 | gene313 | 0.900903424 | gene158 | 1.502907425 |
| 323 | gene413 | 0.869309 | 8.568335 | 0.101456 | gene394 | 0.474284211 | gene459 | 0.901015787 | gene100 | 1.504674482 |
| 324 | gene301 | 0.885861 | 8.689777 | 0.101943 | gene9850 | 0.474408222 | gene254 | 0.901873139 | gene418 | 1.505001777 |
| 325 | gene407 | 0.85394 | 8.37487 | 0.101965 | gene49 | 0.474502469 | gene156 | 0.901950315 | gene151 | 1.505193406 |
| 326 | gene450 | 0.934363 | 9.161571 | 0.101987 | gene22344 | 0.474707408 | gene349 | 0.902250152 | gene479 | 1.506024741 |
| 327 | gene257 | 0.901797 | 8.834667 | 0.102075 | gene38265 | 0.474775557 | gene30 | 0.902469455 | gene313 | 1.506947959 |
| 328 | gene362 | 0.956066 | 9.353889 | 0.102211 | gene201 | 0.475165463 | gene63 | 0.90275235 | gene68 | 1.507137808 |
| 329 | gene248 | 0.856581 | 8.374891 | 0.10228 | gene31481 | 0.475400624 | gene158 | 0.90309365 | gene341 | 1.507180962 |
| 330 | gene309 | 0.907682 | 8.850134 | 0.102561 | gene141 | 0.475514405 | gene72 | 0.903342345 | gene336 | 1.50796912 |
| 331 | gene296 | 0.88306 | 8.607028 | 0.102598 | gene11796 | 0.475593937 | gene141 | 0.904010674 | gene406 | 1.508977628 |
| 332 | gene465 | 0.826679 | 8.045631 | 0.102749 | gene17470 | 0.475661987 | gene421 | 0.904496911 | gene48 | 1.510439353 |
| 333 | gene382 | 0.904244 | 8.786928 | 0.102908 | gene16957 | 0.476010724 | gene143 | 0.905460978 | gene450 | 1.510590181 |
| 334 | gene151 | 0.897991 | 8.718676 | 0.102996 | gene76 | 0.476231009 | gene195 | 0.905733845 | gene396 | 1.51075142 |
| 335 | gene344 | 0.90856 | 8.818202 | 0.103032 | gene37308 | 0.476282705 | gene49 | 0.905886614 | gene215 | 1.511511753 |
| 336 | gene175 | 0.844767 | 8.184789 | 0.103212 | gene35 | 0.476359652 | gene497 | 0.906548903 | gene365 | 1.512504216 |
| 337 | gene116 | 0.911617 | 8.820612 | 0.103351 | gene19896 | 0.476653989 | gene418 | 0.90672986 | gene23 | 1.512724877 |
| 338 | gene457 | 0.907726 | 8.782945 | 0.103351 | gene291 | 0.476896938 | gene475 | 0.906774651 | gene189 | 1.514158969 |
| 339 | gene238 | 0.915621 | 8.849587 | 0.103465 | gene19479 | 0.476916944 | gene416 | 0.90687189 | gene321 | 1.514219049 |
| 340 | gene141 | 0.923878 | 8.927304 | 0.103489 | gene41689 | 0.4772492 | gene93 | 0.907549114 | gene109 | 1.514528137 |
| 341 | gene241 | 0.907674 | 8.768944 | 0.10351 | gene48200 | 0.477251396 | gene466 | 0.90759952 | gene72 | 1.514759907 |
| 342 | gene182 | 0.924668 | 8.898705 | 0.10391 | gene44226 | 0.477393297 | gene333 | 0.907684018 | gene241 | 1.515668111 |
| 343 | gene189 | 0.96106 | 9.244609 | 0.103959 | gene41504 | 0.477406646 | gene201 | 0.908216087 | gene292 | 1.516596316 |
| 344 | gene195 | 0.951948 | 9.154868 | 0.103983 | gene35915 | 0.477539006 | gene450 | 0.908891934 | gene230 | 1.517595643 |
| 345 | gene156 | 0.94545 | 9.089741 | 0.104013 | gene30046 | 0.477614103 | gene271 | 0.908995974 | gene8 | 1.518178708 |
| 346 | gene168 | 0.917654 | 8.813042 | 0.104125 | gene17622 | 0.477622324 | gene118 | 0.909035323 | gene249 | 1.518951455 |
| 347 | gene118 | 0.954421 | 9.159942 | 0.104195 | gene15433 | 0.477737695 | gene494 | 0.909527404 | gene145 | 1.518952631 |
| 348 | gene293 | 0.934976 | 8.972273 | 0.104207 | gene19641 | 0.477850395 | gene38 | 0.910691941 | gene473 | 1.519726625 |
| 349 | gene313 | 0.967667 | 9.279843 | 0.104276 | gene22176 | 0.477927843 | gene228 | 0.91081288 | gene467 | 1.520197052 |
| 350 | gene467 | 0.919752 | 8.817026 | 0.104315 | gene177 | 0.477962255 | gene76 | 0.910888952 | gene91 | 1.521782354 |
| 351 | gene213 | 0.89893 | 8.613682 | 0.104361 | gene35919 | 0.477979637 | gene361 | 0.911098429 | gene304 | 1.521879599 |
| 352 | gene197 | 0.963253 | 9.224404 | 0.104424 | gene7568 | 0.478039286 | gene291 | 0.911355643 | gene201 | 1.522732653 |
| 353 | gene346 | 0.901895 | 8.627369 | 0.104539 | gene4453 | 0.478068797 | gene209 | 0.911479989 | gene10 | 1.523386377 |
| 354 | gene473 | 0.926259 | 8.84867 | 0.104678 | gene27304 | 0.478230369 | gene193 | 0.911523775 | gene38 | 1.523529667 |
| 355 | gene333 | 0.93528 | 8.934707 | 0.104679 | gene23532 | 0.478253829 | gene276 | 0.911740995 | gene40 | 1.523904152 |
| 356 | gene359 | 0.914436 | 8.728996 | 0.104758 | gene27403 | 0.478320374 | gene22 | 0.912617929 | gene51 | 1.524238038 |
| 357 | gene256 | 0.883104 | 8.42395 | 0.104833 | gene42048 | 0.4787368 | gene420 | 0.913561847 | gene401 | 1.52466263 |
| 358 | gene479 | 0.903069 | 8.609626 | 0.104891 | gene21014 | 0.478753576 | gene446 | 0.914049372 | gene42 | 1.525151012 |
| 359 | gene240 | 0.933423 | 8.895262 | 0.104935 | gene457 | 0.478754431 | gene297 | 0.914145632 | gene372 | 1.527136736 |
| 360 | gene446 | 0.951557 | 9.039031 | 0.105272 | gene17308 | 0.478820994 | gene275 | 0.915106149 | gene484 | 1.527320385 |
| 361 | gene263 | 0.93736 | 8.892515 | 0.10541 | gene13544 | 0.478969495 | gene304 | 0.916110095 | gene498 | 1.527538593 |
| 362 | gene332 | 0.930104 | 8.81842 | 0.105473 | gene17713 | 0.479100233 | gene96 | 0.916805802 | gene356 | 1.528140942 |
| 363 | gene324 | 0.941639 | 8.920593 | 0.105558 | gene43859 | 0.479100722 | gene365 | 0.916849081 | gene476 | 1.528244225 |
| 364 | gene215 | 0.816851 | 7.692061 | 0.106194 | gene158 | 0.479303297 | gene394 | 0.917918119 | gene235 | 1.529064357 |
| 365 | gene466 | 0.954824 | 8.972622 | 0.106415 | gene28346 | 0.47935532 | gene468 | 0.918788706 | gene272 | 1.529183479 |
| 366 | gene396 | 0.942511 | 8.828524 | 0.106757 | gene6265 | 0.479392465 | gene82 | 0.919354577 | gene475 | 1.530120922 |
| 367 | gene390 | 0.953764 | 8.931212 | 0.10679 | gene27078 | 0.479598549 | gene477 | 0.920965943 | gene41 | 1.530766301 |
| 368 | gene330 | 0.859619 | 8.019636 | 0.107189 | gene392 | 0.479639103 | gene272 | 0.921848234 | gene175 | 1.53292473 |
| 369 | gene297 | 0.948151 | 8.839126 | 0.107267 | gene17885 | 0.4797435 | gene292 | 0.924733027 | gene310 | 1.533907926 |
| 370 | gene349 | 0.957958 | 8.905138 | 0.107574 | gene45989 | 0.479879677 | gene427 | 0.925073476 | gene326 | 1.534927896 |
| 371 | gene254 | 0.976187 | 9.074092 | 0.10758 | gene10730 | 0.479927725 | gene309 | 0.925353702 | gene152 | 1.535467057 |
| 372 | gene494 | 0.973631 | 8.966339 | 0.108587 | gene15877 | 0.480103967 | gene479 | 0.925850418 | gene159 | 1.536694687 |
| 373 | gene158 | 0.94902 | 8.726153 | 0.108756 | gene18388 | 0.480160848 | gene257 | 0.925851363 | gene136 | 1.537064959 |
| 374 | gene375 | 0.905719 | 8.324524 | 0.108801 | gene17651 | 0.480495069 | gene239 | 0.92667435 | gene350 | 1.537217768 |
| 375 | gene134 | 0.931939 | 8.55648 | 0.108916 | gene31270 | 0.480550347 | gene163 | 0.926792054 | gene202 | 1.537297987 |
| 376 | gene242 | 0.919172 | 8.43408 | 0.108983 | gene9219 | 0.480598886 | gene496 | 0.927485736 | gene7 | 1.537496191 |
| 377 | gene260 | 0.926152 | 8.492012 | 0.109062 | gene39868 | 0.480602282 | gene350 | 0.927802415 | gene141 | 1.538010873 |
| 378 | gene498 | 0.927123 | 8.44667 | 0.109762 | gene42515 | 0.480616419 | gene23 | 0.928981182 | gene31 | 1.538160579 |
| 379 | gene109 | 0.911284 | 8.297242 | 0.10983 | gene22133 | 0.480664359 | gene356 | 0.929514952 | gene228 | 1.539082618 |
| 380 | gene262 | 0.962461 | 8.757529 | 0.109901 | gene36679 | 0.480668615 | gene152 | 0.929810909 | gene254 | 1.540648047 |
| 381 | gene279 | 0.926015 | 8.42083 | 0.109967 | gene274 | 0.480752641 | gene476 | 0.930812404 | gene77 | 1.541738553 |
| 382 | gene429 | 0.953969 | 8.592472 | 0.111024 | gene24866 | 0.48076426 | gene109 | 0.931435122 | gene497 | 1.542386579 |
| 383 | gene144 | 0.945887 | 8.51121 | 0.111134 | gene23878 | 0.480986231 | gene151 | 0.931486652 | gene413 | 1.543024751 |
| 384 | gene444 | 0.955766 | 8.583329 | 0.111351 | gene9919 | 0.48099949 | gene390 | 0.931806446 | gene60 | 1.544348769 |
| 385 | gene426 | 0.979647 | 8.769946 | 0.111705 | gene11702 | 0.481189806 | gene31 | 0.932977226 | gene156 | 1.544941646 |
| 386 | gene492 | 0.949122 | 8.474543 | 0.111997 | gene26324 | 0.481259345 | gene42 | 0.934015387 | gene306 | 1.545776691 |
| 387 | gene258 | 0.985582 | 8.795603 | 0.112054 | gene13319 | 0.481329181 | gene467 | 0.935789761 | gene275 | 1.546137321 |
| 388 | gene339 | 0.978358 | 8.720863 | 0.112186 | gene36100 | 0.481478156 | gene91 | 0.935847072 | gene452 | 1.546278824 |
| 389 | gene131 | 0.929009 | 8.27317 | 0.112292 | gene15090 | 0.481492326 | gene164 | 0.935978658 | gene118 | 1.546647913 |
| 390 | gene111 | 0.914689 | 8.136084 | 0.112424 | gene26049 | 0.481534676 | gene321 | 0.936030449 | gene297 | 1.548268212 |
| 391 | gene384 | 0.957335 | 8.473399 | 0.112981 | gene23012 | 0.481539838 | gene60 | 0.936339638 | gene98 | 1.548488011 |
| 392 | gene357 | 0.975234 | 8.620532 | 0.113129 | gene16807 | 0.481546191 | gene362 | 0.937641041 | gene390 | 1.550605361 |
| 393 | gene119 | 0.974437 | 8.560261 | 0.113833 | gene46662 | 0.481569124 | gene413 | 0.937934385 | gene256 | 1.551151788 |
| 394 | gene252 | 0.943476 | 8.146548 | 0.115813 | gene34377 | 0.481582795 | gene256 | 0.938036498 | gene181 | 1.551253977 |
| 395 | gene166 | 0.938349 | 8.084295 | 0.116071 | gene39602 | 0.48179195 | gene181 | 0.938047524 | gene309 | 1.551668222 |
| 396 | gene284 | 0.959711 | 8.050217 | 0.119216 | gene35771 | 0.481814102 | gene401 | 0.938540203 | gene257 | 1.55229734 |
| 397 | gene283 | 0.975767 | 8.018395 | 0.121691 | gene48162 | 0.48197518 | gene414 | 0.938579108 | gene19 | 1.55280006 |
| 398 | gene449 | 0.985181 | 8.032723 | 0.122646 | gene40130 | 0.482036968 | gene294 | 0.939908636 | gene457 | 1.553218657 |
| 399 | gene179 | 0.959865 | 7.807317 | 0.122944 | gene365 | 0.482043297 | gene77 | 0.941513197 | gene132 | 1.553498457 |
| 400 | gene210 | 0.934811 | 7.197833 | 0.129874 | gene26830 | 0.482189275 | gene62 | 0.941573402 | gene465 | 1.556518053 |

## Table S2: Reference candidate genes top 400 list from GSV and OLIVER for the synthetic dataset 2. The rank order (GSV ID) of the genes (ID) was based on the coefficient of variation (CV). TPM avrg: TPM average. The OLIVER orders was based on CV and methods 10 and 14 calculations in the OLIVER original paper. The low-expression genes filtered out by GSV are in red. The variable genes filtered out by GSV are in orange.

| **GSV** | | | | | **OLIVER** | | | | | |
| --- | --- | --- | --- | --- | --- | --- | --- | --- | --- | --- |
| **GSV ID** | **ID** | **SD** | **TPM AVRG** | **CV** | **ResultFile** | **OLIVER cv** | **ResultFile** | **geomean expratio cv OLIVER method 10** | **ResultFile** | **avgexpratio avgcv OLIVER method 14** |
| 1 | gene377 | 0.110245 | 9.723479 | 0.011338 | gene49 | 0.072039715 | gene49 | 0.31417451 | gene290 | 0.793945955 |
| 2 | gene290 | 0.121321 | 9.339871 | 0.01299 | gene377 | 0.077310402 | gene377 | 0.33298232 | gene55 | 0.7962386 |
| 3 | gene222 | 0.197401 | 9.733793 | 0.02028 | gene290 | 0.084592793 | gene290 | 0.34160419 | gene49 | 0.805766566 |
| 4 | gene155 | 0.194373 | 9.529592 | 0.020397 | gene69 | 0.092272792 | gene50 | 0.36414906 | gene377 | 0.816994433 |
| 5 | gene325 | 0.198272 | 9.465065 | 0.020948 | gene50 | 0.093969762 | gene69 | 0.36678234 | gene286 | 0.849679025 |
| 6 | gene286 | 0.203005 | 9.439783 | 0.021505 | gene88 | 0.094540069 | gene34 | 0.37005926 | gene62 | 0.855130919 |
| 7 | gene133 | 0.251593 | 9.261978 | 0.027164 | gene34 | 0.097975811 | gene81 | 0.37152753 | gene43 | 0.867789381 |
| 8 | gene342 | 0.26335 | 9.682192 | 0.027199 | gene81 | 0.099273382 | gene88 | 0.37557419 | gene58 | 0.871200647 |
| 9 | gene273 | 0.269506 | 9.678665 | 0.027845 | gene5 | 0.100691276 | gene65 | 0.3853843 | gene5 | 0.872066147 |
| 10 | gene480 | 0.272768 | 9.662264 | 0.02823 | gene39 | 0.103061366 | gene39 | 0.38661365 | gene65 | 0.876484488 |
| 11 | gene340 | 0.265637 | 9.334172 | 0.028458 | gene30 | 0.10425852 | gene30 | 0.39151457 | gene88 | 0.876910968 |
| 12 | gene378 | 0.281031 | 9.591588 | 0.0293 | gene65 | 0.104277186 | gene5 | 0.39515376 | gene18 | 0.877284842 |
| 13 | gene409 | 0.276717 | 9.34019 | 0.029626 | gene97 | 0.106799586 | gene55 | 0.39519505 | gene155 | 0.877293939 |
| 14 | gene453 | 0.285897 | 9.6226 | 0.029711 | gene72 | 0.108709629 | gene97 | 0.39677979 | gene81 | 0.877752648 |
| 15 | gene431 | 0.283752 | 9.401054 | 0.030183 | gene55 | 0.109153461 | gene71 | 0.39845149 | gene99 | 0.879467591 |
| 16 | gene402 | 0.278525 | 9.226712 | 0.030187 | gene14 | 0.10958466 | gene4 | 0.40214627 | gene60 | 0.880768098 |
| 17 | gene300 | 0.283573 | 9.378202 | 0.030237 | gene4 | 0.110504181 | gene21 | 0.40218131 | gene27 | 0.881165452 |
| 18 | gene360 | 0.299527 | 9.536431 | 0.031409 | gene21 | 0.112161477 | gene72 | 0.40465776 | gene69 | 0.885551289 |
| 19 | gene264 | 0.306551 | 9.50114 | 0.032265 | gene71 | 0.112387151 | gene58 | 0.40479102 | gene39 | 0.886424362 |
| 20 | gene101 | 0.289697 | 8.926785 | 0.032453 | gene26 | 0.112591751 | gene0 | 0.40753389 | gene51 | 0.88650986 |
| 21 | gene438 | 0.316337 | 9.679207 | 0.032682 | gene0 | 0.114821464 | gene26 | 0.41472995 | gene21 | 0.890896928 |
| 22 | gene451 | 0.308391 | 9.266564 | 0.03328 | gene58 | 0.115358827 | gene14 | 0.41752775 | gene96 | 0.891294697 |
| 23 | gene489 | 0.326176 | 9.595713 | 0.033992 | gene96 | 0.11566427 | gene86 | 0.41896997 | gene50 | 0.894600778 |
| 24 | gene404 | 0.292991 | 8.605131 | 0.034048 | gene45 | 0.119657509 | gene45 | 0.41964057 | gene26 | 0.895943348 |
| 25 | gene405 | 0.337236 | 9.51922 | 0.035427 | gene98 | 0.121044251 | gene87 | 0.42065432 | gene86 | 0.89725746 |
| 26 | gene329 | 0.339012 | 9.519928 | 0.035611 | gene22 | 0.122169946 | gene98 | 0.42779031 | gene222 | 0.899485054 |
| 27 | gene441 | 0.346043 | 9.704271 | 0.035659 | gene85 | 0.12416787 | gene96 | 0.42993508 | gene0 | 0.901639186 |
| 28 | gene237 | 0.336744 | 9.191205 | 0.036638 | gene87 | 0.126879443 | gene27 | 0.4308766 | gene47 | 0.902099929 |
| 29 | gene380 | 0.351458 | 9.548838 | 0.036806 | gene27 | 0.127739592 | gene56 | 0.43557585 | gene61 | 0.902471509 |
| 30 | gene482 | 0.339798 | 8.879414 | 0.038268 | gene86 | 0.127991713 | gene43 | 0.43847882 | gene23 | 0.905916384 |
| 31 | gene285 | 0.360836 | 9.367363 | 0.038521 | gene222 | 0.129709827 | gene22 | 0.43999875 | gene95 | 0.907171369 |
| 32 | gene223 | 0.368917 | 9.423726 | 0.039148 | gene12 | 0.130113969 | gene73 | 0.44193947 | gene89 | 0.91050755 |
| 33 | gene411 | 0.371132 | 9.345639 | 0.039712 | gene43 | 0.13037975 | gene85 | 0.44309628 | gene4 | 0.912115287 |
| 34 | gene319 | 0.386251 | 9.432352 | 0.04095 | gene38 | 0.132011703 | gene325 | 0.44709958 | gene34 | 0.913265736 |
| 35 | gene370 | 0.370306 | 8.861963 | 0.041786 | gene155 | 0.134013082 | gene222 | 0.44876807 | gene9 | 0.914212663 |
| 36 | gene383 | 0.394944 | 9.429722 | 0.041883 | gene325 | 0.134598401 | gene286 | 0.44896724 | gene71 | 0.918427084 |
| 37 | gene490 | 0.390639 | 9.284677 | 0.042073 | gene56 | 0.134882839 | gene24 | 0.4512028 | gene56 | 0.921657929 |
| 38 | gene282 | 0.394126 | 9.349026 | 0.042157 | gene35 | 0.135538533 | gene155 | 0.45120627 | gene57 | 0.921906678 |
| 39 | gene363 | 0.394371 | 9.295877 | 0.042424 | gene37 | 0.13565563 | gene25 | 0.45188597 | gene52 | 0.924426051 |
| 40 | gene140 | 0.38969 | 9.024626 | 0.043181 | gene25 | 0.136017859 | gene37 | 0.45259207 | gene87 | 0.924878738 |
| 41 | gene491 | 0.39045 | 9.018882 | 0.043292 | gene24 | 0.136114925 | gene12 | 0.45367608 | gene17 | 0.924941295 |
| 42 | gene463 | 0.387535 | 8.929904 | 0.043397 | gene40 | 0.13785083 | gene35 | 0.4549341 | gene14 | 0.926610858 |
| 43 | gene381 | 0.405554 | 9.317427 | 0.043526 | gene51 | 0.138642763 | gene60 | 0.45498998 | gene85 | 0.927674962 |
| 44 | gene462 | 0.406756 | 9.31912 | 0.043647 | gene17 | 0.14019978 | gene19 | 0.45550604 | gene325 | 0.932350047 |
| 45 | gene113 | 0.411164 | 9.377169 | 0.043847 | gene19 | 0.140382655 | gene51 | 0.45624781 | gene37 | 0.934317011 |
| 46 | gene368 | 0.385256 | 8.713823 | 0.044212 | gene92 | 0.14055934 | gene95 | 0.45699823 | gene98 | 0.934946241 |
| 47 | gene464 | 0.409296 | 9.234585 | 0.044322 | gene53 | 0.140982506 | gene52 | 0.45794271 | gene2 | 0.936159142 |
| 48 | gene142 | 0.426945 | 9.629661 | 0.044336 | gene73 | 0.141509716 | gene92 | 0.45894919 | gene7 | 0.938562877 |
| 49 | gene121 | 0.405707 | 9.072561 | 0.044718 | gene60 | 0.142243167 | gene76 | 0.45968521 | gene24 | 0.938609705 |
| 50 | gene219 | 0.41713 | 9.298939 | 0.044858 | gene52 | 0.142248074 | gene23 | 0.46105751 | gene42 | 0.94148541 |
| 51 | gene474 | 0.419279 | 9.281502 | 0.045174 | gene95 | 0.142981302 | gene82 | 0.46133052 | gene76 | 0.942709803 |
| 52 | gene160 | 0.442174 | 9.525586 | 0.04642 | gene286 | 0.143433988 | gene38 | 0.46168888 | gene45 | 0.94510598 |
| 53 | gene190 | 0.413599 | 8.787055 | 0.047069 | gene10 | 0.143746904 | gene99 | 0.46575832 | gene90 | 0.945301157 |
| 54 | gene115 | 0.41967 | 8.908811 | 0.047107 | gene23 | 0.144383287 | gene18 | 0.46749931 | gene93 | 0.945579063 |
| 55 | gene395 | 0.433483 | 9.105883 | 0.047605 | gene76 | 0.14482552 | gene40 | 0.46794167 | gene97 | 0.947858107 |
| 56 | gene298 | 0.439013 | 9.205788 | 0.047689 | gene82 | 0.145098223 | gene10 | 0.46849754 | gene92 | 0.948080842 |
| 57 | gene403 | 0.45438 | 9.440651 | 0.04813 | gene47 | 0.147214147 | gene2 | 0.46871041 | gene36 | 0.950345434 |
| 58 | gene269 | 0.429285 | 8.891437 | 0.048281 | gene89 | 0.148121817 | gene62 | 0.46913475 | gene74 | 0.950716643 |
| 59 | gene316 | 0.456884 | 9.369227 | 0.048764 | gene32 | 0.148137287 | gene17 | 0.46970009 | gene94 | 0.951865559 |
| 60 | gene265 | 0.452076 | 9.265011 | 0.048794 | gene99 | 0.148162112 | gene53 | 0.47167827 | gene1 | 0.952304113 |
| 61 | gene281 | 0.450025 | 9.218313 | 0.048819 | gene9 | 0.148562042 | gene89 | 0.47304515 | gene11 | 0.953421913 |
| 62 | gene385 | 0.457144 | 9.347046 | 0.048908 | gene94 | 0.14974238 | gene61 | 0.47443473 | gene28 | 0.953943269 |
| 63 | gene112 | 0.451796 | 9.166367 | 0.049288 | gene77 | 0.150300317 | gene83 | 0.47516933 | gene22 | 0.954231174 |
| 64 | gene354 | 0.46519 | 9.415508 | 0.049407 | gene18 | 0.150308727 | gene75 | 0.47772774 | gene82 | 0.955889309 |
| 65 | gene162 | 0.45649 | 9.238509 | 0.049412 | gene80 | 0.151118192 | gene47 | 0.47806551 | gene10 | 0.957363832 |
| 66 | gene312 | 0.448839 | 9.072878 | 0.04947 | gene29 | 0.151936367 | gene7 | 0.48127221 | gene73 | 0.957444654 |
| 67 | gene198 | 0.471693 | 9.427414 | 0.050034 | gene62 | 0.152749847 | gene67 | 0.48282951 | gene48 | 0.959512588 |
| 68 | gene186 | 0.458638 | 9.146273 | 0.050145 | gene83 | 0.152775999 | gene9 | 0.48366329 | gene83 | 0.9604332 |
| 69 | gene217 | 0.415232 | 8.112677 | 0.051183 | gene68 | 0.152815936 | gene84 | 0.48376038 | gene40 | 0.960548447 |
| 70 | gene371 | 0.467681 | 9.134597 | 0.051199 | gene59 | 0.154177149 | gene42 | 0.48501816 | gene29 | 0.960826498 |
| 71 | gene154 | 0.477569 | 9.294193 | 0.051384 | gene2 | 0.154338187 | gene77 | 0.48518359 | gene44 | 0.961297723 |
| 72 | gene261 | 0.456646 | 8.866917 | 0.0515 | gene33 | 0.154585583 | gene91 | 0.48559767 | gene13 | 0.962538778 |
| 73 | gene348 | 0.455711 | 8.816046 | 0.051691 | gene54 | 0.154735957 | gene80 | 0.48650717 | gene38 | 0.962608144 |
| 74 | gene130 | 0.444603 | 8.569519 | 0.051882 | gene61 | 0.155838551 | gene78 | 0.48805128 | gene30 | 0.964864139 |
| 75 | gene196 | 0.47718 | 9.182479 | 0.051966 | gene75 | 0.156860112 | gene94 | 0.48837707 | gene35 | 0.965427955 |
| 76 | gene224 | 0.448343 | 8.603868 | 0.052109 | gene78 | 0.15981751 | gene93 | 0.48884559 | gene25 | 0.966095173 |
| 77 | gene355 | 0.493928 | 9.415029 | 0.052462 | gene48 | 0.160456475 | gene46 | 0.49102107 | gene64 | 0.968595176 |
| 78 | gene174 | 0.487837 | 9.276248 | 0.05259 | gene93 | 0.160729908 | gene68 | 0.4918095 | gene480 | 0.968691417 |
| 79 | gene345 | 0.500847 | 9.52274 | 0.052595 | gene7 | 0.161460632 | gene59 | 0.49217234 | gene75 | 0.973316609 |
| 80 | gene478 | 0.46561 | 8.70687 | 0.053476 | gene46 | 0.161915843 | gene11 | 0.49223204 | gene31 | 0.976471895 |
| 81 | gene270 | 0.458079 | 8.532964 | 0.053683 | gene16 | 0.162496703 | gene29 | 0.49469262 | gene8 | 0.976773221 |
| 82 | gene483 | 0.520701 | 9.60062 | 0.054236 | gene42 | 0.163744405 | gene20 | 0.49471088 | gene67 | 0.980024768 |
| 83 | gene461 | 0.495902 | 9.113772 | 0.054412 | gene41 | 0.165148031 | gene8 | 0.49534278 | gene68 | 0.980383219 |
| 84 | gene400 | 0.504218 | 9.246303 | 0.054532 | gene67 | 0.165263131 | gene133 | 0.49616126 | gene53 | 0.981350618 |
| 85 | gene485 | 0.515413 | 9.416684 | 0.054734 | gene11 | 0.165848503 | gene70 | 0.49649558 | gene54 | 0.98265464 |
| 86 | gene236 | 0.514288 | 9.341388 | 0.055055 | gene91 | 0.166971407 | gene32 | 0.4965896 | gene72 | 0.983277249 |
| 87 | gene199 | 0.49374 | 8.961057 | 0.055098 | gene84 | 0.167080912 | gene74 | 0.49661986 | gene16 | 0.984535262 |
| 88 | gene221 | 0.499155 | 8.961649 | 0.055699 | gene8 | 0.167156937 | gene44 | 0.49765662 | gene91 | 0.984586421 |
| 89 | gene443 | 0.502348 | 8.999475 | 0.05582 | gene74 | 0.167516404 | gene64 | 0.49771673 | gene19 | 0.985530593 |
| 90 | gene105 | 0.518364 | 9.275857 | 0.055883 | gene3 | 0.169824301 | gene36 | 0.49789598 | gene78 | 0.987370612 |
| 91 | gene327 | 0.493341 | 8.752264 | 0.056367 | gene70 | 0.170059682 | gene13 | 0.4997029 | gene3 | 0.99095125 |
| 92 | gene148 | 0.507125 | 8.939788 | 0.056727 | gene6 | 0.170936416 | gene54 | 0.50031221 | gene59 | 0.991908069 |
| 93 | gene364 | 0.52249 | 9.201874 | 0.056781 | gene28 | 0.170938084 | gene48 | 0.50162334 | gene340 | 0.995341726 |
| 94 | gene126 | 0.529421 | 9.254366 | 0.057208 | gene13 | 0.171206106 | gene33 | 0.50427646 | gene6 | 0.995827862 |
| 95 | gene233 | 0.519669 | 9.06728 | 0.057313 | gene44 | 0.171428007 | gene79 | 0.50482104 | gene378 | 0.997322626 |
| 96 | gene234 | 0.534136 | 9.311019 | 0.057366 | gene63 | 0.17153193 | gene16 | 0.5062849 | gene70 | 0.997487297 |
| 97 | gene486 | 0.556614 | 9.661029 | 0.057614 | gene64 | 0.171583395 | gene6 | 0.50755921 | gene431 | 1.001397813 |
| 98 | gene205 | 0.535337 | 9.221263 | 0.058055 | gene20 | 0.172681171 | gene41 | 0.50803095 | gene77 | 1.002732245 |
| 99 | gene437 | 0.525694 | 9.026347 | 0.05824 | gene133 | 0.174499233 | gene57 | 0.50937107 | gene20 | 1.003671699 |
| 100 | gene499 | 0.537983 | 9.201778 | 0.058465 | gene342 | 0.174962688 | gene66 | 0.51109205 | gene409 | 1.004381904 |
| 101 | gene331 | 0.551006 | 9.416067 | 0.058518 | gene66 | 0.175185664 | gene28 | 0.51146769 | gene453 | 1.006185839 |
| 102 | gene358 | 0.548197 | 9.351661 | 0.05862 | gene15 | 0.175300391 | gene15 | 0.51190269 | gene101 | 1.006589455 |
| 103 | gene188 | 0.548555 | 9.320316 | 0.058856 | gene79 | 0.176907741 | gene342 | 0.51493263 | gene79 | 1.009435144 |
| 104 | gene391 | 0.538661 | 9.109576 | 0.059131 | gene273 | 0.177553781 | gene63 | 0.51733688 | gene84 | 1.009976105 |
| 105 | gene102 | 0.518276 | 8.758253 | 0.059176 | gene378 | 0.17862346 | gene3 | 0.5197278 | gene402 | 1.010216204 |
| 106 | gene317 | 0.535993 | 8.992143 | 0.059607 | gene57 | 0.178773518 | gene1 | 0.52017691 | gene15 | 1.014168092 |
| 107 | gene458 | 0.553143 | 9.231845 | 0.059917 | gene36 | 0.179954405 | gene90 | 0.52543736 | gene66 | 1.014271104 |
| 108 | gene191 | 0.572379 | 9.542553 | 0.059982 | gene31 | 0.180587418 | gene273 | 0.5261293 | gene32 | 1.016066676 |
| 109 | gene187 | 0.554849 | 9.234199 | 0.060086 | gene90 | 0.181883015 | gene31 | 0.52699233 | gene46 | 1.016215615 |
| 110 | gene129 | 0.558987 | 9.272431 | 0.060285 | gene340 | 0.182762622 | gene480 | 0.52961172 | gene12 | 1.017556779 |
| 111 | gene469 | 0.553644 | 9.122029 | 0.060693 | gene453 | 0.183176518 | gene409 | 0.53102822 | gene41 | 1.018499533 |
| 112 | gene314 | 0.565323 | 9.311413 | 0.060713 | gene438 | 0.186659659 | gene453 | 0.53175614 | gene342 | 1.020443158 |
| 113 | gene110 | 0.542654 | 8.916722 | 0.060858 | gene480 | 0.187862722 | gene340 | 0.53326904 | gene80 | 1.020698252 |
| 114 | gene481 | 0.552099 | 9.012274 | 0.061261 | gene402 | 0.189667715 | gene378 | 0.53470474 | gene133 | 1.020781847 |
| 115 | gene225 | 0.580758 | 9.422626 | 0.061634 | gene101 | 0.194167816 | gene402 | 0.54619663 | gene451 | 1.021270965 |
| 116 | gene415 | 0.553646 | 8.964641 | 0.061759 | gene300 | 0.195066721 | gene431 | 0.54784434 | gene404 | 1.027809254 |
| 117 | gene108 | 0.567888 | 9.16294 | 0.061977 | gene1 | 0.196140462 | gene300 | 0.55092368 | gene237 | 1.02814186 |
| 118 | gene367 | 0.58668 | 9.41637 | 0.062304 | gene409 | 0.200642477 | gene101 | 0.55296074 | gene360 | 1.029226562 |
| 119 | gene433 | 0.577804 | 9.271529 | 0.06232 | gene404 | 0.203604703 | gene438 | 0.55677738 | gene63 | 1.036384355 |
| 120 | gene295 | 0.568381 | 9.026776 | 0.062966 | gene451 | 0.203752009 | gene404 | 0.56164379 | gene33 | 1.042352432 |
| 121 | gene137 | 0.560309 | 8.851647 | 0.0633 | gene360 | 0.205182587 | gene264 | 0.56508619 | gene300 | 1.042643185 |
| 122 | gene172 | 0.570857 | 9.017421 | 0.063306 | gene441 | 0.210116719 | gene360 | 0.56690415 | gene223 | 1.048377075 |
| 123 | gene226 | 0.575414 | 8.984564 | 0.064045 | gene264 | 0.212489647 | gene489 | 0.57325231 | gene380 | 1.052519972 |
| 124 | gene117 | 0.590426 | 9.164041 | 0.064429 | gene405 | 0.213227994 | gene441 | 0.57728683 | gene264 | 1.064075905 |
| 125 | gene302 | 0.577137 | 8.91109 | 0.064766 | gene489 | 0.214790365 | gene405 | 0.57825026 | gene273 | 1.066763689 |
| 126 | gene220 | 0.60698 | 9.368835 | 0.064787 | gene431 | 0.217532777 | gene451 | 0.57996618 | gene395 | 1.082085433 |
| 127 | gene454 | 0.604395 | 9.325822 | 0.064809 | gene380 | 0.219599644 | gene482 | 0.5929863 | gene285 | 1.08317508 |
| 128 | gene318 | 0.587357 | 9.0585 | 0.06484 | gene482 | 0.22357479 | gene237 | 0.59477907 | gene319 | 1.086101048 |
| 129 | gene347 | 0.579235 | 8.910055 | 0.065009 | gene237 | 0.226844427 | gene140 | 0.60571174 | gene490 | 1.08776056 |
| 130 | gene139 | 0.599801 | 9.192913 | 0.065246 | gene329 | 0.227254557 | gene380 | 0.60600234 | gene489 | 1.090186784 |
| 131 | gene287 | 0.604772 | 9.228274 | 0.065535 | gene219 | 0.240238004 | gene329 | 0.60800971 | gene329 | 1.090778933 |
| 132 | gene386 | 0.613988 | 9.337544 | 0.065755 | gene140 | 0.241179276 | gene223 | 0.61310336 | gene438 | 1.101941693 |
| 133 | gene392 | 0.585524 | 8.860144 | 0.066085 | gene370 | 0.244523868 | gene370 | 0.61566377 | gene405 | 1.105935545 |
| 134 | gene422 | 0.618954 | 9.363546 | 0.066103 | gene411 | 0.244903673 | gene285 | 0.61827885 | gene462 | 1.11647497 |
| 135 | gene194 | 0.60239 | 9.040156 | 0.066635 | gene142 | 0.246940105 | gene363 | 0.62985055 | gene370 | 1.117137388 |
| 136 | gene389 | 0.598435 | 8.935964 | 0.066969 | gene474 | 0.247607623 | gene462 | 0.63226079 | gene160 | 1.123966521 |
| 137 | gene150 | 0.608347 | 9.067104 | 0.067094 | gene223 | 0.251949476 | gene411 | 0.63529753 | gene482 | 1.124014559 |
| 138 | gene124 | 0.625089 | 9.203277 | 0.06792 | gene285 | 0.253396557 | gene368 | 0.63606633 | gene113 | 1.129506658 |
| 139 | gene169 | 0.609418 | 8.957367 | 0.068035 | gene121 | 0.25500676 | gene282 | 0.63657163 | gene282 | 1.132145102 |
| 140 | gene211 | 0.635792 | 9.259794 | 0.068662 | gene368 | 0.255031909 | gene474 | 0.63677006 | gene121 | 1.133564969 |
| 141 | gene430 | 0.652274 | 9.402468 | 0.069373 | gene113 | 0.255693783 | gene121 | 0.63745008 | gene491 | 1.13479076 |
| 142 | gene180 | 0.627148 | 9.006883 | 0.06963 | gene363 | 0.256310235 | gene491 | 0.64049829 | gene464 | 1.136244179 |
| 143 | gene207 | 0.647564 | 9.295203 | 0.069666 | gene462 | 0.261167595 | gene381 | 0.64199292 | gene269 | 1.136829929 |
| 144 | gene208 | 0.631693 | 9.059422 | 0.069728 | gene491 | 0.261996981 | gene490 | 0.64222553 | gene368 | 1.138920018 |
| 145 | gene445 | 0.63666 | 9.110655 | 0.069881 | gene319 | 0.263124579 | gene319 | 0.6440683 | gene217 | 1.145030672 |
| 146 | gene460 | 0.656552 | 9.351252 | 0.07021 | gene490 | 0.263702457 | gene383 | 0.6443762 | gene463 | 1.145080863 |
| 147 | gene255 | 0.615446 | 8.761603 | 0.070244 | gene464 | 0.27297408 | gene113 | 0.64484913 | gene162 | 1.14713484 |
| 148 | gene123 | 0.653465 | 9.26713 | 0.070514 | gene160 | 0.274157567 | gene463 | 0.66078773 | gene383 | 1.150242952 |
| 149 | gene288 | 0.627881 | 8.883693 | 0.070678 | gene383 | 0.275312536 | gene217 | 0.66254864 | gene298 | 1.152518111 |
| 150 | gene229 | 0.65784 | 9.274891 | 0.070927 | gene261 | 0.276680703 | gene142 | 0.66332986 | gene403 | 1.15416681 |
| 151 | gene487 | 0.636299 | 8.970894 | 0.070929 | gene381 | 0.277997578 | gene219 | 0.66475755 | gene385 | 1.157497629 |
| 152 | gene232 | 0.651344 | 9.148612 | 0.071196 | gene282 | 0.279197316 | gene190 | 0.66481299 | gene312 | 1.163955667 |
| 153 | gene320 | 0.638596 | 8.968043 | 0.071208 | gene395 | 0.280534718 | gene464 | 0.67072821 | gene219 | 1.164660442 |
| 154 | gene369 | 0.631654 | 8.867957 | 0.071229 | gene354 | 0.281923335 | gene261 | 0.67087384 | gene381 | 1.165659138 |
| 155 | gene305 | 0.650116 | 9.040757 | 0.071909 | gene196 | 0.283805757 | gene395 | 0.67209474 | gene363 | 1.168984249 |
| 156 | gene338 | 0.674319 | 9.375862 | 0.071921 | gene486 | 0.285318281 | gene403 | 0.67546433 | gene411 | 1.169644746 |
| 157 | gene387 | 0.633306 | 8.802425 | 0.071947 | gene115 | 0.286052037 | gene160 | 0.67679352 | gene140 | 1.170745462 |
| 158 | gene277 | 0.610383 | 8.472173 | 0.072046 | gene483 | 0.287186989 | gene371 | 0.68712916 | gene190 | 1.172230024 |
| 159 | gene146 | 0.625287 | 8.65019 | 0.072286 | gene217 | 0.2872596 | gene385 | 0.6892677 | gene441 | 1.174683868 |
| 160 | gene455 | 0.646991 | 8.942941 | 0.072347 | gene403 | 0.288509141 | gene115 | 0.68954536 | gene115 | 1.174729521 |
| 161 | gene120 | 0.665042 | 9.155697 | 0.072637 | gene345 | 0.290474221 | gene281 | 0.68957957 | gene142 | 1.174997209 |
| 162 | gene176 | 0.675221 | 9.277494 | 0.072781 | gene371 | 0.292142502 | gene269 | 0.68982226 | gene196 | 1.178205403 |
| 163 | gene410 | 0.682493 | 9.347383 | 0.073014 | gene236 | 0.293767896 | gene348 | 0.69332804 | gene461 | 1.18007313 |
| 164 | gene227 | 0.683754 | 9.352516 | 0.073109 | gene105 | 0.29518513 | gene224 | 0.69587833 | gene186 | 1.185729076 |
| 165 | gene128 | 0.672554 | 9.181155 | 0.073254 | gene191 | 0.29713221 | gene130 | 0.69605823 | gene130 | 1.191162015 |
| 166 | gene374 | 0.680313 | 9.259082 | 0.073475 | gene130 | 0.302366285 | gene162 | 0.69641585 | gene198 | 1.193070981 |
| 167 | gene424 | 0.647341 | 8.800349 | 0.073559 | gene367 | 0.302772801 | gene196 | 0.69646473 | gene281 | 1.19600211 |
| 168 | gene280 | 0.664414 | 9.027742 | 0.073597 | gene190 | 0.302859683 | gene198 | 0.69889864 | gene199 | 1.197757843 |
| 169 | gene303 | 0.649247 | 8.805604 | 0.073731 | gene186 | 0.307570856 | gene112 | 0.70045522 | gene478 | 1.202748796 |
| 170 | gene412 | 0.6363 | 8.56342 | 0.074304 | gene269 | 0.307621151 | gene270 | 0.70050703 | gene265 | 1.202870652 |
| 171 | gene289 | 0.65064 | 8.749356 | 0.074364 | gene348 | 0.308936084 | gene354 | 0.70160303 | gene174 | 1.208909251 |
| 172 | gene212 | 0.681043 | 9.098806 | 0.07485 | gene385 | 0.30984492 | gene298 | 0.70246504 | gene154 | 1.209157813 |
| 173 | gene423 | 0.677218 | 9.019222 | 0.075086 | gene198 | 0.310478143 | gene316 | 0.7033482 | gene348 | 1.215504326 |
| 174 | gene323 | 0.684068 | 9.089716 | 0.075257 | gene331 | 0.313008547 | gene331 | 0.7089583 | gene129 | 1.21567525 |
| 175 | gene135 | 0.677319 | 8.981808 | 0.07541 | gene463 | 0.31313479 | gene312 | 0.71364168 | gene224 | 1.220463294 |
| 176 | gene165 | 0.676716 | 8.967152 | 0.075466 | gene148 | 0.314911174 | gene126 | 0.71643653 | gene316 | 1.229709058 |
| 177 | gene439 | 0.676875 | 8.963107 | 0.075518 | gene316 | 0.315427643 | gene154 | 0.71845599 | gene112 | 1.234124073 |
| 178 | gene125 | 0.705699 | 9.328961 | 0.075646 | gene112 | 0.316165245 | gene355 | 0.71852727 | gene270 | 1.23600137 |
| 179 | gene127 | 0.670492 | 8.861495 | 0.075663 | gene478 | 0.317602344 | gene400 | 0.72137649 | gene205 | 1.236499925 |
| 180 | gene470 | 0.684039 | 9.006602 | 0.075949 | gene314 | 0.320786543 | gene191 | 0.7217252 | gene105 | 1.236762693 |
| 181 | gene267 | 0.69909 | 9.181825 | 0.076138 | gene355 | 0.32129314 | gene186 | 0.72230191 | gene483 | 1.236914991 |
| 182 | gene171 | 0.695997 | 9.109995 | 0.076399 | gene281 | 0.321617667 | gene234 | 0.72318016 | gene400 | 1.239708105 |
| 183 | gene231 | 0.655567 | 8.570441 | 0.076492 | gene221 | 0.323021918 | gene499 | 0.72321083 | gene225 | 1.242246441 |
| 184 | gene153 | 0.682486 | 8.910924 | 0.07659 | gene265 | 0.323554674 | gene486 | 0.72372463 | gene355 | 1.242586356 |
| 185 | gene214 | 0.680168 | 8.874836 | 0.07664 | gene312 | 0.324997751 | gene367 | 0.72443233 | gene261 | 1.247027259 |
| 186 | gene311 | 0.671873 | 8.751224 | 0.076775 | gene162 | 0.326698638 | gene483 | 0.7265237 | gene221 | 1.247292392 |
| 187 | gene147 | 0.651146 | 8.472383 | 0.076855 | gene270 | 0.327241304 | gene478 | 0.72860975 | gene317 | 1.248412429 |
| 188 | gene200 | 0.699617 | 9.074383 | 0.077098 | gene154 | 0.327353742 | gene345 | 0.72992998 | gene187 | 1.252962828 |
| 189 | gene442 | 0.634968 | 8.209995 | 0.077341 | gene364 | 0.327898507 | gene221 | 0.7315802 | gene458 | 1.256375606 |
| 190 | gene432 | 0.695441 | 8.944093 | 0.077754 | gene298 | 0.328279 | gene174 | 0.73311737 | gene302 | 1.259078678 |
| 191 | gene251 | 0.671764 | 8.628962 | 0.07785 | gene174 | 0.328701734 | gene461 | 0.73546784 | gene354 | 1.259540472 |
| 192 | gene185 | 0.6672 | 8.552747 | 0.07801 | gene233 | 0.329080421 | gene233 | 0.73627062 | gene117 | 1.261653412 |
| 193 | gene163 | 0.661393 | 8.442091 | 0.078345 | gene386 | 0.329462533 | gene265 | 0.73682665 | gene314 | 1.263159132 |
| 194 | gene106 | 0.667902 | 8.524511 | 0.078351 | gene485 | 0.330662955 | gene236 | 0.73701079 | gene443 | 1.264514449 |
| 195 | gene246 | 0.69626 | 8.867978 | 0.078514 | gene234 | 0.332700096 | gene317 | 0.74009215 | gene371 | 1.26892407 |
| 196 | gene337 | 0.7064 | 8.952979 | 0.078901 | gene499 | 0.333326014 | gene485 | 0.74086209 | gene391 | 1.26965057 |
| 197 | gene493 | 0.73126 | 9.24028 | 0.079138 | gene400 | 0.334031235 | gene148 | 0.74133024 | gene345 | 1.276226653 |
| 198 | gene299 | 0.703906 | 8.869857 | 0.079359 | gene224 | 0.336381768 | gene199 | 0.74282518 | gene188 | 1.276479528 |
| 199 | gene353 | 0.680958 | 8.53774 | 0.079759 | gene389 | 0.336768659 | gene481 | 0.74380471 | gene148 | 1.277524112 |
| 200 | gene343 | 0.731406 | 9.114233 | 0.080249 | gene126 | 0.337296134 | gene188 | 0.74878518 | gene485 | 1.278694099 |
| 201 | gene206 | 0.689697 | 8.557122 | 0.080599 | gene317 | 0.338327845 | gene105 | 0.75052865 | gene126 | 1.279453412 |
| 202 | gene322 | 0.727605 | 9.021938 | 0.080648 | gene461 | 0.338883908 | gene102 | 0.75235671 | gene474 | 1.279931569 |
| 203 | gene193 | 0.712018 | 8.807526 | 0.080842 | gene207 | 0.340441833 | gene205 | 0.7528931 | gene415 | 1.280124639 |
| 204 | gene399 | 0.753953 | 9.321727 | 0.080881 | gene129 | 0.343961624 | gene169 | 0.75301167 | gene364 | 1.280600478 |
| 205 | gene435 | 0.696561 | 8.606158 | 0.080938 | gene433 | 0.345505315 | gene386 | 0.75307027 | gene236 | 1.2841998 |
| 206 | gene447 | 0.71803 | 8.870205 | 0.080949 | gene188 | 0.345744634 | gene364 | 0.75690425 | gene437 | 1.287017737 |
| 207 | gene184 | 0.724892 | 8.944211 | 0.081046 | gene227 | 0.345761477 | gene389 | 0.75898892 | gene327 | 1.287240349 |
| 208 | gene149 | 0.689182 | 8.49845 | 0.081095 | gene358 | 0.346318906 | gene358 | 0.76177571 | gene234 | 1.287558836 |
| 209 | gene204 | 0.730021 | 8.975156 | 0.081338 | gene225 | 0.346887292 | gene433 | 0.76398334 | gene481 | 1.290765471 |
| 210 | gene315 | 0.73703 | 9.061328 | 0.081338 | gene481 | 0.34691354 | gene327 | 0.76432296 | gene137 | 1.29377928 |
| 211 | gene245 | 0.76012 | 9.288598 | 0.081834 | gene430 | 0.349634043 | gene129 | 0.76475157 | gene486 | 1.29386156 |
| 212 | gene103 | 0.75974 | 9.262324 | 0.082025 | gene458 | 0.349853547 | gene225 | 0.76608362 | gene102 | 1.294718852 |
| 213 | gene138 | 0.756179 | 9.183371 | 0.082342 | gene347 | 0.353374639 | gene207 | 0.7675128 | gene499 | 1.30208996 |
| 214 | gene495 | 0.736628 | 8.943049 | 0.082369 | gene229 | 0.358663975 | gene314 | 0.77065388 | gene233 | 1.305881441 |
| 215 | gene398 | 0.75014 | 9.10304 | 0.082405 | gene169 | 0.359767952 | gene347 | 0.77112564 | gene191 | 1.309456943 |
| 216 | gene216 | 0.751898 | 9.069758 | 0.082902 | gene108 | 0.360324738 | gene220 | 0.77140936 | gene108 | 1.309868261 |
| 217 | gene143 | 0.755362 | 9.106157 | 0.082951 | gene397 | 0.361941177 | gene108 | 0.77421059 | gene226 | 1.310548345 |
| 218 | gene436 | 0.758445 | 9.119259 | 0.08317 | gene399 | 0.36224913 | gene391 | 0.77430546 | gene331 | 1.313813776 |
| 219 | gene274 | 0.750928 | 9.015648 | 0.083292 | gene398 | 0.363564879 | gene437 | 0.77736971 | gene469 | 1.314042148 |
| 220 | gene276 | 0.726929 | 8.723702 | 0.083328 | gene410 | 0.364284218 | gene194 | 0.78017315 | gene110 | 1.314774319 |
| 221 | gene448 | 0.734614 | 8.814719 | 0.08334 | gene138 | 0.365320736 | gene117 | 0.78020218 | gene460 | 1.317557335 |
| 222 | gene243 | 0.742817 | 8.899465 | 0.083468 | gene437 | 0.365529991 | gene458 | 0.78072207 | gene139 | 1.31802214 |
| 223 | gene418 | 0.736889 | 8.806194 | 0.083678 | gene123 | 0.365861367 | gene415 | 0.78266446 | gene172 | 1.321849422 |
| 224 | gene472 | 0.69036 | 8.246995 | 0.083711 | gene205 | 0.365925277 | gene110 | 0.78373166 | gene358 | 1.328876125 |
| 225 | gene253 | 0.720546 | 8.58195 | 0.083961 | gene460 | 0.367089828 | gene124 | 0.78416353 | gene367 | 1.330342611 |
| 226 | gene352 | 0.732106 | 8.718863 | 0.083968 | gene220 | 0.370205106 | gene422 | 0.78511325 | gene211 | 1.333097184 |
| 227 | gene100 | 0.758188 | 9.023162 | 0.084027 | gene327 | 0.370249996 | gene187 | 0.78631165 | gene435 | 1.333340047 |
| 228 | gene209 | 0.748712 | 8.877473 | 0.084338 | gene187 | 0.371135937 | gene443 | 0.78695676 | gene287 | 1.333994203 |
| 229 | gene114 | 0.736963 | 8.711922 | 0.084593 | gene302 | 0.3719828 | gene146 | 0.78849008 | gene295 | 1.336144226 |
| 230 | gene420 | 0.746231 | 8.79385 | 0.084858 | gene454 | 0.373741718 | gene172 | 0.78879795 | gene288 | 1.338442069 |
| 231 | gene488 | 0.780727 | 9.181589 | 0.085032 | gene216 | 0.37389655 | gene206 | 0.79476673 | gene207 | 1.339540477 |
| 232 | gene230 | 0.775492 | 9.101447 | 0.085205 | gene146 | 0.374303405 | gene123 | 0.79679462 | gene433 | 1.340237229 |
| 233 | gene157 | 0.786686 | 9.231438 | 0.085218 | gene117 | 0.376392187 | gene442 | 0.80120216 | gene320 | 1.34507307 |
| 234 | gene365 | 0.752011 | 8.816006 | 0.085301 | gene338 | 0.378106948 | gene469 | 0.80180265 | gene392 | 1.345299714 |
| 235 | gene425 | 0.771731 | 9.026863 | 0.085493 | gene391 | 0.378325731 | gene445 | 0.80183714 | gene318 | 1.345378709 |
| 236 | gene456 | 0.741539 | 8.66896 | 0.08554 | gene424 | 0.379137396 | gene302 | 0.80225688 | gene220 | 1.345934943 |
| 237 | gene249 | 0.801508 | 9.349018 | 0.085732 | gene199 | 0.381319529 | gene229 | 0.80273476 | gene176 | 1.346650764 |
| 238 | gene183 | 0.795964 | 9.276878 | 0.085801 | gene194 | 0.381426233 | gene305 | 0.80472948 | gene124 | 1.347291682 |
| 239 | gene307 | 0.743445 | 8.649338 | 0.085954 | gene192 | 0.38170727 | gene289 | 0.80545494 | gene369 | 1.35052965 |
| 240 | gene416 | 0.79286 | 9.216943 | 0.086022 | gene161 | 0.3830753 | gene255 | 0.80569567 | gene487 | 1.353585764 |
| 241 | gene471 | 0.792001 | 9.203301 | 0.086056 | gene422 | 0.383643125 | gene424 | 0.81041869 | gene347 | 1.353864792 |
| 242 | gene334 | 0.78046 | 9.047567 | 0.086262 | gene102 | 0.384209592 | gene226 | 0.81145405 | gene194 | 1.355721728 |
| 243 | gene394 | 0.784593 | 9.092433 | 0.086291 | gene124 | 0.38476498 | gene139 | 0.81291085 | gene200 | 1.359613556 |
| 244 | gene475 | 0.757877 | 8.756992 | 0.086545 | gene362 | 0.386674727 | gene430 | 0.81327638 | gene323 | 1.359954245 |
| 245 | gene428 | 0.790084 | 9.126651 | 0.086569 | gene287 | 0.388099232 | gene318 | 0.81461761 | gene454 | 1.361967945 |
| 246 | gene122 | 0.7548 | 8.696976 | 0.086789 | gene128 | 0.388365853 | gene135 | 0.8152589 | gene231 | 1.362070059 |
| 247 | gene379 | 0.740709 | 8.519602 | 0.086942 | gene183 | 0.389479786 | gene374 | 0.8154767 | gene146 | 1.36260988 |
| 248 | gene173 | 0.765581 | 8.791826 | 0.087079 | gene172 | 0.390417871 | gene137 | 0.81635514 | gene150 | 1.364311596 |
| 249 | gene159 | 0.781459 | 8.947635 | 0.087337 | gene249 | 0.390761536 | gene398 | 0.82014422 | gene442 | 1.365499531 |
| 250 | gene417 | 0.757997 | 8.66475 | 0.087481 | gene226 | 0.390823314 | gene454 | 0.82047397 | gene303 | 1.365812661 |
| 251 | gene266 | 0.785558 | 8.979566 | 0.087483 | gene135 | 0.391099536 | gene288 | 0.82200428 | gene180 | 1.366461922 |
| 252 | gene376 | 0.801235 | 9.145889 | 0.087606 | gene469 | 0.391227954 | gene280 | 0.82233885 | gene253 | 1.36703832 |
| 253 | gene476 | 0.757724 | 8.646251 | 0.087636 | gene288 | 0.391276248 | gene287 | 0.82283189 | gene169 | 1.36791418 |
| 254 | gene335 | 0.806653 | 9.203787 | 0.087644 | gene104 | 0.391437526 | gene455 | 0.82340575 | gene455 | 1.374899319 |
| 255 | gene393 | 0.771818 | 8.7946 | 0.08776 | gene374 | 0.396257733 | gene216 | 0.82345039 | gene280 | 1.375512078 |
| 256 | gene178 | 0.815444 | 9.291457 | 0.087763 | gene137 | 0.396312606 | gene138 | 0.82633041 | gene389 | 1.376381889 |
| 257 | gene247 | 0.779962 | 8.863631 | 0.087996 | gene211 | 0.398391013 | gene180 | 0.82638843 | gene218 | 1.376597837 |
| 258 | gene397 | 0.783797 | 8.897419 | 0.088093 | gene289 | 0.398887086 | gene208 | 0.82693037 | gene277 | 1.379890495 |
| 259 | gene167 | 0.759017 | 8.583789 | 0.088424 | gene351 | 0.399341999 | gene128 | 0.82798342 | gene208 | 1.381215597 |
| 260 | gene275 | 0.779271 | 8.781166 | 0.088743 | gene125 | 0.399624756 | gene460 | 0.82805461 | gene424 | 1.381727842 |
| 261 | gene104 | 0.811403 | 9.086022 | 0.089302 | gene415 | 0.399708078 | gene338 | 0.82823787 | gene386 | 1.384106272 |
| 262 | gene341 | 0.786752 | 8.808913 | 0.089313 | gene310 | 0.400371787 | gene295 | 0.82869331 | gene135 | 1.3858938 |
| 263 | gene406 | 0.801763 | 8.968766 | 0.089395 | gene178 | 0.400973379 | gene232 | 0.82875111 | gene204 | 1.388266694 |
| 264 | gene259 | 0.798915 | 8.932766 | 0.089437 | gene423 | 0.402527808 | gene412 | 0.82923447 | gene425 | 1.38847708 |
| 265 | gene484 | 0.817448 | 9.136804 | 0.089468 | gene435 | 0.402887178 | gene423 | 0.83104949 | gene147 | 1.389053126 |
| 266 | gene218 | 0.696961 | 7.756645 | 0.089853 | gene498 | 0.404253216 | gene493 | 0.83142439 | gene439 | 1.389337618 |
| 267 | gene164 | 0.813445 | 9.019689 | 0.090185 | gene267 | 0.404435806 | gene410 | 0.83228174 | gene128 | 1.391419534 |
| 268 | gene244 | 0.827484 | 9.125461 | 0.090679 | gene305 | 0.404595244 | gene392 | 0.83368334 | gene123 | 1.391984756 |
| 269 | gene434 | 0.789635 | 8.667548 | 0.091102 | gene204 | 0.404635004 | gene150 | 0.83407914 | gene445 | 1.392590882 |
| 270 | gene350 | 0.814537 | 8.899919 | 0.091522 | gene180 | 0.40499321 | gene125 | 0.83477393 | gene410 | 1.395337714 |
| 271 | gene239 | 0.826809 | 8.968041 | 0.092195 | gene250 | 0.405297582 | gene106 | 0.83654079 | gene127 | 1.396249927 |
| 272 | gene306 | 0.790869 | 8.553637 | 0.09246 | gene127 | 0.4053364 | gene227 | 0.83850811 | gene412 | 1.397210121 |
| 273 | gene294 | 0.826798 | 8.936902 | 0.092515 | gene323 | 0.405621786 | gene211 | 0.83927968 | gene183 | 1.397519845 |
| 274 | gene427 | 0.822389 | 8.881709 | 0.092594 | gene295 | 0.405641312 | gene435 | 0.84059155 | gene227 | 1.398674662 |
| 275 | gene366 | 0.833754 | 9.000673 | 0.092632 | gene443 | 0.406670973 | gene103 | 0.84216591 | gene343 | 1.399166367 |
| 276 | gene152 | 0.827771 | 8.916683 | 0.092834 | gene473 | 0.406775557 | gene452 | 0.84436978 | gene106 | 1.401825769 |
| 277 | gene170 | 0.803491 | 8.652583 | 0.092861 | gene318 | 0.407187085 | gene320 | 0.8451109 | gene212 | 1.402701035 |
| 278 | gene477 | 0.823846 | 8.866619 | 0.092915 | gene419 | 0.407825952 | gene353 | 0.85000353 | gene255 | 1.402867549 |
| 279 | gene440 | 0.770032 | 8.273464 | 0.093072 | gene442 | 0.408170329 | gene200 | 0.85003869 | gene423 | 1.404907445 |
| 280 | gene132 | 0.845438 | 9.066568 | 0.093248 | gene200 | 0.410104267 | gene171 | 0.85398155 | gene149 | 1.405472578 |
| 281 | gene452 | 0.836379 | 8.969213 | 0.09325 | gene445 | 0.412066868 | gene185 | 0.85445939 | gene232 | 1.406125968 |
| 282 | gene336 | 0.818353 | 8.760153 | 0.093418 | gene139 | 0.413468232 | gene399 | 0.85492521 | gene165 | 1.406154355 |
| 283 | gene326 | 0.846419 | 9.055237 | 0.093473 | gene206 | 0.413865008 | gene120 | 0.85532635 | gene267 | 1.407392954 |
| 284 | gene107 | 0.805465 | 8.604301 | 0.093612 | gene493 | 0.415157533 | gene176 | 0.85545871 | gene120 | 1.407759232 |
| 285 | gene250 | 0.860637 | 9.167989 | 0.093874 | gene428 | 0.415223414 | gene447 | 0.85593013 | gene352 | 1.409215187 |
| 286 | gene328 | 0.867913 | 9.233842 | 0.093993 | gene110 | 0.416970875 | gene127 | 0.85633704 | gene289 | 1.409444008 |
| 287 | gene201 | 0.867522 | 9.216556 | 0.094126 | gene103 | 0.417866625 | gene246 | 0.85648782 | gene387 | 1.410357757 |
| 288 | gene271 | 0.82349 | 8.741849 | 0.094201 | gene171 | 0.417998582 | gene323 | 0.85651873 | gene430 | 1.411166092 |
| 289 | gene468 | 0.845075 | 8.964495 | 0.094269 | gene328 | 0.418435028 | gene218 | 0.85684039 | gene338 | 1.411714371 |
| 290 | gene361 | 0.865708 | 9.183063 | 0.094272 | gene136 | 0.418457836 | gene303 | 0.85800884 | gene185 | 1.414960702 |
| 291 | gene356 | 0.83044 | 8.783997 | 0.09454 | gene488 | 0.419618769 | gene434 | 0.85801143 | gene432 | 1.415643847 |
| 292 | gene202 | 0.735624 | 7.777121 | 0.094588 | gene165 | 0.419800928 | gene487 | 0.85876788 | gene157 | 1.415984394 |
| 293 | gene292 | 0.752413 | 7.949089 | 0.094654 | gene335 | 0.419935658 | gene277 | 0.8595279 | gene266 | 1.416101609 |
| 294 | gene310 | 0.875348 | 9.244833 | 0.094685 | gene343 | 0.422198511 | gene149 | 0.86140227 | gene422 | 1.41681048 |
| 295 | gene177 | 0.853001 | 8.996091 | 0.094819 | gene313 | 0.422489814 | gene253 | 0.86294527 | gene229 | 1.4184979 |
| 296 | gene351 | 0.875613 | 9.233554 | 0.094829 | gene246 | 0.422729324 | gene425 | 0.86388485 | gene251 | 1.418914908 |
| 297 | gene372 | 0.828042 | 8.718618 | 0.094974 | gene280 | 0.422917915 | gene147 | 0.86501182 | gene470 | 1.420013865 |
| 298 | gene272 | 0.81639 | 8.588722 | 0.095054 | gene468 | 0.424141973 | gene393 | 0.86501864 | gene206 | 1.422222855 |
| 299 | gene145 | 0.84742 | 8.860343 | 0.095642 | gene232 | 0.424885961 | gene143 | 0.86539012 | gene305 | 1.422489676 |
| 300 | gene459 | 0.803817 | 8.379312 | 0.095929 | gene455 | 0.425687293 | gene178 | 0.86583945 | gene353 | 1.423896357 |
| 301 | gene414 | 0.8599 | 8.931652 | 0.096276 | gene416 | 0.425894514 | gene343 | 0.86585847 | gene374 | 1.42393896 |
| 302 | gene161 | 0.901267 | 9.351437 | 0.096377 | gene218 | 0.426150534 | gene267 | 0.86646103 | gene246 | 1.423998533 |
| 303 | gene304 | 0.864811 | 8.964304 | 0.096473 | gene157 | 0.427965228 | gene153 | 0.86655333 | gene434 | 1.424358665 |
| 304 | gene308 | 0.81586 | 8.449066 | 0.096562 | gene176 | 0.428004818 | gene183 | 0.86706897 | gene125 | 1.42506908 |
| 305 | gene321 | 0.854524 | 8.831586 | 0.096758 | gene452 | 0.428115291 | gene157 | 0.86717202 | gene138 | 1.42621387 |
| 306 | gene497 | 0.873733 | 9.025219 | 0.09681 | gene118 | 0.428174936 | gene432 | 0.86767806 | gene122 | 1.427608112 |
| 307 | gene291 | 0.874661 | 8.959793 | 0.097621 | gene447 | 0.428207307 | gene165 | 0.8687243 | gene436 | 1.432078922 |
| 308 | gene228 | 0.844266 | 8.638742 | 0.09773 | gene255 | 0.430619384 | gene369 | 0.86915284 | gene399 | 1.432377261 |
| 309 | gene401 | 0.85867 | 8.737563 | 0.098273 | gene185 | 0.430716 | gene488 | 0.8692004 | gene315 | 1.432671277 |
| 310 | gene421 | 0.879326 | 8.885145 | 0.098966 | gene212 | 0.430957105 | gene231 | 0.87050506 | gene171 | 1.433875336 |
| 311 | gene203 | 0.836858 | 8.402181 | 0.0996 | gene303 | 0.431127043 | gene406 | 0.87097176 | gene184 | 1.436504489 |
| 312 | gene419 | 0.905193 | 9.082334 | 0.099665 | gene439 | 0.432432902 | gene311 | 0.87237574 | gene334 | 1.44231052 |
| 313 | gene496 | 0.903923 | 9.066618 | 0.099698 | gene425 | 0.432664907 | gene204 | 0.87246387 | gene307 | 1.442416582 |
| 314 | gene278 | 0.869053 | 8.706734 | 0.099814 | gene253 | 0.434906726 | gene387 | 0.87367382 | gene337 | 1.444662069 |
| 315 | gene136 | 0.907395 | 9.068083 | 0.100065 | gene197 | 0.435331112 | gene175 | 0.87651767 | gene143 | 1.446219936 |
| 316 | gene388 | 0.811993 | 8.098962 | 0.100259 | gene189 | 0.435962679 | gene470 | 0.8784062 | gene214 | 1.45374245 |
| 317 | gene373 | 0.865372 | 8.619766 | 0.100394 | gene122 | 0.436681756 | gene428 | 0.8785196 | gene420 | 1.454182696 |
| 318 | gene235 | 0.870955 | 8.667581 | 0.100484 | gene412 | 0.436876983 | gene365 | 0.87922669 | gene472 | 1.454562757 |
| 319 | gene268 | 0.863485 | 8.592693 | 0.100491 | gene266 | 0.437021019 | gene439 | 0.87965724 | gene328 | 1.454609219 |
| 320 | gene408 | 0.879226 | 8.746405 | 0.100524 | gene143 | 0.439693514 | gene132 | 0.87992553 | gene311 | 1.454897633 |
| 321 | gene192 | 0.923736 | 9.15504 | 0.100899 | gene279 | 0.439811162 | gene473 | 0.88132802 | gene274 | 1.45549516 |
| 322 | gene181 | 0.881375 | 8.714037 | 0.101144 | gene320 | 0.439915791 | gene351 | 0.88205418 | gene406 | 1.455602082 |
| 323 | gene413 | 0.869309 | 8.568335 | 0.101456 | gene376 | 0.44009055 | gene335 | 0.88265973 | gene447 | 1.456717023 |
| 324 | gene301 | 0.885861 | 8.689777 | 0.101943 | gene406 | 0.440412354 | gene230 | 0.88275336 | gene243 | 1.457776844 |
| 325 | gene407 | 0.85394 | 8.37487 | 0.101965 | gene471 | 0.440843916 | gene417 | 0.88373342 | gene114 | 1.457829999 |
| 326 | gene450 | 0.934363 | 9.161571 | 0.101987 | gene414 | 0.441575051 | gene397 | 0.8857311 | gene250 | 1.45844442 |
| 327 | gene257 | 0.901797 | 8.834667 | 0.102075 | gene208 | 0.442129525 | gene416 | 0.88676926 | gene301 | 1.459582261 |
| 328 | gene362 | 0.956066 | 9.353889 | 0.102211 | gene434 | 0.443884122 | gene114 | 0.88720054 | gene488 | 1.461896304 |
| 329 | gene248 | 0.856581 | 8.374891 | 0.10228 | gene245 | 0.444646684 | gene498 | 0.88747894 | gene178 | 1.462607397 |
| 330 | gene309 | 0.907682 | 8.850134 | 0.102561 | gene106 | 0.444930781 | gene245 | 0.88826111 | gene416 | 1.462770119 |
| 331 | gene296 | 0.88306 | 8.607028 | 0.102598 | gene114 | 0.448201005 | gene184 | 0.88906456 | gene259 | 1.463817469 |
| 332 | gene465 | 0.826679 | 8.045631 | 0.102749 | gene150 | 0.448376571 | gene249 | 0.88913385 | gene361 | 1.466319177 |
| 333 | gene382 | 0.904244 | 8.786928 | 0.102908 | gene195 | 0.448409333 | gene436 | 0.88922849 | gene216 | 1.467705152 |
| 334 | gene151 | 0.897991 | 8.718676 | 0.102996 | gene446 | 0.451051482 | gene420 | 0.88942006 | gene335 | 1.46866984 |
| 335 | gene344 | 0.90856 | 8.818202 | 0.103032 | gene361 | 0.451332767 | gene446 | 0.88943361 | gene104 | 1.471321687 |
| 336 | gene175 | 0.844767 | 8.184789 | 0.103212 | gene175 | 0.451972239 | gene212 | 0.8894737 | gene153 | 1.471485376 |
| 337 | gene116 | 0.911617 | 8.820612 | 0.103351 | gene132 | 0.452137708 | gene271 | 0.89056628 | gene170 | 1.472214661 |
| 338 | gene457 | 0.907726 | 8.782945 | 0.103351 | gene436 | 0.45240231 | gene202 | 0.89197429 | gene163 | 1.473209059 |
| 339 | gene238 | 0.915621 | 8.849587 | 0.103465 | gene393 | 0.453804737 | gene471 | 0.89316803 | gene427 | 1.475907574 |
| 340 | gene141 | 0.923878 | 8.927304 | 0.103489 | gene254 | 0.45436426 | gene251 | 0.8938861 | gene397 | 1.476857486 |
| 341 | gene241 | 0.907674 | 8.768944 | 0.10351 | gene334 | 0.456373231 | gene100 | 0.894194 | gene394 | 1.477856343 |
| 342 | gene182 | 0.924668 | 8.898705 | 0.10391 | gene231 | 0.460972028 | gene122 | 0.89475645 | gene209 | 1.478281474 |
| 343 | gene189 | 0.96106 | 9.244609 | 0.103959 | gene241 | 0.461038018 | gene241 | 0.89613893 | gene417 | 1.47835299 |
| 344 | gene195 | 0.951948 | 9.154868 | 0.103983 | gene301 | 0.461747408 | gene352 | 0.89622572 | gene276 | 1.478595506 |
| 345 | gene156 | 0.94545 | 9.089741 | 0.104013 | gene235 | 0.462926636 | gene299 | 0.89668648 | gene495 | 1.47906363 |
| 346 | gene168 | 0.917654 | 8.813042 | 0.104125 | gene487 | 0.463286214 | gene468 | 0.89735323 | gene322 | 1.479562255 |
| 347 | gene118 | 0.954421 | 9.159942 | 0.104195 | gene417 | 0.464056544 | gene361 | 0.89759785 | gene471 | 1.48129282 |
| 348 | gene293 | 0.934976 | 8.972273 | 0.104207 | gene369 | 0.465028664 | gene274 | 0.89857541 | gene245 | 1.481584065 |
| 349 | gene313 | 0.967667 | 9.279843 | 0.104276 | gene497 | 0.465249347 | gene310 | 0.89965306 | gene313 | 1.484124579 |
| 350 | gene467 | 0.919752 | 8.817026 | 0.104315 | gene259 | 0.466754826 | gene104 | 0.9001029 | gene193 | 1.485222828 |
| 351 | gene213 | 0.89893 | 8.613682 | 0.104361 | gene23894 | 0.46708195 | gene472 | 0.90145627 | gene376 | 1.48567211 |
| 352 | gene197 | 0.963253 | 9.224404 | 0.104424 | gene353 | 0.467515197 | gene276 | 0.90194188 | gene103 | 1.489753521 |
| 353 | gene346 | 0.901895 | 8.627369 | 0.104539 | gene6845 | 0.467612298 | gene328 | 0.90226944 | gene459 | 1.491194498 |
| 354 | gene473 | 0.926259 | 8.84867 | 0.104678 | gene170 | 0.468499418 | gene209 | 0.90499237 | gene393 | 1.491460838 |
| 355 | gene333 | 0.93528 | 8.934707 | 0.104679 | gene420 | 0.468621035 | gene163 | 0.90571778 | gene249 | 1.49192217 |
| 356 | gene359 | 0.914436 | 8.728996 | 0.104758 | gene14522 | 0.468639423 | gene279 | 0.90626312 | gene398 | 1.492698508 |
| 357 | gene256 | 0.883104 | 8.42395 | 0.104833 | gene1878 | 0.468816356 | gene307 | 0.90651483 | gene493 | 1.493713444 |
| 358 | gene479 | 0.903069 | 8.609626 | 0.104891 | gene43244 | 0.468911685 | gene291 | 0.9065319 | gene241 | 1.493979354 |
| 359 | gene240 | 0.933423 | 8.895262 | 0.104935 | gene120 | 0.469114658 | gene315 | 0.90665414 | gene151 | 1.494668399 |
| 360 | gene446 | 0.951557 | 9.039031 | 0.105272 | gene47545 | 0.469547146 | gene266 | 0.90682263 | gene292 | 1.495299776 |
| 361 | gene263 | 0.93736 | 8.892515 | 0.10541 | gene3725 | 0.469602315 | gene334 | 0.90969578 | gene202 | 1.496401528 |
| 362 | gene332 | 0.930104 | 8.81842 | 0.105473 | gene42896 | 0.469785782 | gene414 | 0.90975602 | gene239 | 1.49729993 |
| 363 | gene324 | 0.941639 | 8.920593 | 0.105558 | gene14751 | 0.46982705 | gene484 | 0.90982275 | gene351 | 1.497973847 |
| 364 | gene215 | 0.816851 | 7.692061 | 0.106194 | gene251 | 0.470217263 | gene214 | 0.91096198 | gene365 | 1.498369976 |
| 365 | gene466 | 0.954824 | 8.972622 | 0.106415 | gene24669 | 0.470285466 | gene333 | 0.91125142 | gene164 | 1.499239098 |
| 366 | gene396 | 0.942511 | 8.828524 | 0.106757 | gene33517 | 0.47093646 | gene250 | 0.91133556 | gene294 | 1.500213631 |
| 367 | gene390 | 0.953764 | 8.931212 | 0.10679 | gene19457 | 0.471207847 | gene322 | 0.91158344 | gene299 | 1.501886705 |
| 368 | gene330 | 0.859619 | 8.019636 | 0.107189 | gene23684 | 0.47131377 | gene173 | 0.91222769 | gene145 | 1.505401105 |
| 369 | gene297 | 0.948151 | 8.839126 | 0.107267 | gene277 | 0.471554533 | gene195 | 0.91276144 | gene333 | 1.506172611 |
| 370 | gene349 | 0.957958 | 8.905138 | 0.107574 | gene8230 | 0.471601626 | gene177 | 0.91342445 | gene235 | 1.507500793 |
| 371 | gene254 | 0.976187 | 9.074092 | 0.10758 | gene12162 | 0.471678587 | gene456 | 0.91375792 | gene215 | 1.508016049 |
| 372 | gene494 | 0.973631 | 8.966339 | 0.108587 | gene43097 | 0.47195252 | gene192 | 0.91418167 | gene321 | 1.508876541 |
| 373 | gene158 | 0.94902 | 8.726153 | 0.108756 | gene244 | 0.471975763 | gene337 | 0.91479323 | gene230 | 1.510373084 |
| 374 | gene375 | 0.905719 | 8.324524 | 0.108801 | gene230 | 0.472531283 | gene158 | 0.91519069 | gene161 | 1.512451199 |
| 375 | gene134 | 0.931939 | 8.55648 | 0.108916 | gene46459 | 0.472588295 | gene254 | 0.91548582 | gene452 | 1.51318955 |
| 376 | gene242 | 0.919172 | 8.43408 | 0.108983 | gene49425 | 0.472681759 | gene201 | 0.91603373 | gene401 | 1.516435683 |
| 377 | gene260 | 0.926152 | 8.492012 | 0.109062 | gene40030 | 0.472917171 | gene215 | 0.91642165 | gene173 | 1.516720645 |
| 378 | gene498 | 0.927123 | 8.44667 | 0.109762 | gene151 | 0.472975968 | gene275 | 0.91732317 | gene341 | 1.516994919 |
| 379 | gene109 | 0.911284 | 8.297242 | 0.10983 | gene432 | 0.473121433 | gene189 | 0.91821439 | gene100 | 1.517316276 |
| 380 | gene262 | 0.962461 | 8.757529 | 0.109901 | gene18467 | 0.473293943 | gene228 | 0.91993408 | gene350 | 1.517321811 |
| 381 | gene279 | 0.926015 | 8.42083 | 0.109967 | gene311 | 0.473325968 | gene161 | 0.92296652 | gene421 | 1.518830695 |
| 382 | gene429 | 0.953969 | 8.592472 | 0.111024 | gene149 | 0.473574885 | gene193 | 0.9231643 | gene418 | 1.520742968 |
| 383 | gene144 | 0.945887 | 8.51121 | 0.111134 | gene14156 | 0.473661153 | gene136 | 0.92406379 | gene271 | 1.522291043 |
| 384 | gene444 | 0.955766 | 8.583329 | 0.111351 | gene33834 | 0.473708988 | gene313 | 0.92408593 | gene310 | 1.522700086 |
| 385 | gene426 | 0.979647 | 8.769946 | 0.111705 | gene27293 | 0.473851202 | gene243 | 0.92424839 | gene189 | 1.522944783 |
| 386 | gene492 | 0.949122 | 8.474543 | 0.111997 | gene33225 | 0.473926225 | gene301 | 0.92519885 | gene336 | 1.52486725 |
| 387 | gene258 | 0.985582 | 8.795603 | 0.112054 | gene6887 | 0.47392681 | gene259 | 0.92617456 | gene448 | 1.525132758 |
| 388 | gene339 | 0.978358 | 8.720863 | 0.112186 | gene11216 | 0.474023108 | gene457 | 0.92659345 | gene450 | 1.525390679 |
| 389 | gene131 | 0.929009 | 8.27317 | 0.112292 | gene12497 | 0.474093536 | gene376 | 0.92710591 | gene456 | 1.525397232 |
| 390 | gene111 | 0.914689 | 8.136084 | 0.112424 | gene153 | 0.474100749 | gene419 | 0.9273841 | gene136 | 1.526486656 |
| 391 | gene384 | 0.957335 | 8.473399 | 0.112981 | gene394 | 0.474284211 | gene495 | 0.92813355 | gene497 | 1.52817296 |
| 392 | gene357 | 0.975234 | 8.620532 | 0.113129 | gene9850 | 0.474408222 | gene362 | 0.92889592 | gene475 | 1.52979717 |
| 393 | gene119 | 0.974437 | 8.560261 | 0.113833 | gene22344 | 0.474707408 | gene247 | 0.92906899 | gene476 | 1.532066145 |
| 394 | gene252 | 0.943476 | 8.146548 | 0.115813 | gene38265 | 0.474775557 | gene170 | 0.9316241 | gene379 | 1.532133666 |
| 395 | gene166 | 0.938349 | 8.084295 | 0.116071 | gene201 | 0.475165463 | gene394 | 0.93216311 | gene297 | 1.534217209 |
| 396 | gene284 | 0.959711 | 8.050217 | 0.119216 | gene31481 | 0.475400624 | gene152 | 0.93286487 | gene175 | 1.534358385 |
| 397 | gene283 | 0.975767 | 8.018395 | 0.121691 | gene141 | 0.475514405 | gene427 | 0.93299681 | gene396 | 1.534560973 |
| 398 | gene449 | 0.985181 | 8.032723 | 0.122646 | gene11796 | 0.475593937 | gene341 | 0.93336209 | gene309 | 1.537007974 |
| 399 | gene179 | 0.959865 | 7.807317 | 0.122944 | gene17470 | 0.475661987 | gene459 | 0.93437999 | gene419 | 1.537205494 |
| 400 | gene210 | 0.934811 | 7.197833 | 0.129874 | gene16957 | 0.476010724 | gene413 | 0.93535551 | gene304 | 1.53763436 |

## Table S3: Reference candidate genes full list for the PRJNA659517 transcriptome. The rank order (GSV ID) of the VectorBase genes (ID) was based on the coefficient of variation (CV). TPM avrg: TPM average.

| **GSV ID** | **ID** | **SD** | **TPM avrg** | **CV** |
| --- | --- | --- | --- | --- |
| 1 | AAEL007824-RA | 0.2582342022 | 11.03092373 | 0.02341002518 |
| 2 | AAEL006564-RB | 0.1532686361 | 5.575119456 | 0.02749154298 |
| 3 | AAEL004378-RA | 0.2025719112 | 7.10817673 | 0.02849843482 |
| 4 | AAEL020737-RA | 0.3369837509 | 11.73293729 | 0.0287211755 |
| 5 | AAEL007135-RA | 0.2411160904 | 8.224337392 | 0.02931738801 |
| 6 | AAEL012279-RA | 0.1855996359 | 6.326160427 | 0.02933843333 |
| 7 | AAEL017494-RA | 0.3672400944 | 12.01070262 | 0.03057607085 |
| 8 | AAEL024536-RA | 0.3241791183 | 10.25607642 | 0.03160849288 |
| 9 | AAEL006860-RA | 0.3542306044 | 10.90460494 | 0.0324844968 |
| 10 | AAEL005817-RA | 0.3387702918 | 10.41217696 | 0.0325359714 |
| 11 | AAEL022104-RA | 0.3253474773 | 9.638242606 | 0.03375589209 |
| 12 | AAEL001479-RA | 0.2244602147 | 6.595434177 | 0.03403266694 |
| 13 | AAEL009496-RA | 0.3313948901 | 9.724427949 | 0.03407859998 |
| 14 | AAEL007771-RB | 0.3727860617 | 10.91444078 | 0.03415530574 |
| 15 | AAEL003582-RA | 0.3432268995 | 9.741662375 | 0.03523288801 |
| 16 | AAEL000795-RA | 0.2912216968 | 8.256192652 | 0.03527312274 |
| 17 | AAEL020290-RA | 0.3589094489 | 10.15341314 | 0.03534865015 |
| 18 | AAEL007385-RF | 0.3200223253 | 8.973370447 | 0.03566355888 |
| 19 | AAEL024434-RA | 0.3848163346 | 10.77115819 | 0.03572655119 |
| 20 | AAEL009151-RA | 0.3593585461 | 10.04839533 | 0.03576277946 |
| 21 | AAEL008188-RA | 0.3645786587 | 10.13483333 | 0.0359728322 |
| 22 | AAEL011471-RF | 0.3911661412 | 10.78175324 | 0.0362803834 |
| 23 | AAEL023769-RA | 0.3511619658 | 9.620664719 | 0.03650080073 |
| 24 | AAEL017081-RA | 0.275876728 | 7.508182122 | 0.03674347845 |
| 25 | AAEL004325-RA | 0.3731994877 | 10.11786762 | 0.0368851918 |
| 26 | AAEL002047-RA | 0.3770296866 | 10.18453032 | 0.03701984035 |
| 27 | AAEL011992-RC | 0.3304218985 | 8.905286234 | 0.03710401775 |
| 28 | AAEL013536-RB | 0.3959799736 | 10.64068228 | 0.03721377662 |
| 29 | AAEL002639-RA | 0.3766301806 | 10.1045798 | 0.0372732155 |
| 30 | AAEL010843-RA | 0.2537495716 | 6.798351717 | 0.03732516088 |
| 31 | AAEL007715-RC | 0.3832885685 | 10.25544205 | 0.03737416357 |
| 32 | AAEL004175-RA | 0.3911226167 | 10.45591771 | 0.03740681858 |
| 33 | AAEL017468-RA | 0.4841400786 | 12.86865718 | 0.03762164705 |
| 34 | AAEL022286-RA | 0.3994937682 | 10.58947692 | 0.03772554313 |
| 35 | AAEL013272-RA | 0.3760987104 | 9.956788445 | 0.03777309446 |
| 36 | AAEL010095-RE | 0.2100526792 | 5.529914563 | 0.03798479648 |
| 37 | AAEL028141-RA | 0.2232668083 | 5.877008874 | 0.03798987088 |
| 38 | AAEL012686-RA | 0.4142965365 | 10.89634264 | 0.03802161423 |
| 39 | AAEL010318-RA | 0.306388725 | 8.048198011 | 0.03806923296 |
| 40 | AAEL000032-RE | 0.3655421248 | 9.560752638 | 0.03823361388 |
| 41 | AAEL013625-RA | 0.3913498051 | 10.20927052 | 0.03833278826 |
| 42 | AAEL019937-RA | 0.2982964466 | 7.774557229 | 0.03836828745 |
| 43 | AAEL018662-RA | 0.5010605832 | 13.03740651 | 0.03843253509 |
| 44 | AAEL010977-RA | 0.3221157322 | 8.352365198 | 0.03856581035 |
| 45 | AAEL009351-RA | 0.286619778 | 7.39592732 | 0.03875373102 |
| 46 | AAEL007699-RG | 0.3916474479 | 9.9681506 | 0.0392898807 |
| 47 | AAEL012317-RA | 0.3004682297 | 7.642749693 | 0.03931415287 |
| 48 | AAEL007383-RG | 0.3347145562 | 8.497749834 | 0.03938861025 |
| 49 | AAEL011587-RC | 0.3983629927 | 10.03023806 | 0.03971620515 |
| 50 | AAEL019568-RD | 0.3104351782 | 7.689068165 | 0.04037357604 |
| 51 | AAEL019998-RA | 0.2243294833 | 5.503805021 | 0.04075898082 |
| 52 | AAEL021083-RA | 0.4249683531 | 10.41020227 | 0.04082229548 |
| 53 | AAEL017198-RA | 0.4359701924 | 10.66713005 | 0.04087043003 |
| 54 | AAEL014932-RC | 0.3533263166 | 8.610659239 | 0.04103359648 |
| 55 | AAEL009747-RA | 0.4059881592 | 9.875497271 | 0.04111065479 |
| 56 | AAEL018669-RA | 0.5331667401 | 12.96560203 | 0.0411216339 |
| 57 | AAEL010299-RC | 0.4336701771 | 10.49763733 | 0.04131121734 |
| 58 | AAEL023209-RA | 0.2549712593 | 6.171388309 | 0.04131505693 |
| 59 | AAEL005170-RB | 0.4114219688 | 9.941969419 | 0.04138234101 |
| 60 | AAEL011447-RB | 0.43058186 | 10.34896887 | 0.04160625715 |
| 61 | AAEL009994-RA | 0.3979884426 | 9.556503737 | 0.04164582085 |
| 62 | AAEL005097-RB | 0.3927989352 | 9.430218545 | 0.04165321655 |
| 63 | AAEL005451-RA | 0.4471762313 | 10.73324202 | 0.04166273624 |
| 64 | AAEL009653-RA | 0.4080129272 | 9.724847687 | 0.04195571389 |
| 65 | AAEL002372-RB | 0.4470520663 | 10.61874845 | 0.04210025961 |
| 66 | AAEL012939-RA | 0.3073663412 | 7.297692453 | 0.04211829193 |
| 67 | AAEL001763-RA | 0.3963507976 | 9.358393839 | 0.04235243829 |
| 68 | AAEL008481-RA | 0.4486548046 | 10.56250962 | 0.04247615582 |
| 69 | AAEL023983-RA | 0.4306506114 | 10.11158331 | 0.04258982972 |
| 70 | AAEL004783-RA | 0.3407226773 | 7.91022274 | 0.04307371467 |
| 71 | AAEL008103-RB | 0.4516649421 | 10.47741117 | 0.0431084487 |
| 72 | AAEL004151-RF | 0.4525493757 | 10.47097186 | 0.04321942429 |
| 73 | AAEL010756-RD | 0.4589466607 | 10.61450869 | 0.04323767346 |
| 74 | AAEL006698-RA | 0.4335457351 | 10.02512877 | 0.04324590187 |
| 75 | AAEL015575-RC | 0.2927396987 | 6.746142926 | 0.04339364018 |
| 76 | AAEL017413-RA | 0.7428376553 | 17.07732963 | 0.04349846677 |
| 77 | AAEL006520-RB | 0.2448924307 | 5.628618564 | 0.04350844313 |
| 78 | AAEL014562-RA | 0.4531487028 | 10.37601733 | 0.04367270103 |
| 79 | AAEL005901-RC | 0.4515730833 | 10.31618167 | 0.04377327754 |
| 80 | AAEL017516-RB | 0.4185757678 | 9.529196947 | 0.04392560781 |
| 81 | AAEL013139-RG | 0.273372209 | 6.179013594 | 0.04424204686 |
| 82 | AAEL002832-RA | 0.4706564471 | 10.57798955 | 0.04449394139 |
| 83 | AAEL009979-RA | 0.3614240964 | 8.11692372 | 0.04452722594 |
| 84 | AAEL001852-RB | 0.2812956572 | 6.281553991 | 0.04478122095 |
| 85 | AAEL005266-RC | 0.4573585588 | 10.14808615 | 0.04506845448 |
| 86 | AAEL003427-RA | 0.4705401309 | 10.39045213 | 0.04528581864 |
| 87 | AAEL010777-RA | 0.3735418406 | 8.231334015 | 0.04538047416 |
| 88 | AAEL022415-RA | 0.4194612851 | 9.220659468 | 0.04549146257 |
| 89 | AAEL022140-RA | 0.5415427901 | 11.90135677 | 0.04550260955 |
| 90 | AAEL004069-RA | 0.2840011765 | 6.223000383 | 0.04563733874 |
| 91 | AAEL007821-RA | 0.2578428035 | 5.584760615 | 0.04616899833 |
| 92 | AAEL014944-RA | 0.4879527306 | 10.52858276 | 0.04634552835 |
| 93 | AAEL004500-RD | 0.4530718344 | 9.733255683 | 0.04654884749 |
| 94 | AAEL020749-RA | 0.4833649777 | 10.34773907 | 0.04671213437 |
| 95 | AAEL008329-RA | 0.4603660165 | 9.849194295 | 0.04674148999 |
| 96 | AAEL004985-RB | 0.3020339111 | 6.460183826 | 0.04675314499 |
| 97 | AAEL011656-RA | 0.4892427353 | 10.41418207 | 0.04697850796 |
| 98 | AAEL028657-RA | 0.6121437757 | 12.98495099 | 0.04714255573 |
| 99 | AAEL014064-RB | 0.2791685254 | 5.907641864 | 0.04725549242 |
| 100 | AAEL013359-RD | 0.3921793423 | 8.295543755 | 0.04727590545 |
| 101 | AAEL001109-RA | 0.2626200002 | 5.553280403 | 0.04729096699 |
| 102 | AAEL000823-RA | 0.4808442886 | 10.04008577 | 0.04789244831 |
| 103 | AAEL005722-RB | 0.4953969668 | 10.29377055 | 0.04812589948 |
| 104 | AAEL017231-RA | 0.6612984553 | 13.73402433 | 0.04815037743 |
| 105 | AAEL022341-RA | 0.4673638323 | 9.703732006 | 0.04816330789 |
| 106 | AAEL013739-RA | 0.3282960808 | 6.798001877 | 0.04829302591 |
| 107 | AAEL010051-RA | 0.2618801309 | 5.416268337 | 0.04835065668 |
| 108 | AAEL010673-RA | 0.4894649497 | 10.10174525 | 0.04845350359 |
| 109 | AAEL014292-RB | 0.4750923883 | 9.794669293 | 0.04850519952 |
| 110 | AAEL025553-RA | 0.4663817449 | 9.596429692 | 0.04859950626 |
| 111 | AAEL005509-RA | 0.2558588482 | 5.25483613 | 0.0486901669 |
| 112 | AAEL014379-RB | 0.2781363892 | 5.706981857 | 0.04873616144 |
| 113 | AAEL007383-RE | 0.3423267689 | 6.990995477 | 0.04896681309 |
| 114 | AAEL005629-RA | 0.479863117 | 9.75103439 | 0.04921150903 |
| 115 | AAEL008192-RA | 0.4818522913 | 9.779059953 | 0.04927388661 |
| 116 | AAEL002534-RA | 0.4890781828 | 9.922211151 | 0.04929124924 |
| 117 | AAEL003942-RA | 0.5257381376 | 10.6282677 | 0.04946602331 |
| 118 | AAEL003160-RA | 0.4413029234 | 8.906825013 | 0.04954660306 |
| 119 | AAEL028043-RB | 0.5062351569 | 10.21305553 | 0.04956745368 |
| 120 | AAEL025321-RA | 0.2883941701 | 5.817930932 | 0.04956988549 |
| 121 | AAEL007681-RA | 0.3930607926 | 7.92821674 | 0.04957745297 |
| 122 | AAEL009341-RA | 0.4663405454 | 9.373056182 | 0.04975330739 |
| 123 | AAEL006511-RA | 0.4842514752 | 9.720424656 | 0.04981793412 |
| 124 | AAEL014630-RA | 0.3389130066 | 6.802778355 | 0.04981979258 |
| 125 | AAEL002833-RA | 0.406564414 | 8.151789951 | 0.04987425049 |
| 126 | AAEL011327-RA | 0.382147116 | 7.65533142 | 0.04991908189 |
| 127 | AAEL009257-RA | 0.537413745 | 10.75539276 | 0.04996691027 |
| 128 | AAEL022384-RA | 0.3019505342 | 6.0397654 | 0.04999375212 |
| 129 | AAEL000182-RA | 0.5884162347 | 11.69358553 | 0.05031957335 |
| 130 | AAEL019789-RA | 0.4152577348 | 8.245701832 | 0.05036050821 |
| 131 | AAEL009201-RA | 0.5688972715 | 11.28988648 | 0.0503899904 |
| 132 | AAEL025362-RA | 0.4493809577 | 8.894488625 | 0.05052352942 |
| 133 | AAEL015006-RB | 0.516320136 | 10.14783017 | 0.05087985583 |
| 134 | AAEL007526-RD | 0.2740502423 | 5.372713125 | 0.05100779362 |
| 135 | AAEL009570-RA | 0.4343633446 | 8.493733575 | 0.05113927118 |
| 136 | AAEL012026-RE | 0.3815255117 | 7.455371863 | 0.05117457837 |
| 137 | AAEL002526-RA | 0.4134215284 | 8.077426517 | 0.05118233233 |
| 138 | AAEL008249-RE | 0.2775761615 | 5.38399313 | 0.05155581644 |
| 139 | AAEL012010-RA | 0.3189959962 | 6.182294115 | 0.05159832098 |
| 140 | AAEL005897-RC | 0.3493487818 | 6.754862881 | 0.05171811597 |
| 141 | AAEL002737-RC | 0.5034086846 | 9.726399443 | 0.05175694126 |
| 142 | AAEL026109-RD | 0.2818684959 | 5.43212939 | 0.05188913511 |
| 143 | AAEL003055-RA | 0.2742807747 | 5.276169104 | 0.05198483394 |
| 144 | AAEL006785-RB | 0.4409569894 | 8.457863482 | 0.05213574212 |
| 145 | AAEL021422-RA | 0.4409592365 | 8.457864663 | 0.05213600053 |
| 146 | AAEL027408-RC | 0.3980964723 | 7.624503033 | 0.05221277643 |
| 147 | AAEL003417-RA | 0.36370119 | 6.954625543 | 0.0522963009 |
| 148 | AAEL000987-RA | 0.5280721499 | 10.08780038 | 0.05234760107 |
| 149 | AAEL018667-RA | 0.5546316705 | 10.57029235 | 0.05247079759 |
| 150 | AAEL018664-RA | 0.5931203474 | 11.28066013 | 0.05257851409 |
| 151 | AAEL006065-RA | 0.3086153602 | 5.85296846 | 0.0527280067 |
| 152 | AAEL011157-RA | 0.4561339359 | 8.62249148 | 0.05290047974 |
| 153 | AAEL003396-RA | 0.4985247631 | 9.420966202 | 0.05291652177 |
| 154 | AAEL010012-RA | 0.3522335242 | 6.646602013 | 0.05299452615 |
| 155 | AAEL006304-RA | 0.407132504 | 7.678916094 | 0.05301952762 |
| 156 | AAEL018689-RA | 0.8036960675 | 15.14319755 | 0.05307307555 |
| 157 | AAEL005220-RD | 0.5267237009 | 9.900395783 | 0.05320228731 |
| 158 | AAEL018671-RA | 0.4890053226 | 9.175776269 | 0.05329307387 |
| 159 | AAEL014039-RD | 0.5748547671 | 10.77542212 | 0.05334870049 |
| 160 | AAEL000010-RA | 0.4695668883 | 8.79848466 | 0.05336906371 |
| 161 | AAEL009160-RC | 0.4140607146 | 7.749386447 | 0.05343141904 |
| 162 | AAEL029013-RA | 0.4576869439 | 8.564881251 | 0.05343762867 |
| 163 | AAEL012585-RA | 0.5205033589 | 9.700227501 | 0.05365888159 |
| 164 | AAEL023816-RA | 0.3082087033 | 5.723888306 | 0.05384603732 |
| 165 | AAEL011742-RA | 0.3242138856 | 6.020703154 | 0.05384983735 |
| 166 | AAEL025388-RA | 0.4259519745 | 7.907096261 | 0.05386958252 |
| 167 | AAEL000458-RD | 0.4920796463 | 9.123065473 | 0.0539379716 |
| 168 | AAEL017146-RA | 0.6604781847 | 12.21711492 | 0.05406171497 |
| 169 | AAEL013069-RA | 0.5412710697 | 10.00062505 | 0.05412372394 |
| 170 | AAEL001210-RA | 0.451158622 | 8.278102609 | 0.05450024521 |
| 171 | AAEL005190-RA | 0.3225133688 | 5.888669793 | 0.05476845877 |
| 172 | AAEL012552-RC | 0.4026821413 | 7.345018203 | 0.05482384524 |
| 173 | AAEL012944-RB | 0.5546514353 | 10.10494636 | 0.05488910238 |
| 174 | AAEL013221-RC | 0.5413216114 | 9.861203818 | 0.05489406988 |
| 175 | AAEL011156-RA | 0.3523984721 | 6.359447369 | 0.05541337976 |
| 176 | AAEL001759-RA | 0.5382935692 | 9.64397653 | 0.05581655737 |
| 177 | AAEL004149-RA | 0.5612353692 | 10.04125017 | 0.05589297743 |
| 178 | AAEL000766-RA | 0.7906302481 | 14.13249409 | 0.05594414144 |
| 179 | AAEL002881-RA | 0.6316879996 | 11.28588566 | 0.05597150446 |
| 180 | AAEL004829-RA | 0.4958463164 | 8.824329144 | 0.05619082293 |
| 181 | AAEL028695-RA | 0.6029096101 | 10.66918809 | 0.05650941805 |
| 182 | AAEL028707-RA | 0.6029096101 | 10.66918809 | 0.05650941805 |
| 183 | AAEL028736-RA | 0.6029124222 | 10.66919437 | 0.05650964836 |
| 184 | AAEL028738-RA | 0.6029128311 | 10.66919553 | 0.05650968057 |
| 185 | AAEL028769-RA | 0.6029135571 | 10.66919602 | 0.056509746 |
| 186 | AAEL028742-RA | 0.6029135571 | 10.66919602 | 0.056509746 |
| 187 | AAEL028798-RA | 0.6030048933 | 10.66952781 | 0.05651654917 |
| 188 | AAEL028650-RA | 0.6030155737 | 10.669116 | 0.05651973171 |
| 189 | AAEL028644-RA | 0.6030155737 | 10.669116 | 0.05651973171 |
| 190 | AAEL028658-RA | 0.6030168504 | 10.66911944 | 0.05651983315 |
| 191 | AAEL028668-RA | 0.6030219185 | 10.66912479 | 0.0565202798 |
| 192 | AAEL028682-RA | 0.6030255064 | 10.66913432 | 0.05652056563 |
| 193 | AAEL028852-RA | 0.6033036576 | 10.67099609 | 0.05653677055 |
| 194 | AAEL028856-RA | 0.6033101736 | 10.67100054 | 0.05653735759 |
| 195 | AAEL028858-RA | 0.603593675 | 10.6712715 | 0.05656248886 |
| 196 | AAEL028862-RA | 0.6035951116 | 10.67127349 | 0.05656261291 |
| 197 | AAEL028884-RA | 0.6035992253 | 10.67127659 | 0.05656298194 |
| 198 | AAEL028912-RA | 0.6036013891 | 10.67128538 | 0.05656313816 |
| 199 | AAEL028985-RA | 0.6036021756 | 10.6712877 | 0.05656319957 |
| 200 | AAEL028849-RA | 0.603602476 | 10.67033242 | 0.05656829162 |
| 201 | AAEL028994-RA | 0.6036985248 | 10.67155928 | 0.05657078868 |
| 202 | AAEL007243-RA | 0.2930916989 | 5.168305813 | 0.05670943428 |
| 203 | AAEL024747-RA | 0.3105704597 | 5.464696654 | 0.05683214995 |
| 204 | AAEL000958-RB | 0.4039519093 | 7.106846967 | 0.05683982097 |
| 205 | AAEL002401-RA | 0.3379439614 | 5.943983873 | 0.05685479111 |
| 206 | AAEL018685-RA | 0.6306498912 | 11.07004438 | 0.05696904815 |
| 207 | AAEL019403-RA | 0.5753266066 | 10.09083553 | 0.05701476405 |
| 208 | AAEL007718-RA | 0.372054045 | 6.522040464 | 0.05704565114 |
| 209 | AAEL021600-RA | 0.3477609952 | 6.076940965 | 0.05722632442 |
| 210 | AAEL013043-RA | 0.6043653766 | 10.5550025 | 0.05725866733 |
| 211 | AAEL026716-RA | 0.3197923696 | 5.569499947 | 0.05741850662 |
| 212 | AAEL003530-RA | 0.5882246934 | 10.23835036 | 0.05745307329 |
| 213 | AAEL028782-RA | 0.5432847688 | 9.451605482 | 0.0574806862 |
| 214 | AAEL012671-RB | 0.4491300821 | 7.784507031 | 0.05769537882 |
| 215 | AAEL000963-RA | 0.409991784 | 7.105833536 | 0.05769791566 |
| 216 | AAEL007886-RA | 0.489062375 | 8.452013633 | 0.05786341531 |
| 217 | AAEL008671-RA | 0.3046527133 | 5.25987504 | 0.05792014277 |
| 218 | AAEL012175-RA | 0.621059596 | 10.71649386 | 0.05795361841 |
| 219 | AAEL005143-RD | 0.6021145762 | 10.34497989 | 0.05820355212 |
| 220 | AAEL005508-RA | 0.5119340791 | 8.768045561 | 0.05838633883 |
| 221 | AAEL026806-RA | 0.5397782695 | 9.214351808 | 0.0585801672 |
| 222 | AAEL017826-RA | 0.4819697588 | 8.212255234 | 0.05868908663 |
| 223 | AAEL017425-RA | 0.5310337599 | 8.984801783 | 0.05910355873 |
| 224 | AAEL002293-RB | 0.3579125265 | 6.043052703 | 0.05922710658 |
| 225 | AAEL010464-RE | 0.3865226116 | 6.507030705 | 0.05940076651 |
| 226 | AAEL028665-RA | 0.5705474697 | 9.596374076 | 0.05945448408 |
| 227 | AAEL009898-RA | 0.322899464 | 5.427086482 | 0.05949775539 |
| 228 | AAEL028813-RA | 0.5717884569 | 9.598774008 | 0.059568905 |
| 229 | AAEL012069-RB | 0.4001964558 | 6.709028354 | 0.05965043441 |
| 230 | AAEL028980-RA | 0.5726765389 | 9.599771174 | 0.05965522808 |
| 231 | AAEL002158-RA | 0.4749492652 | 7.953162925 | 0.05971828688 |
| 232 | AAEL007078-RA | 0.3187834789 | 5.324263727 | 0.05987372062 |
| 233 | AAEL003393-RA | 0.5699858278 | 9.518181312 | 0.05988390104 |
| 234 | AAEL003675-RA | 0.5311163675 | 8.861430885 | 0.05993573435 |
| 235 | AAEL006509-RA | 0.6142654298 | 10.22616513 | 0.06006801395 |
| 236 | AAEL028907-RA | 0.5927440704 | 9.860296803 | 0.06011422194 |
| 237 | AAEL023078-RA | 0.4551951198 | 7.571382235 | 0.06012047809 |
| 238 | AAEL021717-RA | 0.4551951198 | 7.571382235 | 0.06012047809 |
| 239 | AAEL012740-RB | 0.7040284785 | 11.68706404 | 0.06023997779 |
| 240 | AAEL005558-RA | 0.3612932384 | 5.996035848 | 0.06025534996 |
| 241 | AAEL002121-RA | 0.4343721439 | 7.205865268 | 0.06028035882 |
| 242 | AAEL003946-RA | 0.3419852563 | 5.659581103 | 0.0604258955 |
| 243 | AAEL028730-RA | 0.6022407999 | 9.937048615 | 0.06060560064 |
| 244 | AAEL018668-RA | 0.7212843374 | 11.90020312 | 0.06061109465 |
| 245 | AAEL003210-RI | 0.361637425 | 5.941736967 | 0.06086392363 |
| 246 | AAEL006833-RD | 0.4797315467 | 7.880575175 | 0.06087519452 |
| 247 | AAEL021307-RA | 0.3078382517 | 5.053383766 | 0.06091725188 |
| 248 | AAEL013675-RA | 0.3567934179 | 5.846682698 | 0.06102493266 |
| 249 | AAEL009526-RA | 0.3532441814 | 5.782830631 | 0.06108499522 |
| 250 | AAEL008394-RA | 0.4216365934 | 6.900020563 | 0.06110657056 |
| 251 | AAEL028777-RA | 0.6281167372 | 10.26566671 | 0.06118616111 |
| 252 | AAEL003872-RA | 0.552139027 | 8.990663346 | 0.06141249046 |
| 253 | AAEL021035-RA | 0.334611691 | 5.440019258 | 0.06150928428 |
| 254 | AAEL008887-RA | 0.6352061709 | 10.31498885 | 0.06158088782 |
| 255 | AAEL017012-RI | 0.3667529621 | 5.946671161 | 0.06167365777 |
| 256 | AAEL023347-RA | 0.3195917171 | 5.176045976 | 0.06174437372 |
| 257 | AAEL005435-RB | 0.5612839923 | 9.082635021 | 0.06179748399 |
| 258 | AAEL023330-RB | 0.3169902636 | 5.103596561 | 0.06211115235 |
| 259 | AAEL000791-RA | 0.3982965314 | 6.411330419 | 0.06212385033 |
| 260 | AAEL004860-RA | 0.3272354763 | 5.261450092 | 0.06219492166 |
| 261 | AAEL008697-RB | 0.6488766553 | 10.42070998 | 0.06226798911 |
| 262 | AAEL015450-RA | 0.451095471 | 7.242018475 | 0.06228863852 |
| 263 | AAEL006928-RA | 0.4944952337 | 7.929758262 | 0.06235943359 |
| 264 | AAEL003423-RA | 0.5705304654 | 9.147125728 | 0.06237264933 |
| 265 | AAEL009981-RA | 0.3224347965 | 5.159614529 | 0.06249203205 |
| 266 | AAEL023500-RA | 0.3822367809 | 6.114521182 | 0.06251295392 |
| 267 | AAEL010059-RA | 0.3691196343 | 5.884750868 | 0.06272476824 |
| 268 | AAEL009185-RB | 0.5046433112 | 8.035914448 | 0.06279849225 |
| 269 | AAEL008025-RA | 0.3838889131 | 6.112986286 | 0.06279891613 |
| 270 | AAEL010299-RD | 0.3835569485 | 6.092441297 | 0.06295619929 |
| 271 | AAEL002789-RA | 0.3475124843 | 5.517294523 | 0.06298603108 |
| 272 | AAEL000709-RB | 0.3303258267 | 5.243724462 | 0.06299450498 |
| 273 | AAEL013068-RC | 0.4373630176 | 6.940980502 | 0.06301170525 |
| 274 | AAEL022304-RA | 0.5746372292 | 9.115267662 | 0.06304117998 |
| 275 | AAEL004065-RA | 0.4504200805 | 7.124248915 | 0.06322351813 |
| 276 | AAEL002118-RA | 0.4390894849 | 6.923985202 | 0.06341571683 |
| 277 | AAEL013876-RA | 0.518091259 | 8.146042877 | 0.06360035994 |
| 278 | AAEL001887-RD | 0.3444277977 | 5.413127921 | 0.06362823912 |
| 279 | AAEL028794-RA | 0.6294607258 | 9.888872529 | 0.06365343713 |
| 280 | AAEL003234-RA | 0.6959595611 | 10.92639908 | 0.06369523535 |
| 281 | AAEL023486-RA | 0.5433201024 | 8.518033241 | 0.06378468915 |
| 282 | AAEL009379-RB | 0.3580846854 | 5.595932407 | 0.06399017346 |
| 283 | AAEL004423-RA | 0.6657576304 | 10.39890992 | 0.06402186722 |
| 284 | AAEL012121-RA | 0.7150745571 | 11.16392421 | 0.06405225829 |
| 285 | AAEL012026-RD | 0.3894622196 | 6.07455056 | 0.0641137506 |
| 286 | AAEL009078-RA | 0.5713683419 | 8.906195327 | 0.06415403221 |
| 287 | AAEL001864-RA | 0.4373587088 | 6.814858564 | 0.06417722461 |
| 288 | AAEL014583-RE | 0.6847768661 | 10.6531395 | 0.06427934847 |
| 289 | AAEL013353-RF | 0.4528482493 | 7.042833315 | 0.06429915761 |
| 290 | AAEL005236-RA | 0.6938744158 | 10.75818183 | 0.06449736829 |
| 291 | AAEL011381-RA | 0.5257774725 | 8.131117063 | 0.06466239121 |
| 292 | AAEL005914-RA | 0.3986674021 | 6.161333099 | 0.06470473123 |
| 293 | AAEL024675-RA | 0.3407928799 | 5.259056706 | 0.0648011419 |
| 294 | AAEL006929-RB | 0.3299008454 | 5.086461962 | 0.06485860857 |
| 295 | AAEL019642-RA | 0.3695044159 | 5.682480069 | 0.06502520227 |
| 296 | AAEL008848-RA | 0.6435555505 | 9.876578582 | 0.06515976612 |
| 297 | AAEL004148-RA | 0.3750068746 | 5.749772085 | 0.06522117208 |
| 298 | AAEL019333-RA | 0.5596006623 | 8.560497903 | 0.06537010682 |
| 299 | AAEL005593-RB | 0.4115407053 | 6.284272775 | 0.06548740325 |
| 300 | AAEL000291-RA | 0.5875985814 | 8.955468256 | 0.06561338443 |
| 301 | AAEL007049-RA | 0.359618485 | 5.476623909 | 0.06566426525 |
| 302 | AAEL017245-RA | 0.4115473721 | 6.264918165 | 0.0656907818 |
| 303 | AAEL004472-RA | 0.4226623652 | 6.431081498 | 0.06572181759 |
| 304 | AAEL017395-RA | 0.3810465417 | 5.797531071 | 0.06572565753 |
| 305 | AAEL007054-RA | 0.5861194056 | 8.913735716 | 0.06575463132 |
| 306 | AAEL010823-RA | 0.648750712 | 9.863277775 | 0.06577435279 |
| 307 | AAEL001952-RA | 0.3934593623 | 5.979083556 | 0.06580596485 |
| 308 | AAEL001164-RA | 0.5375567663 | 8.165936585 | 0.06582916249 |
| 309 | AAEL025697-RA | 0.459857076 | 6.969194245 | 0.06598425297 |
| 310 | AAEL007023-RC | 0.4163996391 | 6.303315022 | 0.06606042021 |
| 311 | AAEL000713-RD | 0.4563080244 | 6.907024015 | 0.066064346 |
| 312 | AAEL009872-RA | 0.4511415434 | 6.822855366 | 0.06612210273 |
| 313 | AAEL012078-RA | 0.368890269 | 5.565720208 | 0.06627898192 |
| 314 | AAEL000258-RB | 0.34262913 | 5.167723437 | 0.06630175438 |
| 315 | AAEL009810-RB | 0.4038462065 | 6.08332926 | 0.06638572224 |
| 316 | AAEL017931-RA | 0.4266944988 | 6.415329135 | 0.06651170811 |
| 317 | AAEL019334-RA | 0.5937836125 | 8.915398528 | 0.06660202689 |
| 318 | AAEL027977-RA | 0.3921569055 | 5.882545215 | 0.0666644949 |
| 319 | AAEL006115-RA | 0.4499312298 | 6.745552513 | 0.06670042654 |
| 320 | AAEL002160-RA | 0.3678834597 | 5.504920614 | 0.06682811352 |
| 321 | AAEL012684-RA | 0.4305930484 | 6.42233306 | 0.06704620337 |
| 322 | AAEL005165-RD | 0.4729668291 | 7.044096823 | 0.06714371494 |
| 323 | AAEL007777-RA | 0.3797637989 | 5.654592736 | 0.06716023887 |
| 324 | AAEL026565-RB | 0.7038298863 | 10.46812953 | 0.06723549652 |
| 325 | AAEL004350-RA | 0.3402176219 | 5.058132991 | 0.06726150191 |
| 326 | AAEL009708-RD | 0.3754321189 | 5.57887877 | 0.06729526386 |
| 327 | AAEL002334-RA | 0.4377390177 | 6.494680538 | 0.06739962267 |
| 328 | AAEL026769-RA | 0.3686190993 | 5.466991924 | 0.0674263113 |
| 329 | AAEL027771-RA | 0.5546982203 | 8.222702675 | 0.06745935518 |
| 330 | AAEL002297-RA | 0.5632799375 | 8.346948503 | 0.06748333685 |
| 331 | AAEL022010-RA | 0.3846089934 | 5.69239912 | 0.06756535957 |
| 332 | AAEL009185-RA | 0.63510842 | 9.378811928 | 0.06771736387 |
| 333 | AAEL002825-RA | 0.5838943871 | 8.610897791 | 0.06780876992 |
| 334 | AAEL014821-RA | 0.3616040546 | 5.331679947 | 0.06782178567 |
| 335 | AAEL010754-RB | 0.7253387819 | 10.68400871 | 0.06789013393 |
| 336 | AAEL020753-RA | 0.5414959687 | 7.971943248 | 0.06792521621 |
| 337 | AAEL017573-RA | 0.3617359712 | 5.323525983 | 0.0679504472 |
| 338 | AAEL005931-RB | 0.350980748 | 5.1620002 | 0.06799316824 |
| 339 | AAEL026565-RA | 0.6049042385 | 8.891638192 | 0.06803068518 |
| 340 | AAEL011099-RA | 0.3468823545 | 5.09798119 | 0.06804308246 |
| 341 | AAEL000084-RA | 0.398433443 | 5.853047653 | 0.06807281721 |
| 342 | AAEL004855-RA | 0.7459552108 | 10.95578489 | 0.06808779271 |
| 343 | AAEL019332-RA | 0.6065940059 | 8.886386376 | 0.06826104338 |
| 344 | AAEL026904-RA | 0.5115289543 | 7.478710664 | 0.06839801368 |
| 345 | AAEL012218-RA | 0.3432876753 | 5.018033165 | 0.06841080239 |
| 346 | AAEL003957-RD | 0.5263733641 | 7.690980404 | 0.06844034655 |
| 347 | AAEL012860-RA | 0.3602684459 | 5.262569511 | 0.06845865791 |
| 348 | AAEL000221-RB | 0.3800880497 | 5.509447867 | 0.06898841025 |
| 349 | AAEL019669-RA | 0.5061819954 | 7.335025408 | 0.06900889462 |
| 350 | AAEL000175-RB | 0.3935257361 | 5.701741714 | 0.06901851325 |
| 351 | AAEL010017-RA | 0.4695412173 | 6.799651068 | 0.0690537224 |
| 352 | AAEL008871-RA | 0.5843349988 | 8.456394112 | 0.06909978308 |
| 353 | AAEL017087-RA | 0.5251629 | 7.592249676 | 0.06917092066 |
| 354 | AAEL019799-RD | 0.5178115085 | 7.48336897 | 0.06919497228 |
| 355 | AAEL019826-RG | 0.3663681477 | 5.29293331 | 0.06921835705 |
| 356 | AAEL012173-RA | 0.5928351686 | 8.562176028 | 0.0692388438 |
| 357 | AAEL017799-RA | 0.4576065675 | 6.574077677 | 0.06960772142 |
| 358 | AAEL006642-RA | 0.6342270373 | 9.102900922 | 0.06967306826 |
| 359 | AAEL009964-RA | 0.6701804603 | 9.603734886 | 0.06978331537 |
| 360 | AAEL004676-RA | 0.3581030882 | 5.131096786 | 0.06979074906 |
| 361 | AAEL024228-RA | 0.608837137 | 8.712840842 | 0.06987814285 |
| 362 | AAEL015061-RA | 0.427246997 | 6.111636798 | 0.06990713144 |
| 363 | AAEL000986-RB | 0.5478255219 | 7.820360929 | 0.07005118138 |
| 364 | AAEL005422-RA | 0.5291220372 | 7.535546384 | 0.07021681113 |
| 365 | AAEL017066-RA | 0.4942959713 | 7.038172563 | 0.07023072635 |
| 366 | AAEL000641-RA | 0.5137868857 | 7.30130819 | 0.07036915472 |
| 367 | AAEL000418-RA | 0.4438226782 | 6.300917762 | 0.07043778303 |
| 368 | AAEL011711-RA | 0.519569672 | 7.375017743 | 0.07044995552 |
| 369 | AAEL005517-RA | 0.6592933516 | 9.355164212 | 0.07047373372 |
| 370 | AAEL010801-RA | 0.7110767699 | 10.07595689 | 0.07057163681 |
| 371 | AAEL008490-RC | 0.5825511869 | 8.254697266 | 0.07057208377 |
| 372 | AAEL004197-RA | 0.9586329857 | 13.54276121 | 0.07078563748 |
| 373 | AAEL009645-RD | 0.4019861074 | 5.673304665 | 0.07085572363 |
| 374 | AAEL013913-RA | 0.7385912783 | 10.42244159 | 0.07086547544 |
| 375 | AAEL000679-RA | 0.4388947645 | 6.189736816 | 0.07090685397 |
| 376 | AAEL000180-RA | 0.3973386692 | 5.596694865 | 0.07099523535 |
| 377 | AAEL022051-RA | 0.4947569514 | 6.967110082 | 0.07101322436 |
| 378 | AAEL008785-RA | 0.6730947338 | 9.470362143 | 0.07107381151 |
| 379 | AAEL007868-RA | 0.7346936144 | 10.30268947 | 0.07131085687 |
| 380 | AAEL006510-RB | 0.3973295505 | 5.565728983 | 0.07138859109 |
| 381 | AAEL007494-RA | 0.4763769278 | 6.648154879 | 0.07165550991 |
| 382 | AAEL022285-RC | 0.6315393232 | 8.796123912 | 0.07179745642 |
| 383 | AAEL002813-RA | 0.708182371 | 9.854720875 | 0.07186224552 |
| 384 | AAEL017466-RA | 0.648856318 | 9.007055873 | 0.07203866914 |
| 385 | AAEL017545-RA | 0.4199404332 | 5.822562985 | 0.07212295243 |
| 386 | AAEL021316-RA | 0.37686557 | 5.215984598 | 0.07225204808 |
| 387 | AAEL014555-RA | 0.37686557 | 5.215984598 | 0.07225204808 |
| 388 | AAEL017007-RA | 0.5757325878 | 7.955323197 | 0.07237073511 |
| 389 | AAEL007162-RA | 0.5920918038 | 8.166815985 | 0.07249971163 |
| 390 | AAEL009414-RA | 0.5628048899 | 7.737217295 | 0.07273996173 |
| 391 | AAEL014953-RA | 0.5074650567 | 6.971557619 | 0.07279077137 |
| 392 | AAEL001856-RA | 0.3763585935 | 5.158780067 | 0.07295496001 |
| 393 | AAEL004195-RA | 0.4127172364 | 5.645261377 | 0.07310861425 |
| 394 | AAEL005221-RC | 0.4094445306 | 5.593252756 | 0.07320329484 |
| 395 | AAEL013144-RA | 0.4873965363 | 6.633742236 | 0.07347233566 |
| 396 | AAEL012819-RA | 0.3853835329 | 5.236261069 | 0.07359899131 |
| 397 | AAEL001946-RD | 0.3911842066 | 5.31372105 | 0.07361775354 |
| 398 | AAEL004059-RB | 0.4239833524 | 5.741696274 | 0.07384287364 |
| 399 | AAEL001865-RA | 0.6159426453 | 8.329400436 | 0.07394801704 |
| 400 | AAEL008002-RA | 0.6468893943 | 8.723055829 | 0.0741585755 |
| 401 | AAEL000638-RA | 0.7059717563 | 9.51888181 | 0.07416540833 |
| 402 | AAEL010056-RA | 0.4361881173 | 5.881169167 | 0.0741669054 |
| 403 | AAEL010611-RC | 0.6111826981 | 8.231367515 | 0.07425044465 |
| 404 | AAEL000102-RB | 0.4362491028 | 5.860849691 | 0.0744344465 |
| 405 | AAEL012552-RA | 0.388615375 | 5.209276891 | 0.07460063711 |
| 406 | AAEL001377-RA | 0.5499740829 | 7.36621065 | 0.07466173709 |
| 407 | AAEL000269-RA | 0.5502178689 | 7.360304185 | 0.07475477305 |
| 408 | AAEL009101-RA | 0.5289198007 | 7.058082008 | 0.07493817726 |
| 409 | AAEL009712-RA | 0.3969704514 | 5.293378682 | 0.07499377528 |
| 410 | AAEL005471-RA | 0.5430584452 | 7.237362632 | 0.07503540623 |
| 411 | AAEL006061-RA | 0.4302030586 | 5.731860439 | 0.07505469876 |
| 412 | AAEL007392-RB | 0.4947599611 | 6.577084322 | 0.07522481648 |
| 413 | AAEL006855-RC | 0.378528959 | 5.022934106 | 0.07536012836 |
| 414 | AAEL011746-RB | 0.5120482081 | 6.788063864 | 0.07543361676 |
| 415 | AAEL006829-RA | 0.5415543912 | 7.172186015 | 0.07550757749 |
| 416 | AAEL012950-RA | 0.6048052838 | 8.002085126 | 0.075580961 |
| 417 | AAEL018681-RA | 0.4898475807 | 6.46948989 | 0.07571656948 |
| 418 | AAEL005610-RA | 0.6867789615 | 9.051952141 | 0.07587081226 |
| 419 | AAEL024040-RA | 0.6868402547 | 9.052008402 | 0.07587711192 |
| 420 | AAEL023019-RA | 0.4428868432 | 5.832788188 | 0.07593055481 |
| 421 | AAEL018680-RA | 0.7112860486 | 9.366193326 | 0.07594185 |
| 422 | AAEL019487-RA | 0.4531917814 | 5.955814719 | 0.07609232369 |
| 423 | AAEL014889-RA | 0.5533964897 | 7.257450709 | 0.07625218715 |
| 424 | AAEL022204-RA | 0.4233978157 | 5.550801949 | 0.07627687307 |
| 425 | AAEL002328-RA | 0.5018967239 | 6.577999998 | 0.07629928916 |
| 426 | AAEL004071-RA | 0.6262142619 | 8.198509099 | 0.07638148038 |
| 427 | AAEL022285-RA | 0.6217349283 | 8.125460509 | 0.07651688511 |
| 428 | AAEL012994-RA | 0.4831824781 | 6.312528915 | 0.0765434083 |
| 429 | AAEL003011-RA | 0.6193872519 | 8.084476773 | 0.07661438944 |
| 430 | AAEL005150-RA | 0.395251289 | 5.156106665 | 0.07665692638 |
| 431 | AAEL013875-RB | 0.4913224859 | 6.394839302 | 0.07683109187 |
| 432 | AAEL012327-RA | 0.4164473969 | 5.417998457 | 0.07686369795 |
| 433 | AAEL012661-RB | 0.5713749537 | 7.420585553 | 0.07699863436 |
| 434 | AAEL002350-RA | 0.4406540616 | 5.718323512 | 0.07706000905 |
| 435 | AAEL004088-RH | 0.4861152228 | 6.301518728 | 0.07714254987 |
| 436 | AAEL013744-RA | 0.5517717182 | 7.131879105 | 0.07736694776 |
| 437 | AAEL001421-RC | 0.4539663534 | 5.866362324 | 0.07738464287 |
| 438 | AAEL022173-RB | 0.4325653148 | 5.583508961 | 0.0774719478 |
| 439 | AAEL004563-RA | 0.4214809287 | 5.438584289 | 0.07749828012 |
| 440 | AAEL009590-RB | 0.6038617329 | 7.780896898 | 0.07760824245 |
| 441 | AAEL008317-RA | 0.5659468491 | 7.289618576 | 0.07763737473 |
| 442 | AAEL000028-RA | 0.4957331273 | 6.383972342 | 0.07765276864 |
| 443 | AAEL010330-RA | 0.6255814577 | 8.042844353 | 0.07778112198 |
| 444 | AAEL002542-RA | 0.6274484884 | 8.061610531 | 0.07783165486 |
| 445 | AAEL013980-RC | 0.4558340086 | 5.844201988 | 0.07799764783 |
| 446 | AAEL012681-RA | 0.6320913459 | 8.103166045 | 0.07800547865 |
| 447 | AAEL002504-RB | 0.6890830601 | 8.824235018 | 0.07808983541 |
| 448 | AAEL006174-RA | 0.42151521 | 5.397384904 | 0.07809619241 |
| 449 | AAEL009506-RA | 0.6692957011 | 8.569774327 | 0.0780995713 |
| 450 | AAEL020447-RA | 0.6692972658 | 8.569775139 | 0.07809974648 |
| 451 | AAEL001916-RB | 0.4354955276 | 5.572406516 | 0.078152146 |
| 452 | AAEL001930-RD | 0.4477199777 | 5.725934473 | 0.07819159996 |
| 453 | AAEL007236-RA | 0.511488431 | 6.535249255 | 0.07826609377 |
| 454 | AAEL005269-RE | 0.6825449564 | 8.718331729 | 0.07828848197 |
| 455 | AAEL011315-RA | 0.4291456777 | 5.471503755 | 0.07843285812 |
| 456 | AAEL024853-RA | 0.4693887725 | 5.982702547 | 0.07845764833 |
| 457 | AAEL013960-RA | 0.6787975039 | 8.649488633 | 0.07847833931 |
| 458 | AAEL017525-RA | 0.6281720417 | 7.999471734 | 0.07852669058 |
| 459 | AAEL006117-RA | 0.4369399424 | 5.563369587 | 0.07853872289 |
| 460 | AAEL019748-RA | 0.5097734996 | 6.475951719 | 0.07871792776 |
| 461 | AAEL002687-RA | 0.4003726567 | 5.079139783 | 0.07882686317 |
| 462 | AAEL003428-RA | 0.4104457597 | 5.203623668 | 0.07887691076 |
| 463 | AAEL005946-RA | 0.6381731432 | 8.085847643 | 0.07892470541 |
| 464 | AAEL008517-RA | 0.4598384674 | 5.81918853 | 0.07902106369 |
| 465 | AAEL006070-RA | 0.7231384324 | 9.149127064 | 0.07903906322 |
| 466 | AAEL010087-RA | 0.4534025084 | 5.734416548 | 0.07906689453 |
| 467 | AAEL024472-RA | 0.5493316923 | 6.945930353 | 0.07908684141 |
| 468 | AAEL025594-RA | 0.4789420071 | 6.047167198 | 0.07920105257 |
| 469 | AAEL026018-RA | 0.412003473 | 5.196826969 | 0.07927981351 |
| 470 | AAEL013571-RD | 0.4249596938 | 5.356925943 | 0.07932902159 |
| 471 | AAEL003503-RA | 0.5432145062 | 6.843484063 | 0.07937689358 |
| 472 | AAEL006480-RA | 0.5112438945 | 6.4336557 | 0.07946398104 |
| 473 | AAEL007549-RA | 0.5302095381 | 6.671509386 | 0.07947370039 |
| 474 | AAEL013964-RA | 0.4534014085 | 5.699743291 | 0.07954768931 |
| 475 | AAEL010765-RA | 0.5796155498 | 7.277198067 | 0.07964817564 |
| 476 | AAEL023967-RA | 0.5151309293 | 6.465163676 | 0.0796779409 |
| 477 | AAEL008770-RA | 0.4419581901 | 5.537616339 | 0.07981018603 |
| 478 | AAEL017354-RA | 0.425068854 | 5.324026419 | 0.07983973416 |
| 479 | AAEL015584-RA | 0.4148444904 | 5.194456569 | 0.07986292404 |
| 480 | AAEL011871-RA | 0.7682687278 | 9.6180999 | 0.07987739115 |
| 481 | AAEL021894-RA | 0.4608925787 | 5.713297774 | 0.08067014829 |
| 482 | AAEL010400-RA | 0.5742592835 | 7.112428047 | 0.08074025912 |
| 483 | AAEL007945-RA | 0.5487411208 | 6.793727547 | 0.08077172907 |
| 484 | AAEL000469-RA | 0.4361871691 | 5.396712649 | 0.08082460519 |
| 485 | AAEL003418-RA | 0.4996408906 | 6.181087145 | 0.0808338208 |
| 486 | AAEL009066-RA | 0.6823111585 | 8.440275175 | 0.08083991864 |
| 487 | AAEL019799-RA | 0.6804941071 | 8.41482125 | 0.08086851603 |
| 488 | AAEL010230-RA | 0.6522279082 | 8.05067072 | 0.08101535026 |
| 489 | AAEL005830-RA | 0.4282283795 | 5.27925128 | 0.08111536215 |
| 490 | AAEL009278-RA | 0.5863467753 | 7.220398928 | 0.08120697777 |
| 491 | AAEL001779-RA | 0.5659526388 | 6.963341354 | 0.08127601535 |
| 492 | AAEL017646-RA | 0.5660577423 | 6.960594787 | 0.08132318567 |
| 493 | AAEL009275-RB | 0.5918165012 | 7.277221525 | 0.08132451365 |
| 494 | AAEL015064-RB | 0.6535227603 | 8.022938353 | 0.08145678448 |
| 495 | AAEL022847-RA | 0.5803570089 | 7.115107615 | 0.08156686311 |
| 496 | AAEL000200-RA | 0.5944002649 | 7.281806367 | 0.08162813387 |
| 497 | AAEL004873-RB | 0.4877367552 | 5.971786006 | 0.08167351521 |
| 498 | AAEL004440-RA | 0.4509705598 | 5.512631985 | 0.08180675964 |
| 499 | AAEL002797-RA | 0.5934768973 | 7.247152069 | 0.08189105067 |
| 500 | AAEL010840-RC | 0.6219455854 | 7.586259458 | 0.08198316823 |
| 501 | AAEL004691-RA | 0.559393396 | 6.807653451 | 0.08217125034 |
| 502 | AAEL008601-RA | 0.5164018457 | 6.283660586 | 0.0821816899 |
| 503 | AAEL013359-RE | 0.5727368264 | 6.967658133 | 0.08219932945 |
| 504 | AAEL010787-RA | 0.6905382904 | 8.399049719 | 0.08221624035 |
| 505 | AAEL003746-RA | 0.547239339 | 6.644304592 | 0.0823621692 |
| 506 | AAEL001636-RA | 0.4553514329 | 5.523647517 | 0.08243672889 |
| 507 | AAEL011288-RA | 0.6393356826 | 7.753295848 | 0.08245985903 |
| 508 | AAEL005474-RA | 0.4808440227 | 5.813218597 | 0.08271562728 |
| 509 | AAEL000568-RB | 0.4361505652 | 5.269757614 | 0.08276482471 |
| 510 | AAEL010821-RA | 0.7374453883 | 8.895811441 | 0.08289804625 |
| 511 | AAEL006420-RA | 0.4428164587 | 5.336472128 | 0.08297925071 |
| 512 | AAEL019799-RJ | 0.5837730492 | 7.031960259 | 0.08301711438 |
| 513 | AAEL010826-RB | 0.5060798485 | 6.095823744 | 0.08302074825 |
| 514 | AAEL006721-RI | 0.6441796285 | 7.758333435 | 0.08303067069 |
| 515 | AAEL013236-RA | 0.4548597401 | 5.474545324 | 0.08308630456 |
| 516 | AAEL009608-RC | 0.4163069862 | 5.008717977 | 0.08311647574 |
| 517 | AAEL003206-RA | 0.4681579322 | 5.629616187 | 0.08315983126 |
| 518 | AAEL007881-RA | 0.5512709007 | 6.628841795 | 0.08316247661 |
| 519 | AAEL003431-RA | 0.456637327 | 5.49043872 | 0.0831695517 |
| 520 | AAEL004430-RA | 0.4778471819 | 5.741696727 | 0.08322403718 |
| 521 | AAEL000746-RA | 0.4605984723 | 5.532120959 | 0.08325893011 |
| 522 | AAEL002399-RA | 0.5305249039 | 6.37093761 | 0.08327265724 |
| 523 | AAEL000486-RA | 0.5179052847 | 6.213291374 | 0.08335441772 |
| 524 | AAEL008868-RA | 0.6554109894 | 7.861426988 | 0.0833704861 |
| 525 | AAEL001102-RA | 0.4623297084 | 5.542767236 | 0.08341135189 |
| 526 | AAEL001091-RA | 0.5279810893 | 6.324104286 | 0.08348709405 |
| 527 | AAEL024066-RA | 0.5897959717 | 7.057498033 | 0.08357012201 |
| 528 | AAEL007452-RB | 0.5415643835 | 6.471785453 | 0.08368083081 |
| 529 | AAEL006514-RD | 0.4538564882 | 5.415170113 | 0.08381204629 |
| 530 | AAEL003871-RA | 0.4409670592 | 5.257172763 | 0.08387912649 |
| 531 | AAEL006186-RA | 0.4310118599 | 5.136485614 | 0.08391182072 |
| 532 | AAEL002503-RA | 0.4531060805 | 5.383186324 | 0.08417061071 |
| 533 | AAEL004434-RA | 0.5366580161 | 6.366838048 | 0.08428956604 |
| 534 | AAEL004308-RA | 0.4810613699 | 5.703936645 | 0.08433848407 |
| 535 | AAEL000424-RA | 0.4305061354 | 5.103380504 | 0.08435705217 |
| 536 | AAEL001356-RA | 0.514501347 | 6.093013529 | 0.08444119558 |
| 537 | AAEL023497-RA | 0.4234748718 | 5.002124787 | 0.08465899789 |
| 538 | AAEL010611-RD | 0.5808206816 | 6.834178384 | 0.08498763845 |
| 539 | AAEL023037-RB | 0.5101691258 | 5.993645665 | 0.08511833269 |
| 540 | AAEL027357-RA | 0.4726912178 | 5.550529179 | 0.08516146884 |
| 541 | AAEL014505-RA | 0.6368772163 | 7.476339971 | 0.08518569498 |
| 542 | AAEL004433-RA | 0.433347122 | 5.083714011 | 0.08524223058 |
| 543 | AAEL002720-RA | 0.4527983687 | 5.310790306 | 0.08526007292 |
| 544 | AAEL002827-RA | 0.8865838828 | 10.38056715 | 0.08540803891 |
| 545 | AAEL020452-RA | 0.4645926252 | 5.436311331 | 0.08546100415 |
| 546 | AAEL017072-RA | 0.4778739306 | 5.578355522 | 0.08566573586 |
| 547 | AAEL000947-RA | 0.4873747239 | 5.686768889 | 0.08570327605 |
| 548 | AAEL010145-RC | 0.527955249 | 6.154664203 | 0.08578132479 |
| 549 | AAEL011128-RA | 0.6678167397 | 7.784050271 | 0.08579296336 |
| 550 | AAEL017089-RA | 0.4749246033 | 5.534777489 | 0.08580735256 |
| 551 | AAEL012264-RA | 0.4438917024 | 5.167852289 | 0.0858948123 |
| 552 | AAEL011081-RA | 0.4695177817 | 5.461352521 | 0.08597097145 |
| 553 | AAEL004805-RA | 0.5029528414 | 5.840705133 | 0.08611166458 |
| 554 | AAEL009604-RH | 0.4943742576 | 5.734401511 | 0.08621200602 |
| 555 | AAEL028652-RA | 0.6047792485 | 7.003137188 | 0.08635833231 |
| 556 | AAEL022285-RB | 0.6001652476 | 6.938125952 | 0.08650250107 |
| 557 | AAEL005069-RA | 0.5329153661 | 6.155859783 | 0.08657041987 |
| 558 | AAEL013613-RB | 0.7560617859 | 8.721643223 | 0.0866879975 |
| 559 | AAEL022547-RA | 0.5622996791 | 6.464120823 | 0.08698780461 |
| 560 | AAEL000138-RA | 0.6836677685 | 7.856150783 | 0.08702324936 |
| 561 | AAEL004427-RA | 0.4864589802 | 5.583304261 | 0.0871274352 |
| 562 | AAEL028874-RA | 0.5154081474 | 5.904944922 | 0.08728415831 |
| 563 | AAEL028915-RA | 0.5154081474 | 5.904944922 | 0.08728415831 |
| 564 | AAEL007355-RA | 0.4681902079 | 5.363934095 | 0.08728485466 |
| 565 | AAEL010456-RB | 0.5197339291 | 5.953616713 | 0.08729717652 |
| 566 | AAEL020646-RG | 0.4382769747 | 5.017271829 | 0.0873536435 |
| 567 | AAEL004450-RA | 0.5140424857 | 5.869898275 | 0.08757263951 |
| 568 | AAEL007778-RA | 0.4484377526 | 5.118099522 | 0.08761802125 |
| 569 | AAEL003135-RD | 0.4394057162 | 5.011650821 | 0.08767684179 |
| 570 | AAEL008166-RA | 0.8218019212 | 9.363139782 | 0.08776990843 |
| 571 | AAEL003019-RC | 0.4784109233 | 5.432869391 | 0.08805860934 |
| 572 | AAEL010608-RB | 0.6573824498 | 7.464310608 | 0.08807008234 |
| 573 | AAEL009389-RA | 0.4899230444 | 5.558252612 | 0.08814335702 |
| 574 | AAEL017121-RA | 0.4592606653 | 5.205387172 | 0.08822795503 |
| 575 | AAEL005038-RB | 0.470909534 | 5.325804113 | 0.0884203632 |
| 576 | AAEL008369-RD | 0.4948240898 | 5.584597904 | 0.08860514191 |
| 577 | AAEL027903-RA | 0.55332198 | 6.234127281 | 0.08875692699 |
| 578 | AAEL012897-RA | 0.7709652117 | 8.672116037 | 0.08890162544 |
| 579 | AAEL019568-RB | 0.4881159114 | 5.489326466 | 0.08892091123 |
| 580 | AAEL022810-RA | 0.9699002329 | 10.90306887 | 0.08895662724 |
| 581 | AAEL027639-RA | 0.4639310834 | 5.211352816 | 0.08902315766 |
| 582 | AAEL003103-RB | 0.6839279652 | 7.679516509 | 0.08905872712 |
| 583 | AAEL003873-RB | 0.5363709993 | 6.014488034 | 0.08917982649 |
| 584 | AAEL004234-RA | 0.4906921632 | 5.501282198 | 0.08919596297 |
| 585 | AAEL001896-RB | 0.5097711413 | 5.714983126 | 0.08919906324 |
| 586 | AAEL012095-RA | 0.5082586066 | 5.693372335 | 0.08927197743 |
| 587 | AAEL004527-RA | 0.4484192892 | 5.013300299 | 0.08944592633 |
| 588 | AAEL000410-RA | 0.5602865157 | 6.240752384 | 0.08977868071 |
| 589 | AAEL002464-RA | 0.5936901373 | 6.610002137 | 0.08981693576 |
| 590 | AAEL006576-RA | 0.6628074038 | 7.367300692 | 0.08996611263 |
| 591 | AAEL003203-RF | 0.5778733382 | 6.422547459 | 0.089975721 |
| 592 | AAEL004034-RA | 0.4607496273 | 5.119473929 | 0.08999940887 |
| 593 | AAEL004739-RA | 0.5782823597 | 6.419656445 | 0.09007995437 |
| 594 | AAEL001005-RA | 0.6303460824 | 6.977657381 | 0.09033778072 |
| 595 | AAEL005766-RA | 0.8695143455 | 9.622016215 | 0.09036716693 |
| 596 | AAEL021140-RA | 0.573908334 | 6.349146438 | 0.09039141553 |
| 597 | AAEL017290-RA | 0.6486659604 | 7.166424077 | 0.09051459326 |
| 598 | AAEL012924-RB | 0.5035046245 | 5.551810344 | 0.09069197132 |
| 599 | AAEL009808-RA | 0.6133493648 | 6.739291132 | 0.09101096137 |
| 600 | AAEL019482-RA | 0.6777869155 | 7.44161847 | 0.09108057853 |
| 601 | AAEL025872-RA | 0.6824281434 | 7.487327512 | 0.09114442266 |
| 602 | AAEL004631-RA | 0.7473298924 | 8.185542722 | 0.09129875902 |
| 603 | AAEL004603-RA | 0.4609663972 | 5.046480079 | 0.09134414284 |
| 604 | AAEL002337-RA | 0.5951585516 | 6.512511612 | 0.09138694671 |
| 605 | AAEL003497-RA | 0.5553336445 | 6.056354897 | 0.09169436962 |
| 606 | AAEL000847-RA | 0.4605634348 | 5.006049018 | 0.09200138335 |
| 607 | AAEL010819-RA | 0.662699172 | 7.19622167 | 0.09208987748 |
| 608 | AAEL025082-RA | 0.7431178598 | 8.061669557 | 0.09217915154 |
| 609 | AAEL003483-RA | 0.5805961001 | 6.294324603 | 0.09224120724 |
| 610 | AAEL019408-RA | 0.6916424949 | 7.493945737 | 0.09229350187 |
| 611 | AAEL007019-RA | 0.5936661269 | 6.428173277 | 0.09235378409 |
| 612 | AAEL011657-RA | 0.5021572044 | 5.433862383 | 0.09241257306 |
| 613 | AAEL004567-RA | 0.4689757332 | 5.071816281 | 0.09246701915 |
| 614 | AAEL004445-RA | 0.4747982153 | 5.131224372 | 0.09253117401 |
| 615 | AAEL024147-RC | 0.4956056019 | 5.354375366 | 0.09256086248 |
| 616 | AAEL014760-RD | 0.4638640537 | 5.010288371 | 0.09258230652 |
| 617 | AAEL002418-RA | 0.4791357849 | 5.168864299 | 0.09269653006 |
| 618 | AAEL004321-RA | 0.7598238428 | 8.188416434 | 0.09279252575 |
| 619 | AAEL001341-RA | 0.4759713619 | 5.126207223 | 0.09285058938 |
| 620 | AAEL001314-RA | 0.5897587806 | 6.350099563 | 0.09287394232 |
| 621 | AAEL001854-RA | 0.6827375158 | 7.326651866 | 0.09318547247 |
| 622 | AAEL001607-RB | 0.4844761446 | 5.193747856 | 0.09328064396 |
| 623 | AAEL002309-RA | 0.7365411073 | 7.881568814 | 0.09345107867 |
| 624 | AAEL010724-RA | 0.5817297518 | 6.210816054 | 0.09366398018 |
| 625 | AAEL005991-RA | 0.5134151631 | 5.479883008 | 0.09369089856 |
| 626 | AAEL010146-RC | 0.5978527381 | 6.376355637 | 0.09376088351 |
| 627 | AAEL018658-RA | 0.5772801427 | 6.136354999 | 0.09407541494 |
| 628 | AAEL002587-RA | 0.6779610411 | 7.205450012 | 0.09409003462 |
| 629 | AAEL028938-RA | 0.7692585902 | 8.166564131 | 0.09419611208 |
| 630 | AAEL012974-RA | 0.4886737119 | 5.187610064 | 0.09420016267 |
| 631 | AAEL014715-RB | 0.5011284984 | 5.312115814 | 0.09433689247 |
| 632 | AAEL005213-RC | 0.4994373558 | 5.292656855 | 0.09436420489 |
| 633 | AAEL010743-RB | 0.476276348 | 5.038863818 | 0.09452058345 |
| 634 | AAEL017273-RA | 0.5374388818 | 5.683377951 | 0.09456328373 |
| 635 | AAEL003103-RA | 0.4915992577 | 5.192098529 | 0.09468218966 |
| 636 | AAEL023349-RA | 0.6043888996 | 6.375325905 | 0.09480125543 |
| 637 | AAEL005798-RA | 0.6551068603 | 6.907505425 | 0.09483986185 |
| 638 | AAEL000941-RA | 0.6267570518 | 6.606860216 | 0.0948645849 |
| 639 | AAEL014799-RB | 0.5700639092 | 6.007022196 | 0.09489958428 |
| 640 | AAEL022901-RA | 0.4938762184 | 5.19845982 | 0.09500433504 |
| 641 | AAEL008274-RA | 0.5341304068 | 5.619557972 | 0.09504847346 |
| 642 | AAEL009317-RB | 0.5910059157 | 6.211473833 | 0.09514745317 |
| 643 | AAEL012276-RA | 0.508406232 | 5.343320392 | 0.09514799688 |
| 644 | AAEL008291-RA | 0.4812388317 | 5.042816147 | 0.09543057246 |
| 645 | AAEL014583-RD | 0.6706625588 | 7.019182364 | 0.09554710564 |
| 646 | AAEL012035-RA | 0.7056832899 | 7.379283608 | 0.09563032503 |
| 647 | AAEL001432-RA | 0.5845460393 | 6.107669771 | 0.09570688352 |
| 648 | AAEL008740-RA | 0.492240943 | 5.136131622 | 0.09583884901 |
| 649 | AAEL013461-RA | 0.4835685091 | 5.044590847 | 0.09585881666 |
| 650 | AAEL022609-RA | 0.5631716152 | 5.872615979 | 0.09589791283 |
| 651 | AAEL013495-RB | 0.4868485153 | 5.058161025 | 0.09625010214 |
| 652 | AAEL003888-RB | 0.6604012286 | 6.86119972 | 0.09625156759 |
| 653 | AAEL008719-RA | 0.5767234 | 5.989997849 | 0.09628106964 |
| 654 | AAEL020822-RA | 0.5179247552 | 5.375333256 | 0.09635212007 |
| 655 | AAEL017187-RA | 0.4981172929 | 5.15066829 | 0.09670925497 |
| 656 | AAEL008248-RA | 0.5150583008 | 5.312320515 | 0.09695542643 |
| 657 | AAEL002610-RA | 0.5606445284 | 5.771141058 | 0.09714621818 |
| 658 | AAEL011184-RC | 0.9643718059 | 9.918663503 | 0.09722799907 |
| 659 | AAEL003675-RB | 0.8423394376 | 8.658066046 | 0.09728956018 |
| 660 | AAEL011468-RA | 0.6025024185 | 6.182734529 | 0.09744918138 |
| 661 | AAEL011197-RC | 0.7878200432 | 8.072233936 | 0.09759628492 |
| 662 | AAEL008364-RB | 0.4890474066 | 5.00734198 | 0.09766606886 |
| 663 | AAEL017469-RA | 0.5638925213 | 5.754876552 | 0.09798516375 |
| 664 | AAEL018672-RA | 0.6397594138 | 6.512678365 | 0.09823292014 |
| 665 | AAEL003492-RA | 0.7458239915 | 7.588974098 | 0.09827731415 |
| 666 | AAEL002184-RA | 0.541475979 | 5.496603647 | 0.09851101039 |
| 667 | AAEL008787-RA | 0.6964552459 | 7.059176263 | 0.09865956308 |
| 668 | AAEL007773-RA | 0.5254799516 | 5.323057559 | 0.09871769107 |
| 669 | AAEL006922-RA | 0.6696457169 | 6.776966223 | 0.09881201925 |
| 670 | AAEL014913-RC | 0.7216753449 | 7.293365453 | 0.09894956581 |
| 671 | AAEL007022-RA | 0.547408777 | 5.525926188 | 0.09906190535 |
| 672 | AAEL024549-RA | 0.9908943951 | 9.982772658 | 0.09926043887 |
| 673 | AAEL011394-RA | 0.5307212781 | 5.339594156 | 0.09939356113 |
| 674 | AAEL020429-RA | 0.8364129335 | 8.396905096 | 0.09960966855 |
| 675 | AAEL010143-RA | 0.8005907115 | 8.031818208 | 0.09967739443 |
| 676 | AAEL011022-RA | 0.5790554081 | 5.809224026 | 0.09967861551 |
| 677 | AAEL012943-RA | 0.5440436837 | 5.456277531 | 0.09970967947 |
| 678 | AAEL027227-RA | 0.5895493555 | 5.903964619 | 0.09985651906 |
| 679 | AAEL001247-RA | 0.6099818338 | 6.102059466 | 0.09996327261 |
| 680 | AAEL000208-RA | 0.5955829968 | 5.954809354 | 0.1000171393 |
| 681 | AAEL014799-RA | 0.5077002272 | 5.071869346 | 0.1001012038 |
| 682 | AAEL000703-RA | 0.7402887769 | 7.392965196 | 0.1001342164 |
| 683 | AAEL022617-RA | 0.5121461667 | 5.108266601 | 0.1002583081 |
| 684 | AAEL000179-RA | 0.6693066192 | 6.66584546 | 0.1004083613 |
| 685 | AAEL013256-RA | 0.5235213664 | 5.21084619 | 0.1004676299 |
| 686 | AAEL001969-RA | 0.7675825475 | 7.632181081 | 0.1005718469 |
| 687 | AAEL004438-RA | 0.5450034155 | 5.413432617 | 0.1006761244 |
| 688 | AAEL007787-RC | 0.5157125949 | 5.121310585 | 0.1006993398 |
| 689 | AAEL005457-RA | 0.5629015391 | 5.578214335 | 0.1009107046 |
| 690 | AAEL010690-RA | 0.7329430958 | 7.260657868 | 0.1009472019 |
| 691 | AAEL001061-RB | 0.67097391 | 6.64049049 | 0.1010428237 |
| 692 | AAEL011197-RD | 0.74682135 | 7.38667788 | 0.1011038199 |
| 693 | AAEL008553-RA | 0.5304081398 | 5.236197675 | 0.1012964316 |
| 694 | AAEL001484-RC | 0.5503371952 | 5.419019247 | 0.1015566046 |
| 695 | AAEL014605-RA | 0.5271130742 | 5.180130054 | 0.1017567259 |
| 696 | AAEL020590-RA | 0.6249752676 | 6.128965885 | 0.1019707532 |
| 697 | AAEL021859-RA | 0.546501299 | 5.34109455 | 0.1023200945 |
| 698 | AAEL018687-RA | 0.8597473649 | 8.397225736 | 0.1023846913 |
| 699 | AAEL026741-RA | 0.6868926646 | 6.701314977 | 0.1025011758 |
| 700 | AAEL002851-RA | 0.8891130076 | 8.664250623 | 0.1026185698 |
| 701 | AAEL010900-RA | 0.5299344037 | 5.16003965 | 0.1026996767 |
| 702 | AAEL013527-RA | 0.548473281 | 5.336000337 | 0.10278734 |
| 703 | AAEL000431-RA | 0.7132174286 | 6.924296702 | 0.1030021473 |
| 704 | AAEL003993-RA | 0.6473686272 | 6.284210893 | 0.1030151022 |
| 705 | AAEL004450-RB | 0.5533038046 | 5.353376871 | 0.1033560345 |
| 706 | AAEL000950-RA | 0.5301201258 | 5.127233353 | 0.1033930171 |
| 707 | AAEL006895-RC | 0.5506249731 | 5.313351622 | 0.1036304412 |
| 708 | AAEL001020-RB | 0.5319629319 | 5.129659137 | 0.1037033685 |
| 709 | AAEL005742-RA | 0.5698817999 | 5.492401178 | 0.1037582255 |
| 710 | AAEL002764-RC | 0.6361578194 | 6.130285792 | 0.1037729465 |
| 711 | AAEL024598-RB | 0.6544862713 | 6.284665405 | 0.1041401935 |
| 712 | AAEL008723-RA | 0.6622033204 | 6.346187741 | 0.1043466326 |
| 713 | AAEL008405-RA | 0.5925583643 | 5.673098528 | 0.1044505681 |
| 714 | AAEL004404-RA | 0.5632858337 | 5.391425332 | 0.1044780923 |
| 715 | AAEL004988-RA | 0.8956653499 | 8.560571297 | 0.1046268197 |
| 716 | AAEL002175-RA | 0.5844603493 | 5.584914538 | 0.1046498286 |
| 717 | AAEL022565-RA | 0.541298217 | 5.168727105 | 0.1047256328 |
| 718 | AAEL009345-RA | 0.6099006884 | 5.823775625 | 0.1047259935 |
| 719 | AAEL007441-RA | 0.5692349747 | 5.422513438 | 0.1049762221 |
| 720 | AAEL013987-RA | 0.6995235552 | 6.659731245 | 0.1050378055 |
| 721 | AAEL005524-RA | 0.5730005388 | 5.449654953 | 0.1051443704 |
| 722 | AAEL014452-RA | 0.6580140335 | 6.243445344 | 0.1053927755 |
| 723 | AAEL002296-RA | 0.6927189917 | 6.561251432 | 0.1055772666 |
| 724 | AAEL010037-RB | 0.6234350914 | 5.890912235 | 0.1058299745 |
| 725 | AAEL009160-RD | 0.5322758331 | 5.02618284 | 0.1059006109 |
| 726 | AAEL004755-RA | 0.551185414 | 5.201854085 | 0.1059594147 |
| 727 | AAEL004865-RA | 0.7073255277 | 6.675148865 | 0.105964008 |
| 728 | AAEL019717-RB | 0.9502654627 | 8.939578229 | 0.1062986909 |
| 729 | AAEL002436-RD | 0.6968683246 | 6.551899602 | 0.1063612642 |
| 730 | AAEL013989-RA | 0.6840738875 | 6.421821912 | 0.1065233351 |
| 731 | AAEL009074-RE | 0.6084935579 | 5.690898749 | 0.1069239824 |
| 732 | AAEL001159-RA | 0.586696409 | 5.483536994 | 0.1069923317 |
| 733 | AAEL017030-RA | 0.8153980914 | 7.61880128 | 0.1070244598 |
| 734 | AAEL025498-RA | 0.5764498835 | 5.384336076 | 0.1070605318 |
| 735 | AAEL009029-RA | 0.6280918683 | 5.865962944 | 0.1070739577 |
| 736 | AAEL006834-RA | 0.7602733669 | 7.099822524 | 0.1070834326 |
| 737 | AAEL004042-RE | 0.5844524952 | 5.4559065 | 0.1071228943 |
| 738 | AAEL008228-RA | 0.5380031962 | 5.017401041 | 0.1072274654 |
| 739 | AAEL013709-RA | 0.7673147794 | 7.148883968 | 0.1073335059 |
| 740 | AAEL014943-RA | 0.7391784303 | 6.88461646 | 0.1073666826 |
| 741 | AAEL002886-RB | 0.5665296569 | 5.273003107 | 0.1074396592 |
| 742 | AAEL007820-RC | 0.6137582899 | 5.709068291 | 0.1075058589 |
| 743 | AAEL004294-RA | 0.7350691798 | 6.832352252 | 0.107586546 |
| 744 | AAEL008303-RA | 0.666710288 | 6.183987265 | 0.1078123643 |
| 745 | AAEL003039-RA | 0.6224121317 | 5.76356255 | 0.1079908696 |
| 746 | AAEL002600-RB | 0.653826778 | 6.051706658 | 0.1080400646 |
| 747 | AAEL011200-RA | 0.5517255296 | 5.106199392 | 0.1080501342 |
| 748 | AAEL008041-RB | 0.6162987431 | 5.696585883 | 0.1081873873 |
| 749 | AAEL006946-RA | 0.5697308164 | 5.253971533 | 0.108438124 |
| 750 | AAEL020485-RA | 0.5465489491 | 5.036708399 | 0.1085131212 |
| 751 | AAEL002183-RA | 0.5475628149 | 5.039110354 | 0.1086625965 |
| 752 | AAEL021861-RA | 0.6025564612 | 5.537840486 | 0.1088071176 |
| 753 | AAEL011665-RA | 0.5670367637 | 5.206742716 | 0.1089043178 |
| 754 | AAEL017251-RA | 0.5929833078 | 5.442231693 | 0.1089595852 |
| 755 | AAEL007880-RA | 0.6079805035 | 5.56033106 | 0.1093425008 |
| 756 | AAEL002161-RA | 0.5586769287 | 5.101871228 | 0.1095043179 |
| 757 | AAEL007822-RB | 0.6053041538 | 5.527435549 | 0.1095090388 |
| 758 | AAEL028675-RA | 0.9647866072 | 8.807585146 | 0.1095404235 |
| 759 | AAEL019387-RA | 0.9651116536 | 8.806849517 | 0.1095864817 |
| 760 | AAEL012117-RE | 0.6107789896 | 5.565486452 | 0.1097440439 |
| 761 | AAEL005699-RA | 0.5661813174 | 5.150753024 | 0.1099220473 |
| 762 | AAEL019720-RF | 0.5624314683 | 5.114073152 | 0.1099772044 |
| 763 | AAEL028735-RA | 0.9069333888 | 8.244921551 | 0.1099990319 |
| 764 | AAEL001264-RA | 0.5698213674 | 5.167551957 | 0.1102691124 |
| 765 | AAEL025674-RA | 0.9460561345 | 8.546888397 | 0.1106901238 |
| 766 | AAEL007662-RF | 0.60771649 | 5.48499158 | 0.1107962485 |
| 767 | AAEL014717-RA | 0.6120161151 | 5.517380972 | 0.1109251143 |
| 768 | AAEL027639-RB | 0.5809856736 | 5.213292866 | 0.1114431298 |
| 769 | AAEL009859-RA | 0.7226883905 | 6.477853681 | 0.1115629383 |
| 770 | AAEL007065-RD | 0.6592204257 | 5.906360238 | 0.1116119572 |
| 771 | AAEL025769-RA | 0.6844368023 | 6.130905631 | 0.1116371452 |
| 772 | AAEL003750-RA | 0.8046722839 | 7.194921695 | 0.1118389217 |
| 773 | AAEL006342-RC | 0.5773571436 | 5.15481529 | 0.1120034591 |
| 774 | AAEL002501-RB | 0.6152812719 | 5.48777396 | 0.1121185523 |
| 775 | AAEL026008-RA | 0.5926570017 | 5.278521451 | 0.112277085 |
| 776 | AAEL004987-RD | 0.9191604716 | 8.186251027 | 0.1122810024 |
| 777 | AAEL007286-RA | 0.5883830574 | 5.235397349 | 0.1123855589 |
| 778 | AAEL006224-RA | 0.6145151494 | 5.461314324 | 0.1125214761 |
| 779 | AAEL001963-RA | 0.6749695995 | 5.991021194 | 0.1126635306 |
| 780 | AAEL001113-RA | 0.7267455679 | 6.428329811 | 0.1130535597 |
| 781 | AAEL027157-RA | 0.6804526237 | 6.014458354 | 0.1131361435 |
| 782 | AAEL007153-RA | 0.6458107788 | 5.706971547 | 0.113161731 |
| 783 | AAEL002221-RA | 0.6079003749 | 5.365802144 | 0.1132916121 |
| 784 | AAEL004887-RA | 0.8903234462 | 7.857955812 | 0.1133021701 |
| 785 | AAEL004120-RA | 0.6206825215 | 5.476286448 | 0.1133400394 |
| 786 | AAEL003046-RB | 0.8276260352 | 7.283840199 | 0.1136249578 |
| 787 | AAEL006102-RA | 0.7785340811 | 6.845948379 | 0.1137218743 |
| 788 | AAEL014511-RE | 0.6619176163 | 5.802869885 | 0.1140672856 |
| 789 | AAEL007707-RB | 0.7122964945 | 6.238359067 | 0.1141801052 |
| 790 | AAEL002860-RA | 0.7342385833 | 6.429059779 | 0.114206215 |
| 791 | AAEL006647-RA | 0.5797396541 | 5.074400674 | 0.1142479066 |
| 792 | AAEL017551-RA | 0.750343423 | 6.567246587 | 0.1142554057 |
| 793 | AAEL004288-RA | 0.5773219451 | 5.051893315 | 0.1142783327 |
| 794 | AAEL005008-RE | 0.6198087502 | 5.42085657 | 0.1143377882 |
| 795 | AAEL011113-RA | 0.5888222197 | 5.149165036 | 0.1143529515 |
| 796 | AAEL007546-RA | 0.7051613498 | 6.166098039 | 0.1143610344 |
| 797 | AAEL025856-RA | 0.8245753343 | 7.208749097 | 0.1143853564 |
| 798 | AAEL017083-RA | 0.6394950635 | 5.575259076 | 0.1147023044 |
| 799 | AAEL007010-RA | 0.6579160236 | 5.71709865 | 0.1150786551 |
| 800 | AAEL003835-RF | 0.5996988579 | 5.210222527 | 0.1151004309 |
| 801 | AAEL003161-RA | 0.6950163383 | 6.038072667 | 0.1151056598 |
| 802 | AAEL026751-RA | 0.6238519985 | 5.410329316 | 0.1153075833 |
| 803 | AAEL025900-RA | 0.8308202818 | 7.1905902 | 0.1155427105 |
| 804 | AAEL011789-RD | 0.7119587586 | 6.158294859 | 0.1156097223 |
| 805 | AAEL022497-RC | 0.665589128 | 5.756581746 | 0.1156222837 |
| 806 | AAEL025553-RB | 0.6912230939 | 5.977735535 | 0.1156329332 |
| 807 | AAEL000454-RB | 0.9745020208 | 8.427303923 | 0.1156362734 |
| 808 | AAEL008672-RA | 0.6400171278 | 5.49546637 | 0.1164627503 |
| 809 | AAEL019418-RA | 0.6309970275 | 5.408989567 | 0.1166570983 |
| 810 | AAEL013119-RA | 0.6049918774 | 5.1742409 | 0.116923794 |
| 811 | AAEL010159-RA | 0.6552438482 | 5.59348172 | 0.1171441834 |
| 812 | AAEL025665-RA | 0.609200203 | 5.200266949 | 0.1171478712 |
| 813 | AAEL011090-RA | 0.6615836274 | 5.631572057 | 0.1174776103 |
| 814 | AAEL012621-RD | 0.6862694845 | 5.826573624 | 0.1177826848 |
| 815 | AAEL026843-RA | 0.6614899113 | 5.614452572 | 0.1178191289 |
| 816 | AAEL012094-RA | 0.7033217961 | 5.961811495 | 0.1179711564 |
| 817 | AAEL001107-RA | 0.7067908359 | 5.980490084 | 0.118182762 |
| 818 | AAEL017116-RD | 0.6176143236 | 5.214573684 | 0.1184400415 |
| 819 | AAEL006952-RA | 0.7407278976 | 6.231825608 | 0.118862103 |
| 820 | AAEL009685-RA | 0.6760789312 | 5.685103391 | 0.1189211321 |
| 821 | AAEL006158-RA | 0.9870870053 | 8.276383268 | 0.119265502 |
| 822 | AAEL026825-RA | 0.6692256943 | 5.608648079 | 0.1193203219 |
| 823 | AAEL008963-RA | 0.6817871026 | 5.712718098 | 0.1193454833 |
| 824 | AAEL014960-RB | 0.6809320342 | 5.703224745 | 0.1193942137 |
| 825 | AAEL017345-RA | 0.6935159413 | 5.806676872 | 0.1194342231 |
| 826 | AAEL008502-RA | 0.6213087137 | 5.201616178 | 0.1194453209 |
| 827 | AAEL005292-RA | 0.8199368489 | 6.861704468 | 0.1194946318 |
| 828 | AAEL007752-RA | 0.9195859053 | 7.69458153 | 0.1195108404 |
| 829 | AAEL006872-RA | 0.7981504712 | 6.664323146 | 0.1197646713 |
| 830 | AAEL012113-RC | 0.6846815889 | 5.704405457 | 0.1200268098 |
| 831 | AAEL007173-RG | 0.6361790026 | 5.300075988 | 0.1200320531 |
| 832 | AAEL004060-RB | 0.9717737934 | 8.085091444 | 0.1201932965 |
| 833 | AAEL000019-RA | 0.8832950127 | 7.329953736 | 0.1205048551 |
| 834 | AAEL002259-RA | 0.8316855771 | 6.898539911 | 0.1205596529 |
| 835 | AAEL004521-RA | 0.7646455763 | 6.327732607 | 0.1208403742 |
| 836 | AAEL006951-RB | 0.6953699168 | 5.754085754 | 0.1208480281 |
| 837 | AAEL004338-RA | 0.8362258785 | 6.919099763 | 0.1208576126 |
| 838 | AAEL012311-RB | 0.8259204737 | 6.8228608 | 0.1210519308 |
| 839 | AAEL000758-RB | 0.688880481 | 5.690776555 | 0.121052105 |
| 840 | AAEL001946-RA | 0.7643677681 | 6.313548594 | 0.1210678522 |
| 841 | AAEL004307-RB | 0.6123599754 | 5.047661215 | 0.1213155854 |
| 842 | AAEL006976-RB | 0.7200744572 | 5.929794175 | 0.121433297 |
| 843 | AAEL007431-RA | 0.7015124102 | 5.767274482 | 0.1216367302 |
| 844 | AAEL001838-RA | 0.7031956014 | 5.759347559 | 0.1220963997 |
| 845 | AAEL023719-RA | 0.765832906 | 6.271652263 | 0.1221102309 |
| 846 | AAEL006885-RE | 0.6752361495 | 5.526445478 | 0.1221827216 |
| 847 | AAEL011830-RE | 0.7104895929 | 5.814942418 | 0.1221834271 |
| 848 | AAEL013528-RA | 0.7791834366 | 6.342745193 | 0.1228464037 |
| 849 | AAEL008854-RA | 0.6987038623 | 5.684259335 | 0.1229190684 |
| 850 | AAEL013656-RA | 0.8565092394 | 6.954829431 | 0.1231531625 |
| 851 | AAEL010032-RA | 0.6965547531 | 5.644451498 | 0.1234052154 |
| 852 | AAEL000235-RA | 0.6302594189 | 5.096339891 | 0.1236690316 |
| 853 | AAEL007702-RA | 0.622619902 | 5.031933804 | 0.1237337227 |
| 854 | AAEL002411-RC | 0.8204446064 | 6.627529103 | 0.123793437 |
| 855 | AAEL024063-RA | 0.627542105 | 5.057982271 | 0.1240696529 |
| 856 | AAEL021342-RA | 0.7688548003 | 6.194749195 | 0.1241139514 |
| 857 | AAEL010602-RA | 0.7180107821 | 5.768417717 | 0.1244727441 |
| 858 | AAEL009863-RG | 0.7976268782 | 6.403034115 | 0.1245701434 |
| 859 | AAEL012313-RB | 0.6715658515 | 5.388294314 | 0.1246342186 |
| 860 | AAEL009423-RA | 0.6250344126 | 5.010009212 | 0.1247571384 |
| 861 | AAEL028771-RA | 0.635192338 | 5.085572807 | 0.1249008444 |
| 862 | AAEL028865-RA | 0.6353828065 | 5.086585178 | 0.124913431 |
| 863 | AAEL028996-RA | 0.6355345465 | 5.087591334 | 0.1249185528 |
| 864 | AAEL028724-RA | 0.6351969514 | 5.084776853 | 0.1249213033 |
| 865 | AAEL008128-RA | 0.6615547834 | 5.295388395 | 0.1249303609 |
| 866 | AAEL007184-RA | 0.9229927983 | 7.386100002 | 0.1249634852 |
| 867 | AAEL021191-RA | 0.6478665641 | 5.179760677 | 0.125076544 |
| 868 | AAEL012731-RA | 0.7095955138 | 5.666067164 | 0.125235987 |
| 869 | AAEL020998-RB | 0.6717580698 | 5.345898481 | 0.1256585908 |
| 870 | AAEL021185-RA | 0.797300606 | 6.338697217 | 0.1257830401 |
| 871 | AAEL004067-RA | 0.7042053517 | 5.594729905 | 0.1258694099 |
| 872 | AAEL026480-RA | 0.8324109426 | 6.594267689 | 0.126232507 |
| 873 | AAEL011527-RA | 0.6392183121 | 5.057938777 | 0.126379211 |
| 874 | AAEL014393-RB | 0.654397787 | 5.175278631 | 0.1264468705 |
| 875 | AAEL007293-RE | 0.7883079996 | 6.224665343 | 0.1266426315 |
| 876 | AAEL011992-RD | 0.6921943893 | 5.456415651 | 0.1268588087 |
| 877 | AAEL005651-RA | 0.7558540099 | 5.95584828 | 0.1269095474 |
| 878 | AAEL005361-RA | 0.9275348543 | 7.301845079 | 0.1270274628 |
| 879 | AAEL004242-RA | 0.709509537 | 5.581226055 | 0.1271243146 |
| 880 | AAEL010989-RA | 0.6663559459 | 5.240701852 | 0.1271501346 |
| 881 | AAEL026606-RA | 0.8282455943 | 6.489384296 | 0.1276308439 |
| 882 | AAEL012610-RA | 0.6553225803 | 5.132241227 | 0.1276874082 |
| 883 | AAEL002933-RA | 0.6574285594 | 5.14506712 | 0.1277784223 |
| 884 | AAEL017691-RA | 0.8228829859 | 6.437579443 | 0.1278249058 |
| 885 | AAEL006711-RI | 0.6404572453 | 5.008114289 | 0.1278839117 |
| 886 | AAEL013661-RA | 0.7916429664 | 6.178660933 | 0.1281253293 |
| 887 | AAEL027020-RA | 0.8711726036 | 6.796041863 | 0.1281882338 |
| 888 | AAEL004297-RJ | 0.8023733939 | 6.24176783 | 0.1285490611 |
| 889 | AAEL017664-RA | 0.7240669122 | 5.611769129 | 0.1290264969 |
| 890 | AAEL012827-RA | 0.6911268813 | 5.351838435 | 0.1291382185 |
| 891 | AAEL013530-RA | 0.8082860789 | 6.250257875 | 0.1293204369 |
| 892 | AAEL004534-RB | 0.6510376582 | 5.032327065 | 0.1293710941 |
| 893 | AAEL000472-RA | 0.6664181352 | 5.14941045 | 0.1294163947 |
| 894 | AAEL004957-RD | 0.6895158271 | 5.322515411 | 0.1295469855 |
| 895 | AAEL002155-RA | 0.6482834213 | 5.001631029 | 0.1296144033 |
| 896 | AAEL010697-RD | 0.7329522827 | 5.648196605 | 0.1297674876 |
| 897 | AAEL025391-RD | 0.6915041686 | 5.327142972 | 0.1298076985 |
| 898 | AAEL021666-RB | 0.7063802898 | 5.4371672 | 0.1299169703 |
| 899 | AAEL002597-RB | 0.6519948031 | 5.013335511 | 0.1300520984 |
| 900 | AAEL002527-RA | 0.7473990817 | 5.740556841 | 0.1301962688 |
| 901 | AAEL007160-RA | 0.755510545 | 5.784703068 | 0.1306048964 |
| 902 | AAEL012429-RA | 0.7061587902 | 5.405397702 | 0.1306395624 |
| 903 | AAEL022842-RA | 0.9529380146 | 7.293078049 | 0.1306633507 |
| 904 | AAEL011972-RA | 0.8038536587 | 6.146931996 | 0.1307731498 |
| 905 | AAEL017340-RA | 0.6717507939 | 5.13543374 | 0.1308070219 |
| 906 | AAEL011758-RA | 0.7206059837 | 5.507637851 | 0.1308375756 |
| 907 | AAEL003552-RA | 0.6929491687 | 5.291330455 | 0.1309593446 |
| 908 | AAEL000663-RA | 0.6942922963 | 5.292872057 | 0.131174963 |
| 909 | AAEL008692-RA | 0.7483979423 | 5.69335871 | 0.1314510433 |
| 910 | AAEL008367-RA | 0.7013275055 | 5.331485528 | 0.1315444827 |
| 911 | AAEL007001-RA | 0.7138377367 | 5.423547176 | 0.1316182405 |
| 912 | AAEL001769-RA | 0.7349286538 | 5.583085039 | 0.1316348665 |
| 913 | AAEL002993-RA | 0.7173394929 | 5.438694562 | 0.1318955284 |
| 914 | AAEL021617-RA | 0.7173394929 | 5.438694562 | 0.1318955284 |
| 915 | AAEL028739-RA | 0.7514703523 | 5.688511025 | 0.1321031723 |
| 916 | AAEL009604-RG | 0.7939156569 | 5.980666981 | 0.1327470096 |
| 917 | AAEL000931-RA | 0.774387657 | 5.826316376 | 0.1329120506 |
| 918 | AAEL020754-RA | 0.8281216317 | 6.230427499 | 0.1329156999 |
| 919 | AAEL026423-RA | 0.9343602049 | 7.024442106 | 0.1330155749 |
| 920 | AAEL023603-RA | 0.8467002765 | 6.346840954 | 0.1334049936 |
| 921 | AAEL005515-RI | 0.8417467534 | 6.303626105 | 0.1335337375 |
| 922 | AAEL007024-RA | 0.8219182569 | 6.149101799 | 0.1336647666 |
| 923 | AAEL008167-RC | 0.7216766575 | 5.396400966 | 0.1337329568 |
| 924 | AAEL007108-RA | 0.7233660834 | 5.404310874 | 0.1338498285 |
| 925 | AAEL005238-RA | 0.7010256428 | 5.227905792 | 0.1340930137 |
| 926 | AAEL011973-RA | 0.7170044294 | 5.322550425 | 0.134710688 |
| 927 | AAEL009059-RC | 0.7765302951 | 5.758691005 | 0.1348449317 |
| 928 | AAEL013952-RB | 0.6817815054 | 5.05214718 | 0.1349488606 |
| 929 | AAEL007290-RF | 0.6790080777 | 5.03002751 | 0.1349909272 |
| 930 | AAEL013074-RB | 0.7362957264 | 5.451976527 | 0.1350511549 |
| 931 | AAEL002593-RA | 0.7714793054 | 5.70878953 | 0.1351388594 |
| 932 | AAEL000753-RA | 0.7543507888 | 5.579868703 | 0.1351914944 |
| 933 | AAEL004221-RA | 0.7620158573 | 5.62999055 | 0.1353494025 |
| 934 | AAEL008064-RA | 0.7070356211 | 5.218461113 | 0.1354873795 |
| 935 | AAEL003076-RA | 0.8879105018 | 6.529424799 | 0.1359860216 |
| 936 | AAEL007439-RB | 0.8822148409 | 6.482490156 | 0.1360919677 |
| 937 | AAEL013612-RD | 0.9181202554 | 6.739108847 | 0.1362376356 |
| 938 | AAEL023576-RA | 0.7374064192 | 5.404470931 | 0.1364437756 |
| 939 | AAEL011302-RI | 0.6999254279 | 5.115775985 | 0.1368170596 |
| 940 | AAEL013199-RA | 0.7313866988 | 5.337070039 | 0.1370389921 |
| 941 | AAEL022005-RA | 0.7271891187 | 5.298606612 | 0.1372415754 |
| 942 | AAEL003453-RA | 0.8127231019 | 5.906077358 | 0.1376079338 |
| 943 | AAEL001354-RA | 0.8040580412 | 5.839487112 | 0.1376932641 |
| 944 | AAEL000111-RA | 0.7904593651 | 5.740685039 | 0.1376942577 |
| 945 | AAEL008849-RA | 0.7470781365 | 5.425277327 | 0.1377032162 |
| 946 | AAEL007717-RB | 0.7583979133 | 5.486642611 | 0.1382262281 |
| 947 | AAEL028013-RA | 0.7406652742 | 5.357187672 | 0.1382563613 |
| 948 | AAEL006977-RA | 0.8339020611 | 6.022882656 | 0.1384556381 |
| 949 | AAEL013620-RA | 0.7228865741 | 5.217891063 | 0.1385399897 |
| 950 | AAEL004973-RA | 0.704954765 | 5.081731402 | 0.1387233423 |
| 951 | AAEL013224-RA | 0.7711157926 | 5.554732121 | 0.1388214185 |
| 952 | AAEL008594-RA | 0.6980414099 | 5.021841833 | 0.1390010743 |
| 953 | AAEL007206-RA | 0.6991536439 | 5.019164856 | 0.1392968081 |
| 954 | AAEL007444-RB | 0.9196750972 | 6.600347227 | 0.1393373811 |
| 955 | AAEL007883-RD | 0.8573288632 | 6.14054527 | 0.1396177091 |
| 956 | AAEL011478-RD | 0.8399835323 | 5.992343755 | 0.1401761258 |
| 957 | AAEL015432-RB | 0.8407625831 | 5.997474786 | 0.1401860972 |
| 958 | AAEL002523-RA | 0.8013845018 | 5.71550728 | 0.1402123141 |
| 959 | AAEL001215-RA | 0.7039835274 | 5.019080562 | 0.140261452 |
| 960 | AAEL002023-RA | 0.8116284914 | 5.777819747 | 0.1404731416 |
| 961 | AAEL006962-RB | 0.7919739271 | 5.631979828 | 0.1406208742 |
| 962 | AAEL010244-RE | 0.7748791226 | 5.510252429 | 0.1406249773 |
| 963 | AAEL027050-RA | 0.707300624 | 5.028993376 | 0.1406445726 |
| 964 | AAEL012237-RA | 0.739391806 | 5.251645686 | 0.1407924011 |
| 965 | AAEL012713-RA | 0.7192797195 | 5.105179939 | 0.1408921386 |
| 966 | AAEL004081-RA | 0.726640809 | 5.156568785 | 0.140915566 |
| 967 | AAEL004930-RA | 0.9344522765 | 6.614175331 | 0.1412802397 |
| 968 | AAEL023493-RA | 0.7829767918 | 5.536286425 | 0.1414263518 |
| 969 | AAEL000245-RA | 0.8879190646 | 6.276366042 | 0.1414702486 |
| 970 | AAEL017315-RB | 0.7617105967 | 5.378578377 | 0.1416193171 |
| 971 | AAEL009733-RA | 0.8058825207 | 5.684723352 | 0.1417628389 |
| 972 | AAEL012419-RA | 0.7298551573 | 5.140601933 | 0.14197854 |
| 973 | AAEL014318-RA | 0.9468575053 | 6.661606585 | 0.1421365092 |
| 974 | AAEL001544-RC | 0.7773927639 | 5.459185783 | 0.1424008625 |
| 975 | AAEL004873-RA | 0.7934221792 | 5.567564663 | 0.1425079415 |
| 976 | AAEL001103-RA | 0.7532435787 | 5.273156765 | 0.1428449053 |
| 977 | AAEL023587-RA | 0.8584783094 | 5.983194423 | 0.1434816001 |
| 978 | AAEL013407-RA | 0.881969015 | 6.144843966 | 0.1435299285 |
| 979 | AAEL011276-RB | 0.8090019697 | 5.634734896 | 0.1435740962 |
| 980 | AAEL002137-RD | 0.7214435942 | 5.022877425 | 0.1436315349 |
| 981 | AAEL009287-RB | 0.795246103 | 5.536534252 | 0.1436360847 |
| 982 | AAEL002595-RB | 0.8539877634 | 5.937663761 | 0.1438255512 |
| 983 | AAEL001623-RA | 0.7344635719 | 5.104815752 | 0.1438766074 |
| 984 | AAEL014843-RB | 0.9726230324 | 6.745480272 | 0.1441888484 |
| 985 | AAEL008620-RA | 0.731433868 | 5.070685771 | 0.1442475241 |
| 986 | AAEL005866-RA | 0.8963537993 | 6.203132552 | 0.1445001847 |
| 987 | AAEL004523-RA | 0.8237482162 | 5.698036135 | 0.1445670397 |
| 988 | AAEL017075-RC | 0.9072823474 | 6.272568945 | 0.1446428657 |
| 989 | AAEL001052-RA | 0.9162608591 | 6.332631446 | 0.1446888023 |
| 990 | AAEL011708-RA | 0.9003681782 | 6.195614641 | 0.1453234635 |
| 991 | AAEL011832-RA | 0.7467571089 | 5.134785491 | 0.1454310234 |
| 992 | AAEL019990-RB | 0.8997534339 | 6.183157209 | 0.1455168296 |
| 993 | AAEL014642-RB | 0.9402089752 | 6.459433622 | 0.1455559466 |
| 994 | AAEL011752-RA | 0.8521024534 | 5.792672316 | 0.1471000614 |
| 995 | AAEL004846-RD | 0.801764875 | 5.448105995 | 0.1471639641 |
| 996 | AAEL010269-RA | 0.9162420385 | 6.223379908 | 0.1472257924 |
| 997 | AAEL008738-RA | 0.8879479519 | 6.026529936 | 0.1473398392 |
| 998 | AAEL011102-RB | 0.9097125965 | 6.145327296 | 0.1480332215 |
| 999 | AAEL024500-RA | 0.7587708309 | 5.116403067 | 0.1483016136 |
| 1000 | AAEL021444-RA | 0.9384413852 | 6.321902819 | 0.1484428679 |
| 1001 | AAEL026365-RA | 0.9188787132 | 6.187992691 | 0.1484938265 |
| 1002 | AAEL002781-RB | 0.7539312732 | 5.0728842 | 0.1486198469 |
| 1003 | AAEL028021-RD | 0.807530056 | 5.409615599 | 0.1492767908 |
| 1004 | AAEL019430-RC | 0.7709682182 | 5.156570799 | 0.149511807 |
| 1005 | AAEL023294-RA | 0.8034001987 | 5.358539147 | 0.1499289595 |
| 1006 | AAEL009539-RA | 0.9798843071 | 6.52350479 | 0.1502082605 |
| 1007 | AAEL012570-RA | 0.8041690312 | 5.349090396 | 0.1503375288 |
| 1008 | AAEL013138-RA | 0.7833730094 | 5.202008954 | 0.1505904769 |
| 1009 | AAEL000944-RA | 0.7698408906 | 5.11149957 | 0.1506095971 |
| 1010 | AAEL010467-RE | 0.8612382312 | 5.714061016 | 0.1507226172 |
| 1011 | AAEL013071-RA | 0.7736910475 | 5.132639037 | 0.1507394231 |
| 1012 | AAEL001352-RA | 0.8607944807 | 5.707949426 | 0.1508062557 |
| 1013 | AAEL026158-RA | 0.856251655 | 5.675962282 | 0.150855769 |
| 1014 | AAEL026538-RA | 0.8141568376 | 5.394208798 | 0.1509316506 |
| 1015 | AAEL015238-RA | 0.9545088145 | 6.315235301 | 0.1511438243 |
| 1016 | AAEL023187-RD | 0.8190220484 | 5.41800701 | 0.1511666646 |
| 1017 | AAEL011184-RA | 0.8059653406 | 5.323358422 | 0.1514016673 |
| 1018 | AAEL012904-RA | 0.9765936823 | 6.429653265 | 0.1518890121 |
| 1019 | AAEL026069-RF | 0.8485468748 | 5.586554888 | 0.1518909045 |
| 1020 | AAEL017447-RE | 0.8264573368 | 5.439255536 | 0.151943098 |
| 1021 | AAEL002551-RB | 0.8007705644 | 5.263424935 | 0.1521386881 |
| 1022 | AAEL009026-RF | 0.769828367 | 5.016704034 | 0.1534530165 |
| 1023 | AAEL024070-RA | 0.7845869354 | 5.109682512 | 0.1535490578 |
| 1024 | AAEL026700-RB | 0.7851207218 | 5.104262176 | 0.1538166918 |
| 1025 | AAEL013132-RE | 0.8146173117 | 5.294796159 | 0.1538524406 |
| 1026 | AAEL026215-RD | 0.8988975228 | 5.826148707 | 0.1542867455 |
| 1027 | AAEL022931-RA | 0.7798136696 | 5.038550199 | 0.1547694553 |
| 1028 | AAEL014959-RC | 0.8981769561 | 5.802781351 | 0.1547838703 |
| 1029 | AAEL000676-RA | 0.9060221267 | 5.847790374 | 0.154934098 |
| 1030 | AAEL002938-RA | 0.8140182982 | 5.230562288 | 0.1556273023 |
| 1031 | AAEL001061-RC | 0.8887336814 | 5.674699793 | 0.1566133388 |
| 1032 | AAEL002411-RD | 0.8801187648 | 5.618584416 | 0.1566442185 |
| 1033 | AAEL025431-RB | 0.8097428228 | 5.166537346 | 0.1567283402 |
| 1034 | AAEL004902-RA | 0.959407788 | 6.116770495 | 0.156848747 |
| 1035 | AAEL018336-RA | 0.9134274027 | 5.798358368 | 0.1575320711 |
| 1036 | AAEL005593-RA | 0.8990028151 | 5.702159801 | 0.1576600528 |
| 1037 | AAEL004054-RA | 0.9421972953 | 5.965916983 | 0.1579300044 |
| 1038 | AAEL017342-RA | 0.8768466439 | 5.544559939 | 0.158145399 |
| 1039 | AAEL026825-RB | 0.9450307053 | 5.964515726 | 0.1584421517 |
| 1040 | AAEL025888-RA | 0.9558218646 | 6.024185393 | 0.1586640852 |
| 1041 | AAEL011206-RA | 0.9280314832 | 5.843611393 | 0.1588112934 |
| 1042 | AAEL001134-RA | 0.9209756354 | 5.784088711 | 0.1592257106 |
| 1043 | AAEL005052-RB | 0.8992870995 | 5.634900167 | 0.1595923748 |
| 1044 | AAEL009627-RA | 0.8685181302 | 5.433437821 | 0.1598468886 |
| 1045 | AAEL009214-RA | 0.8875952837 | 5.546232986 | 0.160035701 |
| 1046 | AAEL011564-RB | 0.8057005552 | 5.023746993 | 0.1603784101 |
| 1047 | AAEL005046-RB | 0.8616027101 | 5.367716915 | 0.1605156762 |
| 1048 | AAEL017513-RA | 0.8705740627 | 5.40830884 | 0.1609697391 |
| 1049 | AAEL004118-RA | 0.8896405398 | 5.50786268 | 0.1615219172 |
| 1050 | AAEL019767-RB | 0.8594487778 | 5.307655202 | 0.1619262641 |
| 1051 | AAEL003858-RA | 0.8246897318 | 5.09167684 | 0.1619681998 |
| 1052 | AAEL027575-RA | 0.9317522182 | 5.747684255 | 0.1621091516 |
| 1053 | AAEL011881-RA | 0.8305278792 | 5.098769697 | 0.1628878982 |
| 1054 | AAEL027938-RI | 0.991455427 | 6.068253766 | 0.1633839759 |
| 1055 | AAEL017132-RD | 0.8535496272 | 5.176308001 | 0.1648954481 |
| 1056 | AAEL011105-RA | 0.8462527839 | 5.122161227 | 0.1652140076 |
| 1057 | AAEL002865-RA | 0.8625131299 | 5.210807128 | 0.1655239023 |
| 1058 | AAEL007342-RA | 0.911498246 | 5.455087017 | 0.1670914219 |
| 1059 | AAEL000770-RB | 0.9404718163 | 5.626126803 | 0.1671615037 |
| 1060 | AAEL026310-RA | 0.8946663116 | 5.346279913 | 0.1673437093 |
| 1061 | AAEL003180-RA | 0.8512065673 | 5.079791351 | 0.1675672303 |
| 1062 | AAEL024358-RA | 0.8848987213 | 5.275644035 | 0.1677328333 |
| 1063 | AAEL024482-RA | 0.8803091261 | 5.23292372 | 0.1682251019 |
| 1064 | AAEL002629-RB | 0.8688466818 | 5.149138911 | 0.1687363066 |
| 1065 | AAEL017419-RA | 0.9282066336 | 5.483740623 | 0.1692652329 |
| 1066 | AAEL021926-RA | 0.8545327047 | 5.030805926 | 0.169860002 |
| 1067 | AAEL004467-RA | 0.9202141031 | 5.401820258 | 0.1703525958 |
| 1068 | AAEL009387-RA | 0.867903627 | 5.067174281 | 0.1712796085 |
| 1069 | AAEL005567-RC | 0.9056902248 | 5.247871376 | 0.1725823977 |
| 1070 | AAEL007371-RB | 0.9379323836 | 5.432851788 | 0.1726408929 |
| 1071 | AAEL004419-RA | 0.9562103113 | 5.528093798 | 0.1729728811 |
| 1072 | AAEL021211-RC | 0.9361162429 | 5.377413167 | 0.1740830049 |
| 1073 | AAEL004484-RA | 0.918841707 | 5.248193122 | 0.175077724 |
| 1074 | AAEL014078-RB | 0.9543774361 | 5.446255709 | 0.1752355172 |
| 1075 | AAEL001605-RA | 0.8837587225 | 5.022299144 | 0.175966962 |
| 1076 | AAEL005414-RA | 0.9081554915 | 5.149554715 | 0.1763561205 |
| 1077 | AAEL013327-RA | 0.9380103324 | 5.310009648 | 0.1766494591 |
| 1078 | AAEL020739-RA | 0.9772241829 | 5.520982892 | 0.1770018495 |
| 1079 | AAEL012671-RC | 0.9564311306 | 5.379439629 | 0.1777938218 |
| 1080 | AAEL020524-RA | 0.9812959854 | 5.408706131 | 0.1814289706 |
| 1081 | AAEL009317-RA | 0.9957185416 | 5.478758779 | 0.1817416283 |
| 1082 | AAEL013536-RD | 0.9607778475 | 5.285621144 | 0.1817719851 |
| 1083 | AAEL019988-RA | 0.9901598099 | 5.392447742 | 0.1836197321 |
| 1084 | AAEL023449-RA | 0.9479597434 | 5.116563636 | 0.1852727359 |
| 1085 | AAEL007555-RA | 0.9608473892 | 5.154226488 | 0.1864193185 |
| 1086 | AAEL007322-RA | 0.9671453834 | 5.172025164 | 0.1869954907 |
| 1087 | AAEL019559-RA | 0.967109565 | 5.163975441 | 0.1872800473 |
| 1088 | AAEL008672-RB | 0.9617636488 | 5.107314949 | 0.1883110124 |
| 1089 | AAEL004294-RB | 0.9809452575 | 5.152022307 | 0.1904000408 |
| 1090 | AAEL008171-RA | 0.9709497391 | 5.00898817 | 0.1938414918 |
| 1091 | AAEL000622-RC | 0.9966566934 | 5.006815584 | 0.1990599967 |

## Table S4: Validation candidate genes full list for the PRJNA659517 transcriptome. The rank order (GSV ID) of the VectorBase genes (ID) was based on the standard deviation (SD). TPM avrg: TPM average.

| **GSV ID** | **ID** | **SD** | **TPM avrg** |
| --- | --- | --- | --- |
| 1 | AAEL006259-RA | 8.527251681 | 8.217347699 |
| 2 | AAEL017262-RA | 8.476450384 | 5.566228549 |
| 3 | AAEL013535-RA | 7.326451716 | 5.166652068 |
| 4 | AAEL003888-RC | 4.972236262 | 5.129493208 |
| 5 | AAEL023015-RA | 4.89721388 | 5.157223232 |
| 6 | AAEL006579-RA | 4.418938336 | 5.511723393 |
| 7 | AAEL004223-RA | 4.204042765 | 10.28595041 |
| 8 | AAEL004292-RA | 3.880707628 | 5.658267115 |
| 9 | AAEL019799-RB | 3.678006944 | 5.27662199 |
| 10 | AAEL020963-RA | 3.669554077 | 7.903826685 |
| 11 | AAEL008441-RA | 3.4976042 | 7.310460187 |
| 12 | AAEL027311-RA | 3.438665668 | 6.585553447 |
| 13 | AAEL011741-RE | 3.411577136 | 5.850746722 |
| 14 | AAEL003049-RA | 3.398253702 | 5.985697185 |
| 15 | AAEL004249-RA | 3.397924288 | 6.471788977 |
| 16 | AAEL026267-RA | 3.382249843 | 7.122032664 |
| 17 | AAEL022665-RA | 3.310223751 | 7.16978194 |
| 18 | AAEL018152-RD | 3.231685453 | 5.071686075 |
| 19 | AAEL026093-RA | 3.172985947 | 6.529294633 |
| 20 | AAEL017402-RA | 3.152563406 | 5.718066505 |
| 21 | AAEL021930-RA | 3.149772766 | 6.285396077 |
| 22 | AAEL026093-RB | 3.065261817 | 5.77535134 |
| 23 | AAEL025608-RA | 3.02962108 | 8.179878257 |
| 24 | AAEL014231-RB | 2.994158612 | 5.539003428 |
| 25 | AAEL001319-RB | 2.985645368 | 6.831245885 |
| 26 | AAEL009081-RA | 2.980474986 | 5.385214298 |
| 27 | AAEL003041-RA | 2.912873051 | 5.124298488 |
| 28 | AAEL013766-RB | 2.870300595 | 6.179366578 |
| 29 | AAEL012704-RC | 2.842665457 | 5.265637788 |
| 30 | AAEL003259-RA | 2.805341056 | 8.258998329 |
| 31 | AAEL001683-RA | 2.794334015 | 5.198965607 |
| 32 | AAEL017293-RA | 2.76260754 | 8.300468946 |
| 33 | AAEL012110-RB | 2.761715134 | 5.835515746 |
| 34 | AAEL005772-RA | 2.752587836 | 8.206562894 |
| 35 | AAEL002811-RA | 2.730943896 | 5.199640067 |
| 36 | AAEL017114-RA | 2.688424141 | 7.213471558 |
| 37 | AAEL014893-RA | 2.686913516 | 5.096228161 |
| 38 | AAEL004585-RA | 2.67994592 | 5.822368293 |
| 39 | AAEL008587-RA | 2.665446515 | 5.448655948 |
| 40 | AAEL010523-RB | 2.662537325 | 5.106973669 |
| 41 | AAEL007034-RA | 2.660769844 | 5.317060812 |
| 42 | AAEL008285-RA | 2.641396219 | 5.991857017 |
| 43 | AAEL001284-RA | 2.640761692 | 7.261556028 |
| 44 | AAEL026044-RA | 2.639499514 | 5.265874416 |
| 45 | AAEL003425-RB | 2.636336409 | 5.547893779 |
| 46 | AAEL020593-RA | 2.634020411 | 6.686642315 |
| 47 | AAEL006993-RN | 2.619435715 | 5.21171434 |
| 48 | AAEL007986-RA | 2.607067506 | 5.949648561 |
| 49 | AAEL026304-RA | 2.585856102 | 9.470996516 |
| 50 | AAEL002055-RA | 2.580696655 | 6.280634135 |
| 51 | AAEL005692-RE | 2.578604785 | 5.31979959 |
| 52 | AAEL007776-RA | 2.57706332 | 5.862261231 |
| 53 | AAEL005699-RB | 2.562823795 | 5.087157017 |
| 54 | AAEL026867-RC | 2.55561593 | 5.538656417 |
| 55 | AAEL020512-RA | 2.545301283 | 8.666312517 |
| 56 | AAEL007518-RL | 2.540172891 | 5.315615399 |
| 57 | AAEL010257-RA | 2.522332804 | 6.712596776 |
| 58 | AAEL007513-RA | 2.513525484 | 5.931339181 |
| 59 | AAEL000647-RA | 2.499265144 | 5.527333184 |
| 60 | AAEL007518-RJ | 2.452714759 | 5.401020363 |
| 61 | AAEL010659-RC | 2.437419873 | 5.223480073 |
| 62 | AAEL000667-RB | 2.425144336 | 5.95265545 |
| 63 | AAEL014450-RA | 2.408058294 | 5.779366752 |
| 64 | AAEL017500-RA | 2.403666278 | 5.726704476 |
| 65 | AAEL007288-RD | 2.398784217 | 5.34874326 |
| 66 | AAEL010659-RB | 2.375661317 | 7.186286715 |
| 67 | AAEL019525-RE | 2.371506791 | 5.206881862 |
| 68 | AAEL022261-RA | 2.368768336 | 6.747386709 |
| 69 | AAEL011890-RC | 2.363269959 | 6.549606116 |
| 70 | AAEL001863-RA | 2.361669969 | 6.857239581 |
| 71 | AAEL012062-RI | 2.360089254 | 5.261765252 |
| 72 | AAEL001082-RA | 2.353418087 | 9.498035678 |
| 73 | AAEL004597-RA | 2.344361157 | 5.744658641 |
| 74 | AAEL003430-RC | 2.320830467 | 5.174605992 |
| 75 | AAEL003419-RA | 2.319736318 | 5.134874456 |
| 76 | AAEL009670-RA | 2.312792785 | 7.164132591 |
| 77 | AAEL020097-RA | 2.309063894 | 5.315062796 |
| 78 | AAEL002606-RA | 2.297728935 | 7.802387338 |
| 79 | AAEL008485-RA | 2.273416953 | 7.577926169 |
| 80 | AAEL001031-RA | 2.234546913 | 5.382709483 |
| 81 | AAEL006526-RA | 2.233363569 | 5.779993737 |
| 82 | AAEL011981-RA | 2.211911617 | 6.291715584 |
| 83 | AAEL014419-RA | 2.210303989 | 6.979696431 |
| 84 | AAEL006719-RA | 2.200530357 | 5.009112789 |
| 85 | AAEL006854-RA | 2.199235813 | 5.426806901 |
| 86 | AAEL000288-RA | 2.196775255 | 5.31898296 |
| 87 | AAEL027052-RA | 2.193863269 | 8.0056667 |
| 88 | AAEL002764-RE | 2.181181828 | 5.297736647 |
| 89 | AAEL008289-RB | 2.170484988 | 9.216577867 |
| 90 | AAEL004798-RA | 2.155553088 | 7.270019464 |
| 91 | AAEL013458-RD | 2.144685092 | 7.079165583 |
| 92 | AAEL017563-RA | 2.118766201 | 10.8358652 |
| 93 | AAEL010884-RB | 2.103709712 | 10.52510766 |
| 94 | AAEL010205-RB | 2.102306963 | 6.310021197 |
| 95 | AAEL004816-RA | 2.094527649 | 5.344069447 |
| 96 | AAEL012596-RA | 2.091825397 | 8.781195831 |
| 97 | AAEL023527-RA | 2.079173615 | 5.971756941 |
| 98 | AAEL010260-RC | 2.079108925 | 6.975384465 |
| 99 | AAEL004522-RA | 2.076305782 | 7.060112577 |
| 100 | AAEL013515-RA | 2.06540154 | 8.422158232 |
| 101 | AAEL000165-RB | 2.05696973 | 5.082603131 |
| 102 | AAEL004212-RA | 2.048221816 | 5.019945827 |
| 103 | AAEL010783-RD | 2.044898313 | 6.41943 |
| 104 | AAEL006885-RC | 2.038657353 | 5.320208808 |
| 105 | AAEL026822-RA | 2.035923134 | 5.045088752 |
| 106 | AAEL003091-RA | 2.014673637 | 5.472812399 |
| 107 | AAEL005961-RA | 2.006089684 | 9.579405418 |
| 108 | AAEL001860-RA | 1.998180913 | 5.124677791 |
| 109 | AAEL020221-RA | 1.994648187 | 5.985422101 |
| 110 | AAEL017405-RA | 1.99179354 | 5.558483121 |
| 111 | AAEL009524-RA | 1.981626201 | 7.277291874 |
| 112 | AAEL010169-RA | 1.964921937 | 7.582181946 |
| 113 | AAEL003551-RB | 1.951592268 | 6.27930991 |
| 114 | AAEL017536-RD | 1.949677374 | 6.73822926 |
| 115 | AAEL007282-RB | 1.948259066 | 6.025673118 |
| 116 | AAEL008953-RG | 1.932838245 | 5.049357822 |
| 117 | AAEL022632-RA | 1.932174576 | 6.978544538 |
| 118 | AAEL024583-RK | 1.925230959 | 5.025854649 |
| 119 | AAEL016994-RA | 1.916769902 | 11.11352977 |
| 120 | AAEL027636-RA | 1.909191412 | 5.702798075 |
| 121 | AAEL009422-RE | 1.899290299 | 6.160607248 |
| 122 | AAEL013458-RF | 1.887024689 | 6.524684028 |
| 123 | AAEL006685-RB | 1.881460202 | 5.858931465 |
| 124 | AAEL007282-RC | 1.880134689 | 6.075628938 |
| 125 | AAEL017536-RC | 1.872687274 | 5.654233334 |
| 126 | AAEL024887-RA | 1.865606127 | 6.310535748 |
| 127 | AAEL000185-RD | 1.835186624 | 5.092169631 |
| 128 | AAEL021584-RA | 1.835173399 | 5.311165492 |
| 129 | AAEL009510-RA | 1.818744025 | 5.185123919 |
| 130 | AAEL008844-RA | 1.816245738 | 9.451418734 |
| 131 | AAEL012062-RD | 1.810124164 | 6.046946599 |
| 132 | AAEL003425-RA | 1.767202498 | 5.274152556 |
| 133 | AAEL001287-RA | 1.764474601 | 6.031320109 |
| 134 | AAEL001306-RB | 1.75266881 | 6.334054233 |
| 135 | AAEL001022-RA | 1.747674808 | 5.71630782 |
| 136 | AAEL002759-RG | 1.74122523 | 8.470006606 |
| 137 | AAEL001965-RA | 1.739365251 | 5.969686264 |
| 138 | AAEL002417-RC | 1.73300649 | 5.297659135 |
| 139 | AAEL004242-RB | 1.723275963 | 5.436442099 |
| 140 | AAEL014142-RA | 1.722482357 | 5.83005647 |
| 141 | AAEL003223-RA | 1.697661857 | 8.627862205 |
| 142 | AAEL007292-RB | 1.695421487 | 6.343826142 |
| 143 | AAEL004220-RA | 1.684188381 | 5.680059773 |
| 144 | AAEL006535-RA | 1.662611273 | 5.045032909 |
| 145 | AAEL011966-RA | 1.660866622 | 5.290376049 |
| 146 | AAEL026805-RA | 1.660866622 | 5.290376049 |
| 147 | AAEL025477-RA | 1.659606891 | 5.793207303 |
| 148 | AAEL016972-RC | 1.658814857 | 6.550957661 |
| 149 | AAEL009422-RD | 1.651860329 | 5.952021972 |
| 150 | AAEL010034-RA | 1.642622854 | 6.603587845 |
| 151 | AAEL000372-RA | 1.631845272 | 5.235553234 |
| 152 | AAEL019935-RB | 1.628316306 | 5.843824416 |
| 153 | AAEL021435-RA | 1.625791552 | 7.701547311 |
| 154 | AAEL010975-RB | 1.624965866 | 6.814053989 |
| 155 | AAEL001490-RA | 1.624178648 | 7.017700106 |
| 156 | AAEL005995-RA | 1.622628172 | 5.710708305 |
| 157 | AAEL012062-RK | 1.610416881 | 6.759651651 |
| 158 | AAEL004060-RC | 1.604135739 | 9.121787923 |
| 159 | AAEL011070-RA | 1.603982417 | 7.517524282 |
| 160 | AAEL021056-RA | 1.602986725 | 5.470025745 |
| 161 | AAEL002470-RA | 1.601384687 | 7.020031246 |
| 162 | AAEL012326-RA | 1.585556348 | 8.862330801 |
| 163 | AAEL003934-RA | 1.572788702 | 5.957921851 |
| 164 | AAEL010814-RC | 1.555543704 | 6.994677688 |
| 165 | AAEL010789-RA | 1.554244795 | 5.247227364 |
| 166 | AAEL001390-RA | 1.523616531 | 11.08116758 |
| 167 | AAEL005768-RA | 1.520040156 | 9.079350426 |
| 168 | AAEL023634-RA | 1.519168765 | 5.165094495 |
| 169 | AAEL001928-RA | 1.513819194 | 7.008656546 |
| 170 | AAEL017074-RA | 1.512369682 | 5.736994416 |
| 171 | AAEL019856-RA | 1.509998198 | 5.032192435 |
| 172 | AAEL008953-RD | 1.504329087 | 6.030028742 |
| 173 | AAEL014609-RA | 1.503677765 | 5.286115608 |
| 174 | AAEL007020-RA | 1.499976392 | 5.490773312 |
| 175 | AAEL003345-RA | 1.493559671 | 6.101216233 |
| 176 | AAEL012064-RB | 1.493548011 | 7.18288125 |
| 177 | AAEL011126-RB | 1.492303394 | 5.027376731 |
| 178 | AAEL004249-RB | 1.488185262 | 8.199566628 |
| 179 | AAEL007293-RD | 1.486816265 | 6.318781448 |
| 180 | AAEL027773-RA | 1.485447271 | 5.218790398 |
| 181 | AAEL024233-RA | 1.478731528 | 7.372479704 |
| 182 | AAEL001593-RD | 1.474382152 | 8.74836469 |
| 183 | AAEL004278-RA | 1.472004421 | 5.831306671 |
| 184 | AAEL000544-RA | 1.46746378 | 5.759764441 |
| 185 | AAEL017805-RA | 1.465746241 | 5.183063447 |
| 186 | AAEL025199-RB | 1.465273544 | 5.197537964 |
| 187 | AAEL014937-RA | 1.459284867 | 7.602652087 |
| 188 | AAEL001673-RA | 1.457321931 | 9.412950278 |
| 189 | AAEL017320-RA | 1.451983768 | 5.252676985 |
| 190 | AAEL002491-RA | 1.451963953 | 5.005539005 |
| 191 | AAEL006572-RC | 1.442384595 | 7.766615983 |
| 192 | AAEL010205-RD | 1.440526661 | 6.90120254 |
| 193 | AAEL011116-RA | 1.434079791 | 7.206061605 |
| 194 | AAEL026075-RA | 1.425867065 | 5.585002359 |
| 195 | AAEL004119-RC | 1.422515247 | 5.338798078 |
| 196 | AAEL026967-RA | 1.42179192 | 9.949359192 |
| 197 | AAEL009629-RA | 1.421534029 | 5.399215595 |
| 198 | AAEL010850-RM | 1.421195736 | 5.198220539 |
| 199 | AAEL002417-RE | 1.417809385 | 5.122779295 |
| 200 | AAEL002185-RA | 1.412336674 | 8.984476109 |
| 201 | AAEL000211-RA | 1.40169083 | 8.127590227 |
| 202 | AAEL015202-RB | 1.397026374 | 5.197498961 |
| 203 | AAEL011639-RA | 1.394571235 | 8.116715374 |
| 204 | AAEL001913-RA | 1.393196092 | 6.71019162 |
| 205 | AAEL009812-RA | 1.392107141 | 5.145117036 |
| 206 | AAEL028128-RA | 1.390242373 | 6.41236825 |
| 207 | AAEL002721-RA | 1.384735238 | 5.175052266 |
| 208 | AAEL011263-RA | 1.383956409 | 5.126314218 |
| 209 | AAEL017098-RB | 1.379514562 | 6.345882789 |
| 210 | AAEL013524-RA | 1.378225095 | 5.798627912 |
| 211 | AAEL011776-RA | 1.376558284 | 6.006237412 |
| 212 | AAEL013486-RA | 1.376376274 | 8.46373931 |
| 213 | AAEL019718-RA | 1.371060076 | 7.734528892 |
| 214 | AAEL022134-RA | 1.370552392 | 5.664758901 |
| 215 | AAEL013407-RB | 1.364246291 | 5.330444334 |
| 216 | AAEL019719-RA | 1.362900513 | 9.265256253 |
| 217 | AAEL028877-RA | 1.360396993 | 6.448426661 |
| 218 | AAEL006446-RA | 1.353012336 | 5.931357685 |
| 219 | AAEL002283-RA | 1.350221571 | 5.191921017 |
| 220 | AAEL002834-RC | 1.339106367 | 6.263510737 |
| 221 | AAEL009955-RB | 1.336639498 | 5.892140073 |
| 222 | AAEL002572-RA | 1.333617844 | 10.49424624 |
| 223 | AAEL020092-RA | 1.331126748 | 5.711263298 |
| 224 | AAEL003067-RA | 1.327576954 | 7.414978085 |
| 225 | AAEL017082-RA | 1.325901022 | 5.14812088 |
| 226 | AAEL008680-RB | 1.321839618 | 5.078801962 |
| 227 | AAEL004699-RA | 1.318748182 | 7.756774075 |
| 228 | AAEL006582-RH | 1.317173343 | 8.081435139 |
| 229 | AAEL009313-RA | 1.313973092 | 5.759938775 |
| 230 | AAEL028924-RA | 1.312757385 | 7.417759744 |
| 231 | AAEL005790-RA | 1.30866749 | 6.901178916 |
| 232 | AAEL014846-RA | 1.299298718 | 5.354004443 |
| 233 | AAEL021755-RA | 1.298967557 | 6.523496018 |
| 234 | AAEL010956-RA | 1.295816352 | 5.719645712 |
| 235 | AAEL005314-RA | 1.295747767 | 6.681599964 |
| 236 | AAEL013052-RA | 1.294755556 | 5.400268333 |
| 237 | AAEL013484-RA | 1.291734692 | 5.0290826 |
| 238 | AAEL021259-RA | 1.289832332 | 5.001628021 |
| 239 | AAEL014847-RA | 1.287594161 | 6.495519155 |
| 240 | AAEL020172-RA | 1.287467123 | 5.244533253 |
| 241 | AAEL017455-RB | 1.284228824 | 6.949698907 |
| 242 | AAEL028897-RA | 1.281074541 | 6.379528773 |
| 243 | AAEL006271-RD | 1.280667242 | 6.497906023 |
| 244 | AAEL009387-RF | 1.279792412 | 5.109571557 |
| 245 | AAEL006741-RA | 1.278860925 | 6.593327569 |
| 246 | AAEL005515-RF | 1.27272157 | 5.621995067 |
| 247 | AAEL022090-RA | 1.272358021 | 5.297891275 |
| 248 | AAEL000445-RC | 1.271464798 | 6.286860342 |
| 249 | AAEL006890-RB | 1.267761929 | 5.693992628 |
| 250 | AAEL006582-RE | 1.267204284 | 8.519703423 |
| 251 | AAEL017212-RA | 1.266825065 | 8.064852942 |
| 252 | AAEL018117-RB | 1.266399295 | 6.020999428 |
| 253 | AAEL001625-RA | 1.265251795 | 5.087032452 |
| 254 | AAEL006885-RB | 1.263547406 | 8.521698415 |
| 255 | AAEL025746-RA | 1.261824356 | 5.695609092 |
| 256 | AAEL001579-RA | 1.255754888 | 9.576639845 |
| 257 | AAEL011109-RD | 1.254717559 | 5.172918048 |
| 258 | AAEL009115-RA | 1.254332673 | 7.355367213 |
| 259 | AAEL002638-RA | 1.254065364 | 5.553782566 |
| 260 | AAEL007979-RB | 1.252965471 | 5.57713295 |
| 261 | AAEL002083-RA | 1.251705833 | 8.324816805 |
| 262 | AAEL001295-RA | 1.251239336 | 5.422278014 |
| 263 | AAEL015065-RA | 1.25058623 | 5.093035929 |
| 264 | AAEL012243-RA | 1.24786018 | 5.47661297 |
| 265 | AAEL009185-RE | 1.247770876 | 6.676475894 |
| 266 | AAEL011575-RA | 1.247712272 | 5.202941383 |
| 267 | AAEL017376-RA | 1.247653684 | 5.727300143 |
| 268 | AAEL009585-RB | 1.24221605 | 5.407978057 |
| 269 | AAEL004987-RA | 1.238988025 | 6.521663087 |
| 270 | AAEL018746-RA | 1.236115734 | 5.670849493 |
| 271 | AAEL024451-RA | 1.234868278 | 5.238023676 |
| 272 | AAEL017302-RA | 1.23104999 | 6.652181655 |
| 273 | AAEL026403-RA | 1.229658573 | 9.385870503 |
| 274 | AAEL019283-RA | 1.229242331 | 6.309737405 |
| 275 | AAEL028855-RA | 1.229242331 | 6.309737405 |
| 276 | AAEL028961-RA | 1.229242331 | 6.309737405 |
| 277 | AAEL006169-RA | 1.227334435 | 7.467034669 |
| 278 | AAEL023171-RA | 1.225117052 | 5.272806389 |
| 279 | AAEL003104-RB | 1.22221782 | 5.222635475 |
| 280 | AAEL027218-RA | 1.220125879 | 5.759909344 |
| 281 | AAEL002761-RAI | 1.219095055 | 8.91324097 |
| 282 | AAEL008006-RA | 1.217510637 | 6.028894702 |
| 283 | AAEL010148-RA | 1.216980029 | 5.866323898 |
| 284 | AAEL020990-RA | 1.21691813 | 7.74186857 |
| 285 | AAEL017301-RC | 1.214717043 | 6.722499521 |
| 286 | AAEL012964-RD | 1.214073233 | 6.428865948 |
| 287 | AAEL006377-RA | 1.211741531 | 5.033819752 |
| 288 | AAEL012113-RA | 1.209156506 | 5.309726856 |
| 289 | AAEL008789-RA | 1.207513987 | 7.910751367 |
| 290 | AAEL010470-RA | 1.205358225 | 5.878863663 |
| 291 | AAEL003312-RA | 1.203704976 | 5.115262283 |
| 292 | AAEL001964-RA | 1.201374101 | 6.050624517 |
| 293 | AAEL027216-RA | 1.19858749 | 6.312396197 |
| 294 | AAEL011221-RB | 1.194613079 | 5.592408628 |
| 295 | AAEL000621-RA | 1.190117799 | 7.286613802 |
| 296 | AAEL017540-RA | 1.189543402 | 5.223405779 |
| 297 | AAEL006103-RC | 1.187772711 | 5.302502281 |
| 298 | AAEL013458-RE | 1.185622891 | 7.314395104 |
| 299 | AAEL004657-RA | 1.184942679 | 6.20031415 |
| 300 | AAEL005951-RD | 1.184008911 | 5.449100139 |
| 301 | AAEL025995-RB | 1.183972045 | 5.516242538 |
| 302 | AAEL020382-RA | 1.178333237 | 5.220827915 |
| 303 | AAEL009181-RA | 1.177812679 | 7.834023934 |
| 304 | AAEL000670-RA | 1.173351216 | 5.817837657 |
| 305 | AAEL000124-RA | 1.170612273 | 5.855581249 |
| 306 | AAEL008131-RA | 1.166608437 | 5.791524494 |
| 307 | AAEL009274-RA | 1.16604254 | 5.743557666 |
| 308 | AAEL018102-RB | 1.16584149 | 7.858619894 |
| 309 | AAEL017144-RA | 1.162758699 | 7.089642695 |
| 310 | AAEL005032-RC | 1.162117671 | 6.080838938 |
| 311 | AAEL011789-RC | 1.161995562 | 8.579967396 |
| 312 | AAEL012311-RC | 1.161609859 | 5.002262307 |
| 313 | AAEL012017-RA | 1.159978723 | 6.990387891 |
| 314 | AAEL007042-RE | 1.15797222 | 5.678092869 |
| 315 | AAEL004436-RA | 1.156492242 | 5.295070761 |
| 316 | AAEL021666-RD | 1.156127821 | 5.130235826 |
| 317 | AAEL024403-RA | 1.15608384 | 5.401307035 |
| 318 | AAEL012114-RA | 1.154698038 | 6.684863951 |
| 319 | AAEL025597-RE | 1.151426952 | 5.339252241 |
| 320 | AAEL017116-RB | 1.149746266 | 5.797932331 |
| 321 | AAEL000886-RA | 1.14928496 | 6.388218851 |
| 322 | AAEL002817-RB | 1.14708266 | 5.672537771 |
| 323 | AAEL011830-RF | 1.147081412 | 5.501019814 |
| 324 | AAEL014768-RB | 1.145751854 | 5.743867022 |
| 325 | AAEL027335-RA | 1.143507584 | 5.082266034 |
| 326 | AAEL001176-RB | 1.143251905 | 5.191387705 |
| 327 | AAEL005515-RM | 1.142834624 | 6.32458645 |
| 328 | AAEL024387-RA | 1.142344027 | 6.808165532 |
| 329 | AAEL013229-RA | 1.140725269 | 7.85190912 |
| 330 | AAEL019523-RB | 1.136998279 | 5.143262008 |
| 331 | AAEL021929-RA | 1.136702082 | 5.157591029 |
| 332 | AAEL027762-RA | 1.135760105 | 6.452191625 |
| 333 | AAEL025597-RA | 1.134252677 | 6.692338453 |
| 334 | AAEL002759-RE | 1.132648389 | 6.878162785 |
| 335 | AAEL006389-RC | 1.131042674 | 5.334338726 |
| 336 | AAEL014246-RC | 1.130777651 | 6.401251433 |
| 337 | AAEL007298-RD | 1.13009162 | 5.679999984 |
| 338 | AAEL008727-RA | 1.127223864 | 9.364194638 |
| 339 | AAEL011650-RA | 1.126879633 | 5.333159125 |
| 340 | AAEL007889-RC | 1.124556841 | 5.360093193 |
| 341 | AAEL007542-RA | 1.123665489 | 5.688983498 |
| 342 | AAEL003006-RB | 1.123607516 | 5.942265902 |
| 343 | AAEL000160-RA | 1.121201095 | 10.554257 |
| 344 | AAEL023854-RA | 1.118056996 | 7.119098106 |
| 345 | AAEL007349-RB | 1.117690994 | 5.567347735 |
| 346 | AAEL009695-RB | 1.11494875 | 5.47280794 |
| 347 | AAEL023743-RA | 1.111054887 | 7.856110518 |
| 348 | AAEL000657-RA | 1.110385096 | 5.652747182 |
| 349 | AAEL003593-RA | 1.108876751 | 5.319711769 |
| 350 | AAEL003459-RA | 1.108567896 | 6.361894713 |
| 351 | AAEL000328-RA | 1.107457846 | 5.658113201 |
| 352 | AAEL003203-RD | 1.105623851 | 6.852227867 |
| 353 | AAEL017247-RA | 1.100700749 | 11.90554064 |
| 354 | AAEL013612-RF | 1.095678659 | 5.244600734 |
| 355 | AAEL027802-RA | 1.093600736 | 7.696888969 |
| 356 | AAEL008397-RA | 1.09316906 | 6.133222164 |
| 357 | AAEL024916-RA | 1.091737163 | 9.042081287 |
| 358 | AAEL001323-RA | 1.091326751 | 6.356251525 |
| 359 | AAEL014600-RA | 1.087238923 | 6.022205855 |
| 360 | AAEL004141-RE | 1.086705667 | 5.41298867 |
| 361 | AAEL004496-RE | 1.086549972 | 6.334755513 |
| 362 | AAEL019798-RA | 1.085087669 | 7.033679156 |
| 363 | AAEL023573-RA | 1.084882867 | 5.022452958 |
| 364 | AAEL017481-RA | 1.08167128 | 7.607616952 |
| 365 | AAEL001128-RA | 1.081282554 | 5.206668286 |
| 366 | AAEL005336-RA | 1.079690991 | 5.882444593 |
| 367 | AAEL009337-RB | 1.074237688 | 7.371556283 |
| 368 | AAEL019379-RA | 1.0739036 | 5.391213617 |
| 369 | AAEL028845-RA | 1.0739036 | 5.391213617 |
| 370 | AAEL020106-RA | 1.073633938 | 5.133434208 |
| 371 | AAEL001511-RA | 1.072868247 | 5.558671698 |
| 372 | AAEL017301-RA | 1.071049078 | 7.274303921 |
| 373 | AAEL027613-RA | 1.065012604 | 6.284604282 |
| 374 | AAEL007707-RA | 1.064277733 | 6.003051894 |
| 375 | AAEL013431-RU | 1.062891334 | 5.357502274 |
| 376 | AAEL012062-RE | 1.058889442 | 7.664809157 |
| 377 | AAEL023854-RB | 1.058130407 | 7.121524547 |
| 378 | AAEL014185-RA | 1.056175605 | 6.191158033 |
| 379 | AAEL006516-RA | 1.052547831 | 5.109472713 |
| 380 | AAEL011320-RA | 1.050660649 | 6.769685029 |
| 381 | AAEL010145-RE | 1.049771289 | 7.439814008 |
| 382 | AAEL003128-RA | 1.045182222 | 5.456554041 |
| 383 | AAEL010004-RB | 1.043106811 | 5.698246225 |
| 384 | AAEL008723-RC | 1.042565511 | 5.768192958 |
| 385 | AAEL010538-RA | 1.042536343 | 5.096904796 |
| 386 | AAEL005845-RB | 1.041819729 | 6.015072727 |
| 387 | AAEL007548-RA | 1.041649281 | 5.957673484 |
| 388 | AAEL019526-RA | 1.041069349 | 5.176589601 |
| 389 | AAEL022980-RA | 1.038874661 | 5.401933316 |
| 390 | AAEL001351-RA | 1.038277334 | 5.540541669 |
| 391 | AAEL006179-RB | 1.038212406 | 5.50824799 |
| 392 | AAEL019797-RA | 1.037932025 | 6.531115395 |
| 393 | AAEL001094-RA | 1.03774833 | 5.858799057 |
| 394 | AAEL001194-RA | 1.03737475 | 6.557602996 |
| 395 | AAEL013279-RB | 1.035973123 | 7.929145318 |
| 396 | AAEL014913-RB | 1.033578298 | 6.540584357 |
| 397 | AAEL027549-RA | 1.03345272 | 6.915112648 |
| 398 | AAEL014484-RA | 1.031020292 | 6.106775466 |
| 399 | AAEL011890-RB | 1.030028209 | 7.389209753 |
| 400 | AAEL022603-RA | 1.028966897 | 5.804224431 |
| 401 | AAEL023869-RA | 1.028966897 | 5.804224431 |
| 402 | AAEL009637-RA | 1.028841385 | 6.860677871 |
| 403 | AAEL024545-RA | 1.028660402 | 5.660986868 |
| 404 | AAEL011873-RA | 1.028178996 | 5.100423627 |
| 405 | AAEL009275-RA | 1.025088197 | 5.866478164 |
| 406 | AAEL004457-RE | 1.024419746 | 6.471112063 |
| 407 | AAEL019822-RA | 1.02185494 | 6.403375605 |
| 408 | AAEL008073-RA | 1.021848133 | 7.40745223 |
| 409 | AAEL007659-RA | 1.020247899 | 5.310516961 |
| 410 | AAEL017085-RA | 1.020245017 | 5.133608488 |
| 411 | AAEL008651-RA | 1.019160064 | 5.773769585 |
| 412 | AAEL010975-RA | 1.018844961 | 7.651238476 |
| 413 | AAEL023684-RA | 1.018770651 | 5.351074517 |
| 414 | AAEL015306-RA | 1.016652244 | 7.783559746 |
| 415 | AAEL004457-RD | 1.015016448 | 10.06420948 |
| 416 | AAEL017406-RA | 1.014953485 | 5.88760895 |
| 417 | AAEL013133-RA | 1.014943643 | 6.816155692 |
| 418 | AAEL016984-RA | 1.014436616 | 10.48748929 |
| 419 | AAEL001964-RB | 1.014162378 | 9.887697366 |
| 420 | AAEL026335-RA | 1.013457235 | 6.422705795 |
| 421 | AAEL002917-RA | 1.013091513 | 5.482693591 |
| 422 | AAEL012359-RA | 1.012435188 | 8.629572618 |
| 423 | AAEL012588-RA | 1.010981998 | 5.722325794 |
| 424 | AAEL005617-RB | 1.010575452 | 6.671074999 |
| 425 | AAEL006968-RA | 1.009530124 | 5.243103839 |
| 426 | AAEL008106-RA | 1.007396482 | 5.759054258 |
| 427 | AAEL007944-RA | 1.00381796 | 5.36453483 |
| 428 | AAEL012207-RF | 1.00308015 | 11.3188282 |
| 429 | AAEL006745-RA | 1.001815432 | 6.24691141 |
| 430 | AAEL012114-RB | 1.00141111 | 6.701334221 |

## Table S5: Raw data used to create Figure 5.

| **N1** | | | | | |  |  | **N2** | | | | |  |  | **N3** | | | | |
| --- | --- | --- | --- | --- | --- | --- | --- | --- | --- | --- | --- | --- | --- | --- | --- | --- | --- | --- | --- |
| **eiF1A** |  | **a** |  | **b** |  |  | **eiF1A** |  | **a** |  | **b** |  | **eiF1A** |  | **a** |  | **b** |  |  |
|  | **head** | F2h | 25.84537125 | F2h | 25.55444336 |  |  | **head** | F2h | 24.09986496 | F2h | 25.47465134 |  | **head** | F2h | 25.05502892 | F2h | 24.72233772 |  |
|  |  | F24h | 26.28053856 | F24h | 25.72532082 |  |  |  | F24h | 25.06018829 | F24h | 27.07487297 |  |  | F24h | 25.94018745 | F24h | 28.5826664 |  |
|  |  | F96h | 26.76944542 | F96h | 27.88435555 |  |  |  | F96h | 24.90779877 | F96h | 29.94364166 |  |  | F96h | 27.00431252 | F96h | 27.56942177 |  |
|  |  | M2h | 25.58454514 | M2h | 24.68947983 |  |  |  | M2h | 24.79315567 | M2h | 24.30462265 |  |  | M2h | 25.78943062 | M2h | 24.96296692 |  |
|  |  | M24h | 25.59124565 | M24h | 26.26392937 |  |  |  | M24h | 25.51702881 | M24h | 26.46090698 |  |  | M24h | 25.4083252 | M24h | 25.57702827 |  |
|  |  | M96h | 26.45151901 | M96h | 25.90134811 |  |  |  | M96h | 25.04011536 | M96h | 28.40176582 |  |  | M96h | 26.78837776 | M96h | 27.90398407 |  |
|  | **body** | F2h | 23.96679878 | F2h | 23.79315567 |  |  | **body** | F2h | 22.44442177 | F2h | 22.19914246 |  | **body** | F2h | 23.35492134 | F24h | 23.07408142 |  |
|  |  | F24h | 23.32240105 | F24h | 23.48915482 |  |  |  | F24h | 21.9730854 | F24h | 22.49297523 |  |  | F24h | 22.92860794 | F24h | 23.13096619 |  |
|  |  | F96h | 23.89072227 | F96h | 23.88030815 |  |  |  | F96h | 22.01382828 | F96h | 22.16500282 |  |  | F96h | 23.33530617 | F96h | 23.27595711 |  |
|  |  | M2h | 23.80131531 | M2h | 23.94511604 |  |  |  | M2h | 22.35322571 | M2h | 22.76481628 |  |  | M2h | 23.21374702 | M2h | 23.35806465 |  |
|  |  | M24h | 24.62648392 | M24h | 24.82411385 |  |  |  | M24h | 23.33322716 | M24h | 23.44300079 |  |  | M24h | 23.85375977 | M24h | 23.76151848 |  |
|  |  | M96h | 25.00161552 | M96h | Undetermined |  |  |  | M96h | 23.65142822 | M96h | 22.96022606 |  |  | M96h | 31.40329361 | M96h | 24.39575195 |  |
|  |  |  |  |  |  |  |  |  |  |  |  |  |  |  |  |  |  |  |  |
| **prdx5** |  | **a** |  | **b** |  |  | **prdx5** |  | **a** |  | **b** |  | **prdx5** |  | **a** |  | **b** |  |  |
|  | **head** | F2h | 26.65979004 | F2h | 27.01625443 |  |  | **head** | F2h | 26.28234482 | F2h | 27.09188843 |  | **head** | F2h | 27.55448723 | F2h | 25.95856094 |  |
|  |  | F24h | 28.04269791 | F24h | 27.66957855 |  |  |  | F24h | 26.48182678 | F24h | 27.55525589 |  |  | F24h | 27.17442703 | F24h | 29.27039719 |  |
|  |  | F96h | 28.60667992 | F96h | 30.94756126 |  |  |  | F96h | 25.75688934 | F96h | 27.86184502 |  |  | F96h | 28.99486542 | F96h | 31.72049332 |  |
|  |  | M2h | 26.02136421 | M2h | 25.64145851 |  |  |  | M2h | 26.0893898 | M2h | 24.55344772 |  |  | M2h | 26.8030014 | M2h | 26.33722496 |  |
|  |  | M24h | 26.60195732 | M24h | 28.6831131 |  |  |  | M24h | 27.93440056 | M24h | 27.49660301 |  |  | M24h | 27.87367058 | M24h | 27.55919266 |  |
|  |  | M96h | 30.37713051 | M96h | 29.28266716 |  |  |  | M96h | 26.39273834 | M96h | 27.7188282 |  |  | M96h | 30.34805489 | M96h | 31.9045105 |  |
|  | **body** | F2h | 24.46544456 | F2h | 24.3102684 |  |  | **body** | F2h | 21.73172569 | F2h | 21.62747574 |  | **body** | F2h | 23.50406456 | F24h | 23.57767296 |  |
|  |  | F24h | 23.13768005 | F24h | 23.51276588 |  |  |  | F24h | 21.27748299 | F24h | 21.41714859 |  |  | F24h | 22.56379509 | F24h | 22.83389282 |  |
|  |  | F96h | 24.69782066 | F96h | 25.10948372 |  |  |  | F96h | 22.75979614 | F96h | 24.5014801 |  |  | F96h | 24.27447701 | F96h | 23.86523056 |  |
|  |  | M2h | 24.00495529 | M2h | 24.11512756 |  |  |  | M2h | 21.92221451 | M2h | 22.54768372 |  |  | M2h | 23.67988396 | M2h | 22.95279503 |  |
|  |  | M24h | 24.86898041 | M24h | 25.25930405 |  |  |  | M24h | 23.21815109 | M24h | 24.38343048 |  |  | M24h | 23.93016052 | M24h | 24.11486816 |  |
|  |  | M96h | 26.74681664 | M96h | Undetermined |  |  |  | M96h | 24.26332855 | M96h | 24.20907021 |  |  | M96h | 32.59479904 | M96h | 27.0463295 |  |
|  |  |  |  |  |  |  |  |  |  |  |  |  |  |  |  |  |  |  |  |
| **eiF3j** |  | **a** |  | **b** |  |  | **eiF3j** |  | **a** |  | **b** |  | **eiF3j** |  | **a** |  | **b** |  |  |
|  | **head** | F2h | 26.7475872 | F2h | 26.81276703 |  |  | **head** | F2h | 24.28580284 | F2h | 25.73266983 |  | **head** | F2h | 25.69832802 | F2h | 26.00950623 |  |
|  |  | F24h | 27.5956707 | F24h | 26.70194817 |  |  |  | F24h | 25.65973854 | F24h | 26.92482567 |  |  | F24h | 27.43489075 | F24h | 29.34107208 |  |
|  |  | F96h | 28.6915493 | F96h | 29.68865204 |  |  |  | F96h | 26.34594727 | F96h | 28.68778229 |  |  | F96h | 28.30866814 | F96h | 28.61343956 |  |
|  |  | M2h | 26.40019989 | M2h | 26.36935806 |  |  |  | M2h | 25.2136631 | M2h | 25.26118088 |  |  | M2h | 26.62806129 | M2h | 25.76511383 |  |
|  |  | M24h | 27.04241371 | M24h | 27.93868828 |  |  |  | M24h | 26.00738144 | M24h | 26.91732025 |  |  | M24h | 26.96067429 | M24h | 26.97584534 |  |
|  |  | M96h | 28.09982109 | M96h | 27.75227737 |  |  |  | M96h | 26.33383179 | M96h | 27.92370033 |  |  | M96h | 27.8652668 | M96h | Undetermined |  |
|  | **body** | F2h | 25.05958366 | F2h | 24.89890671 |  |  | **body** | F2h | 22.6645298 | F2h | 22.80192947 |  | **body** | F2h | 24.40888786 | F24h | 23.9673214 |  |
|  |  | F24h | 24.2682457 | F24h | 24.42020035 |  |  |  | F24h | 22.67585754 | F24h | 22.52425766 |  |  | F24h | 24.25104523 | F24h | 24.1274929 |  |
|  |  | F96h | 24.71103477 | F96h | 25.07687378 |  |  |  | F96h | 22.95312119 | F96h | 22.83158493 |  |  | F96h | 24.37534904 | F96h | 24.49131203 |  |
|  |  | M2h | 25.22685814 | M2h | 25.18385506 |  |  |  | M2h | 23.16255951 | M2h | 23.50641441 |  |  | M2h | 24.68647194 | M2h | 24.32630157 |  |
|  |  | M24h | 25.53769112 | M24h | 25.99933624 |  |  |  | M24h | 24.25101662 | M24h | 24.00727463 |  |  | M24h | 24.50030136 | M24h | 24.76396179 |  |
|  |  | M96h | 26.41874695 | M96h | 30.20453072 |  |  |  | M96h | 24.70326424 | M96h | 24.23211098 |  |  | M96h | 29.92533493 | M96h | 25.900877 |  |
|  |  |  |  |  |  |  |  |  |  |  |  |  |  |  |  |  |  |  |  |
| **RpL32** |  | **a** |  | **b** |  |  | **RpL32** |  | **a** |  | **b** |  |  |  |  | **a** |  | **b** |  |
|  | **head** | F2h | 23.63260269 | F2h | 24.74130821 |  |  | **head** | F2h | 24.7565937 | F2hN3 | 26.71870232 | **RpL32** |  | F2h | 23.68310928 | F2h | 23.14880562 |  |
|  |  | F24h | 24.97300339 | F24h | 24.49243736 |  |  |  | F24h | 25.61169052 | F24hN3 | 29.01123047 |  | **head** | F24h | 24.74608421 | F24h | 26.9407711 |  |
|  |  | F96h | 25.55077171 | F96h | 27.80699921 |  |  |  | F96h | 24.80362701 | F96hN3 | 32.93848801 |  |  | F96h | 26.00810051 | F96h | 28.16075134 |  |
|  |  | M2h | 23.47716904 | M2h | 23.35330009 |  |  |  | M2h | 25.05202675 | M2hN3 | 24.06661034 |  |  | M2h | 23.45853233 | M2h | 23.75007629 |  |
|  |  | M24h | 24.07065773 | M24h | 25.40481567 |  |  |  | M24h | 25.61279297 | M24hN3 | 27.50928879 |  |  | M24h | 25.04042625 | M24h | 24.90020561 |  |
|  |  | M96h | 26.13851547 | M96h | 25.91725159 |  |  |  | M96h | 25.97231674 | M96hN3 | 29.71906471 |  |  | M96h | 26.96600533 | M96h | 28.52402878 |  |
|  | **body** | F2h | 21.64985085 | F2h | 22.01837158 |  |  | **body** | F2h | 21.98394394 | F2hN3 | 21.59083366 |  |  | F2h | 21.29936409 | F2h | 21.43948936 |  |
|  |  | F24h | 20.48493004 | F24h | 20.93242073 |  |  |  | F24h | 20.93998146 | F24hN3 | 21.27971077 |  | **body** | F24h | 20.56960487 | F24h | 20.22148132 |  |
|  |  | F96h | 20.46960831 | F96h | 20.82177734 |  |  |  | F96h | 20.47786713 | M96hN3 | 21.6939373 |  |  | F96h | 20.17118263 | F96h | 20.65032959 |  |
|  |  | M2h | 21.92571449 | M2h | 22.10983658 |  |  |  | M2h | 21.88234138 | M2hN3 | 22.48529816 |  |  | M2h | 21.62062454 | M2h | 21.52266502 |  |
|  |  | M24h | 23.1264286 | M24h | 23.50624466 |  |  |  | M24h | 23.05187988 | M24hN3 | 23.97144318 |  |  | M24h | 22.54045105 | M24h | 23.07479095 |  |
|  |  | M96h | 23.19864655 | M96h | 34.55976105 |  |  |  | M96h | 23.34485245 | M96hN3 | 22.99543571 |  |  | M96h | 36.98610306 | M96h | 23.62598801 |  |
|  |  |  |  |  |  |  |  |  |  |  |  |  |  |  |  |  |  |  |  |
| **Act** |  | **a** |  | **b** |  |  | **Act** |  | **a** |  | **b** |  |  |  |  | **a** |  | **b** |  |
|  | **head** | F2h | 22.79966736 | F2h | 23.20086479 |  |  | **head** | F2h | 22.72968102 | F2h | 24.76996803 | **Act** |  | F2h | 22.09473419 | F2h | 22.51663971 |  |
|  |  | F24h | 24.61504364 | F24h | 23.83221054 |  |  |  | F24h | 24.23806572 | F24h | 26.93396187 |  | **head** | F24h | 24.46253586 | F24h | 27.94836998 |  |
|  |  | F96h | 25.40070343 | F96h | 28.10200691 |  |  |  | F96h | 24.96289825 | F96h | 30.13910484 |  |  | F96h | 25.54178429 | F96h | 27.22899055 |  |
|  |  | M2h | 21.85173988 | M2h | 22.06139183 |  |  |  | M2h | 22.81450081 | M2h | 22.79679489 |  |  | M2h | 22.89771271 | M2h | 22.92392731 |  |
|  |  | M24h | 23.07306099 | M24h | 24.95771027 |  |  |  | M24h | 24.02403831 | M24h | 25.63018036 |  |  | M24h | 23.92162323 | M24h | 24.17559433 |  |
|  |  | M96h | 25.71889305 | M96h | 24.52859116 |  |  |  | M96h | 24.42122269 | M96h | 28.11298752 |  |  | M96h | 25.21521568 | M96h | 27.37851334 |  |
|  | **body** | F2h | 22.26718521 | F2h | 22.35219574 |  |  | **body** | F2h | 21.8345871 | F2h | 21.42153168 |  |  | F2h | 21.67510223 | F2h | 22.07351494 |  |
|  |  | F24h | 20.98044014 | F24h | 20.96942329 |  |  |  | F24h | 20.99277496 | F24h | 20.96547508 |  | **body** | F24h | 20.98032379 | F24h | 21.12722206 |  |
|  |  | F96h | 21.18025398 | F96h | 21.8260994 |  |  |  | F96h | 21.7758007 | F96h | Undetermined |  |  | F96h | 20.97020531 | F96h | 21.76252556 |  |
|  |  | M2h | 22.02942085 | M2h | 22.54564095 |  |  |  | M2h | 21.8137188 | M2h | 22.61876488 |  |  | M2h | 22.06007004 | M2h | 21.98163605 |  |
|  |  | M24h | 22.8631115 | M24h | Undetermined |  |  |  | M24h | 22.88730049 | M24h | 22.98814201 |  |  | M24h | 22.33451271 | M24h | 22.48871994 |  |
|  |  | M96h | 23.9224472 | M96h | 32.57335663 |  |  |  | M96h | 22.2700634 | M96h | 22.6216526 |  |  | M96h | 31.9345932 | M96h | 23.69161797 |  |
|  |  |  |  |  |  |  |  |  |  |  |  |  |  |  |  |  |  |  |  |
| **RPS7** |  | **a** |  | **b** |  |  | **RPS7** |  | **a** |  | **b** |  |  |  | **a** |  | **b** |  |  |
|  | **head** | F2h | 23.1388588 | F2h | 23.81256104 |  |  | **head** | F2h | 22.53582764 | F2h | 24.19582939 | **RPS7** |  | F2h | 20.31117821 | F2h | 20.23985767 |  |
|  |  | F24h | 24.41305161 | F24h | 23.86049557 |  |  |  | F24h | 23.86678696 | F24h | 25.94269562 |  | **head** | F24h | 21.3335228 | F24h | 23.87521362 |  |
|  |  | F96h | 24.70220852 | F96h | 26.28577137 |  |  |  | F96h | 20.9678688 | F96h | 27.99699783 |  |  | F96h | 21.79290295 | F96h | 22.81171799 |  |
|  |  | M2h | 23.33498669 | M2h | 22.96683407 |  |  |  | M2h | 23.33979702 | M2h | 23.11161995 |  |  | M2h | 20.76778126 | M2h | 20.27871132 |  |
|  |  | M24h | 24.06235981 | M24h | 24.50963783 |  |  |  | M24h | 24.16473675 | M24h | 24.78301334 |  |  | M24h | 20.98138428 | M24h | 22.17102528 |  |
|  |  | M96h | 24.46619225 | M96h | 24.11326981 |  |  |  | M96h | 21.94953918 | M96h | 26.78060722 |  |  | M96h | 21.85424805 | M96h | 23.58815765 |  |
|  | **body** | F2h | 21.53424263 | F2h | 21.48461151 |  |  | **body** | F2h | 21.08175087 | F2h | 20.88294601 |  |  | F2h | 20.66666889 | F2h | 20.89129066 |  |
|  |  | F24h | 20.53259182 | F24h | 20.25324249 |  |  |  | F24h | 20.59783268 | F24h | 20.19457436 |  | **body** | F24h | 20.44834328 | F24h | 20.25875378 |  |
|  |  | F96h | 19.75457764 | F96h | 20.25727272 |  |  |  | F96h | 19.49720573 | F96h | 19.63666248 |  |  | F96h | 19.37007523 | F96h | 19.77013969 |  |
|  |  | M2h | 21.47441864 | M2h | 21.55540276 |  |  |  | M2h | 21.24445724 | M2h | 21.49298 |  |  | M2h | 21.02060509 | M2h | 20.89272785 |  |
|  |  | M24h | 22.16353798 | M24h | 22.42657852 |  |  |  | M24h | 22.24968529 | M24h | 22.33788395 |  |  | M24h | 21.46570873 | M24h | 21.59983921 |  |
|  |  | M96h | 21.93311501 | M96h | 29.94459248 |  |  |  | M96h | 22.50457954 | M96h | 30.7478075 |  |  | M96h | 30.17815018 | M96h | 22.18284607 |  |
|  |  |  |  |  |  |  |  |  |  |  |  |  |  |  |  |  |  |  |  |
| **MSP** |  | **a** |  | **b** |  |  | **MSP** |  | **a** |  | **b** |  |  |  | **a** |  | **b** |  |  |
|  | **head** | F2h | 27.22077942 | F2h | 27.60442162 |  |  | **head** | F2h | 29.69477654 | F2h | 31.55027199 | **MSP** |  | F2h | 29.46741676 | F2h | 27.28535843 |  |
|  |  | F24h | 28.82545662 | F24h | 28.09383392 |  |  |  | F24h | 30.16440392 | F24h | 32.19486618 |  | **head** | F24h | 28.88094139 | F24h | 32.34899902 |  |
|  |  | F96h | 28.87694931 | F96h | 31.47694397 |  |  |  | F96h | 29.93873405 | F96h | 37.09329605 |  |  | F96h | 29.82739067 | F96h | 31.67168045 |  |
|  |  | M2h | 26.33742714 | M2h | 26.24094582 |  |  |  | M2h | 29.65094566 | M2h | 28.54637718 |  |  | M2h | 27.6586132 | M2h | 27.77591324 |  |
|  |  | M24h | 27.22969246 | M24h | 29.81625175 |  |  |  | M24h | 29.77364349 | M24h | 30.72628021 |  |  | M24h | 29.38642502 | M24h | 28.88563919 |  |
|  |  | M96h | 30.88416672 | M96h | 28.76035881 |  |  |  | M96h | 29.58639908 | M96h | 34.46308517 |  |  | M96h | 32.25529099 | M96h | 31.54019547 |  |
|  | **body** | F2h | 24.96698952 | F2h | 24.97270012 |  |  | **body** | F2h | 24.99920082 | F2h | 25.00349998 |  |  | F2h | 25.11685181 | F2h | 24.8157711 |  |
|  |  | F24h | 24.13500786 | F24h | 24.90129852 |  |  |  | F24h | 24.8081398 | F24h | 24.80350685 |  | **body** | F24h | 24.34242439 | F24h | 24.33578682 |  |
|  |  | F96h | 24.66639328 | F96h | 25.75976563 |  |  |  | F96h | 27.12086105 | F96h | 28.37555695 |  |  | F96h | 24.7046051 | F96h | 25.12689018 |  |
|  |  | M2h | 24.4931221 | M2h | 25.22363091 |  |  |  | M2h | 24.91340256 | M2h | 25.65713501 |  |  | M2h | 25.54634285 | M2h | 24.69351387 |  |
|  |  | M24h | 25.9505291 | M24h | 26.01790428 |  |  |  | M24h | 27.1293602 | M24h | 28.33381653 |  |  | M24h | 25.45851898 | M24h | 25.92885208 |  |
|  |  | M96h | 27.10085106 | M96h | 34.63465118 |  |  |  | M96h | 26.56156731 | M96h | 26.39205551 |  |  | M96h | 29.77729034 | M96h | 28.29150009 |  |
|  |  |  |  |  |  |  |  |  |  |  |  |  |  |  |  |  |  |  |  |

## Table S6: List of primers and their characteristics. GSV ID: GSV rank order. ID: VectorBase code.

| **GSV ID** | **Gene** | **ID** | **Primers** | **amplicon (bp)** | **Cq Range** | **R2** | **E%** |
| --- | --- | --- | --- | --- | --- | --- | --- |
| 2 | mitochondrial splicing protein (*MSP*) | AAEL006564-RB | Fw 5´ TCCAGAAGCTGCTCAACAAA | 98 | 17.6 - 27.9 | 0.99 | 91.43 |
|  |  |  | Rv 5´ GGGTGTAGTGAGGGCTGAAG |  |  |  |  |
| 3 | eukariotic translation factor 1A (*eiF1A*) | AAEL004378-RA | Fw 5´ GGTGTCAAGCGATTGTGTCA | 83 | 19.9 - 29.1 | 0.99 | 109.8 |
|  |  |  | Rv 5´ AAGCCGATGAGGATGATGTC |  |  |  |  |
| 5 | peroxiredoxin 5 (*prdx5*) | AAEL007135-RA | Fw 5´ CCAGATTAAGGAAGGCGACA | 111 | 22.3 - 32.7 | 0.99 | 94.12 |
|  |  |  | Rv 5´ GCGAACAGGACCACTTTCTT |  |  |  |  |
| 6 | eukariotic translation factor 3, subunit J (*eiF3j*) | AAEL012279-RA | Fw 5 GGACGAGCTTGAGGAGAAAA | 120 | 20.9 - 30.5 | 0.99 | 101.46 |
|  |  |  | Rv 5 CGCTCAGCTTCTTCTTGCTT |  |  |  |  |
| 13 | Ribosomal Protein S7 (*RpS7*) | AAEL009496-RA | Fw 5´ ACCGCCGTCTACGATGCCA | 112 | 16.2 - 25.9 | 0.99 | 102.78 |
|  |  |  | Rv 5´ ATGGTGGTCTGCTGGTTCTT |  |  |  |  |
| 153 | Ribosomal protein L32 (*RpL32*) | AAEL003396-RA | Fw 5´ GCTATGACAAGCTTGCCCCCA | 189 | 15.9 - 25.9 | 0.99 | 99.77 |
|  |  |  | Rv 5´ TCATCAGCACCTCCAGCTC |  |  |  |  |
| 661 | Actin (*ACT*) | AAEL011197-RC | Fw 5´ CGTTCGTGACATCAAGGAAA | 175 | 17.6 - 27.9 | 0.99 | 95.19 |
| 692 |  | AAEL011197-RD | Rv 5´ GAACGATGGCTGGAAGAGAG |  |  |  |  |

## Supplementary methodology: RNA extraction and quantitative PCR (qPCR)

*Aedes aegypti* samples were collected and processed as previously described [(37)](https://paperpile.com/c/xFERzV/l0l4). Briefly, Aedes Rio (F9-10 generation), males and females, heads and bodies, were collected at 2, 12, 24, 48, and 96 hours post-emergence from pupae to adults. All time points were collected at the same time of the day (from 8:30 to 10:30 am). The mosquitoes were immobilized by chilling on ice and then dissected to obtain head and body. Two pools of five mosquitoes from each time point (2, 12, 24, 48, and 96h post-emergence) of three independent experiments were collected. The samples were immediately placed on TRIzol Reagent (Thermo Fisher Scientific) and the tissues were homogenized with pestles. For each sample, 1μg of RNA was treated with DNAse I (Thermo Fisher Scientific) and cDNA was prepared using a High Capacity cDNA Reverse Transcription Kit (Thermo Fisher Scientific) according to the manufacturer's instructions. qPCR was performed in a StepOnePlus Real-Time PCR System (Thermo Fisher Scientific) with HOT FIREpol EvaGreen qPCR Mix Plus (Solis Biodyne) with a final volume of 10μL, using 2μL of the reagent, 0.1μM of each primer, and 5μL of cDNA (1:50). The efficiency of the primers was established through calibration curves and determined from a slope. The primers used for this analysis are provided in Table S6. Raw cq values were used in OLIVER, GeNorm, NormFinder, RefFinder, and BestKeeper to validate the stability of the gene expression.
